# Supplementary material for: Regrafting submillimeter-scale ferromagnetic soft continuums
Source: Nat Commun. 2025 Jul 31;16:7023. doi: 10.1038/s41467-025-60928-6 (PMC12313971; doi:10.1038/s41467-025-60928-6)
Supplement: Supplementary file 1 — Supplementary Information [file 41467_2025_60928_MOESM1_ESM.pdf]

# Supplementary Materials for

## Regrafting Submillimeter-scale Ferromagnetic Soft Continuums

**Yang Yang[1†], Wentao Shi[1†], Boguang Yang[2], Tiandi Xiong[2], Zhong Alan Li[2], Hongliang Ren[1]***

**[1] Department of Electronic Engineering, The Chinese University of Hong Kong, Hong Kong SAR, 99077, China.**

**[2] Department of Biomedical Engineering, The Chinese University of Hong Kong, Hong Kong SAR, 99077, China.**

*** Corresponding author: hlren@cuhk.edu.hk**

**[†] These authors contributed equally to this work.**

**The PDF file includes:**

**Texts**

Section S1. ETAMs preparation and fabrication
Section S2. Microscopic analysis
Section S3. Stretching and VSM tests
Section S4. Graded stiffness at N-tips
Section S5. Numerical setups
Section S6. Biocompatibility
Section S7. Experimental setups
Section S8. ETACs with/without optic fibers
Section S9. Detailed results of complex aerodigestive tract navigations
Section S10. Heat safety
Section S11. Motivations and creativity
Section S12. Animal trial
Section S13. In vivo grasper assembling
Section S14. Movement precision of ETACs
Section S15. Temperature monitoring
Section S16. Lubrication needs
Section S17. Softening and melting point
Section S18. Heating area issues
Section S19. Magnetization change issue
Section S20. Detailed explanation of self-division
Section S21. Self-alignment during the mergence process
Section S22. Predictions of self-merged ETAM-devices
Section S23. Softening effect by preheating
Section S24. In vivo meshing
Section S25. Mobility of separated sub-ETACs and their alignment strategies
Section S26. Self-division strategies and their stability analysis
Section S27. Potential biomedical scenarios and their corresponding required actuation distances

**Figures**

Fig. S1. Illustration of ETAMs preparation

Fig. S2. Fabrications of ETACs. (A) Fused extrusions of P-regions and N-ETAMs. (B) Manually fused extrusions of ETAMs for P-regions and N-regions. (C) Printed ETACs in a flexible mold, which can be easily demolded. (D) The printed ETACs can respond to the EPM field

Fig. S3. Experimental setups of SEM observations

Fig. S4. SEM image of P-regions (PCL) and surface element scanning result

Figs. S5-S14. Detailed SEM results with respect to P, F, and N-ETAMs

Fig. S15. Result of ETAMs stretching test

Fig. S16. Dimensions of ETAMs stretching sample

Fig. S17. Strain-stress relation of HMA-60%-F. Elastic deformation, plastic deformations, and failure were observed

Figs. S18-S23. Detailed results of stretch tests

Fig. S24. B-H curves of ETAMs were tested by a vibrating sample magnetometer (VSM) with respect to different MNP types and fractions

Figs. S25-S35. Detailed results of bending tests

Fig. S36. Numerical setup of continuums' bending behaviors in gradient fields

Fig. S37. CCK-8 results showing the viability of cells co-cultured with magnetite- and neodymium-based ETAMs for 24, 48, and 72 hrs. N = 5 biological replicates. *, $p \leq 0.05$ (two-way ANOVA).

Fig. S38. Live/dead staining results. (A) Representative images showing the live (green) and dead (red) cells after 72 h of co-culture with ETAMs. N = 5 biological replicates. (B) Quantitative analysis of the mean fluorescence intensity (MFI) of Calcein acetoxymethyl ester (Calcein-AM, for live cells) and Propidium Iodide (PI, for dead cells). N = 3 biological replicates, with 3 fields randomly selected for each biological replicate for MFI analysis. **, $p \leq 0.01$; ****, $p \leq 0.0001$ (one-way ANOVA).

Fig. S39. Length-width ratio effect on bending tests under Helmholtz coil control

Fig. S40. Graded stiffness effect on bending tests under EPM fields

Fig. S41. Experimental setup for "continuums-carriers" demonstration

Fig. S42. Experimental setup for complex aerodigestive tract navigations

Fig. S43. Bronchi phantom applied for navigation tests

Fig. S44. Experimental setup for thermal effects on bending performances

Fig. S45. Experimental setup of demagnetization tests

Fig. S46. Experimental setup of local heating test

Fig. S47. Experimental setup of thermodynamic experiment on induction heatings

Fig. S48. Ex vivo intestine was utilized to conduct the ESD demonstration

Fig. S49. Bending performances of an optic-fiber equipped ETAC (Continuum diameter: 0.8 mm)

Fig. S50. Endoscope-equipped ETAC captured bronchi images

Fig. S51. Navigation results of complex aerodigestive tract practices

Fig. S52. Heating temperature-time relation for biomedical uses. Regrafting is proven to be safe for in vivo uses

Fig. S53. Necessity of self-division from fundamental scientific and engineering perspectives

Fig. S54. In vivo ablation on a hairless mouse

Fig. S55. Ex vivo porcine stomach tissues- control group

Fig. S56. Ablated ex vivo porcine stomach tissues- 10 s-ablation groups

Fig. S57. Ablated ex vivo porcine stomach tissues- 30 s-ablation groups

Fig. S58. Ablated ex vivo porcine stomach tissues- 60 s-ablation groups

Fig. S59. Mouse stomach tissues- control groups
Fig. S60. Ablated in vivo mouse stomach tissues
Fig. S61. Ablated in vivo mouse cardia (lower esophagus) tissues
Fig. S62. In situ assembly
Fig. S63. Movement precision test of ETAM-ESUs.
Fig. S64. Empirical temperature monitoring method.
Fig. S65. Comparison test between uniform modulus ETAC, uniform modulus ETAC with lubrication, graded-stiffness
Fig. S66. Qualitative comparison tests on the heat dissipation effect of lubrication.
Fig. S67. Relation between ETACs' stiffness, MNP proportions, ETAC types, and temperature.
Fig. S68. Two methods to reduce heating area: customizing RF coil geometry and utilizing heat distribution center. Created in BioRender. Yang, Y. (2025) https://BioRender.com/7idnddp
Fig. S69. Magnetic strength comparisons
Fig. S70. Comparisons between the manual and magnetic division approach.
Fig. S71. Self-alignment of separated segments.
Fig. S72. Actuation prediction of self-merged ETACs.
Fig. S73. The phase transition process of 3-states ETAMs and 4-states-ETAMs.
Fig. S74. In vivo meshing.
Fig. S75. Potential applications of in vivo meshing demonstration.
Fig. S76. Biomedical application scopes of ETACs requiring passing through narrow bottlenecks. Created in BioRender. Yang, Y. (2025) https://BioRender.com/pbrbpao
Fig. S77. In vivo meshing underwater was successfully realized, proving that the presence of the body fluid or lubricants also allows the self-mergence.
Fig. S78. Experimental setup
Fig. S79. Experimental results of separated ETACs' mobility characteristics and alignment strategy
Fig. S80. Self-division strategies including: (A) pulling division, (B) bending division, and (C) twisting division.

**Tables**

Table S1. Stretch results for all studied cases
Table S2. Case table of stiffness adjustment
Table S3. Comparisons between regraftable FSCs and existing well-accepted biomedical devices
Table S4. Case table of the experimental design
Table S5. Potential biomedical scenarios and their corresponding required actuation distances.

**Section 1. ETAMs preparation and fabrication**

Here, we introduce the detailed fabrication of the upgraded ETAMs used in this work compared with our previous study (*1*). In the previous work, the hot melt adhesive (HMA) was mainly focused on providing more fabrication convenience. However, HMA is not considered to be applied for biomedical applications due to its unsatisfactory biocompatibility. Here, we utilize PCL as the main base material for the targeted continuum robot application. As we mentioned in the main text, in this work, we further classify the ETAMs into three subfamilies: P-regions, N-ETAMs, and F-ETAMs which are made by pure PCL, PCL+NdFeB, and PCL+$Fe_3O_4$.

A hot platform was applied to provide stable temperature control for the thermal mixing process. PCL particles (150 μm, Ruixiang Plastics Inc.), neodymium MNPs (5 μm, Magnequench Co., Ltd.), and magnetite NPs (48 μm, Leber Inc.) were selectively weighted and premixed in a glass culture dish, which was heated by a heating platform (Yarun Inc.). Heating temperatures varied from 160-200 ℃ with respect to different PCL particle types (Fig. S1). Regarding the thermal mixing, the glass culture dish was preheated for ~5 minutes on the heating platform, and all weighted particles were added and stirred for another 5 minutes. After cooling to the ambient temperature, ETAMs were ready to use. It should be noted that the so-called "ready-to-use" refers to using fabricated ETAC in its enhanced stiffness state. It needs 8-10 hrs to cure into the hardened state.

We demonstrated the fabrication of ETACs in Supplementary Movie S1. Regarding the manual printing, glue guns were prepared to install the fabricated ETAMs strips. The temperature of the glue guns was set to 200 ℃, which can extrude ETAMs fluids smoothly. To distinguish different ETAM sub-families, we mixed fluorescent phosphors during the ETAMs fabrication (Fig. S2). Green and yellow refer to P-regions and N-ETAMs, respectively. A silicon mold with a straight groove was prepared by Ecoflex 0010 (Smooth-on Inc.). Along the groove, ETAM fluids can be extruded onto the mold and flattened by a scraper. After ~5 minutes, the solidified ETAMs continuum can be obtained.

Regarding the connection between two ETAMs continuums, either directly printing different ETAMs in order for single-time fabrication or bonding two fabricated continuums by aligning and heating are workable, according to our tests.

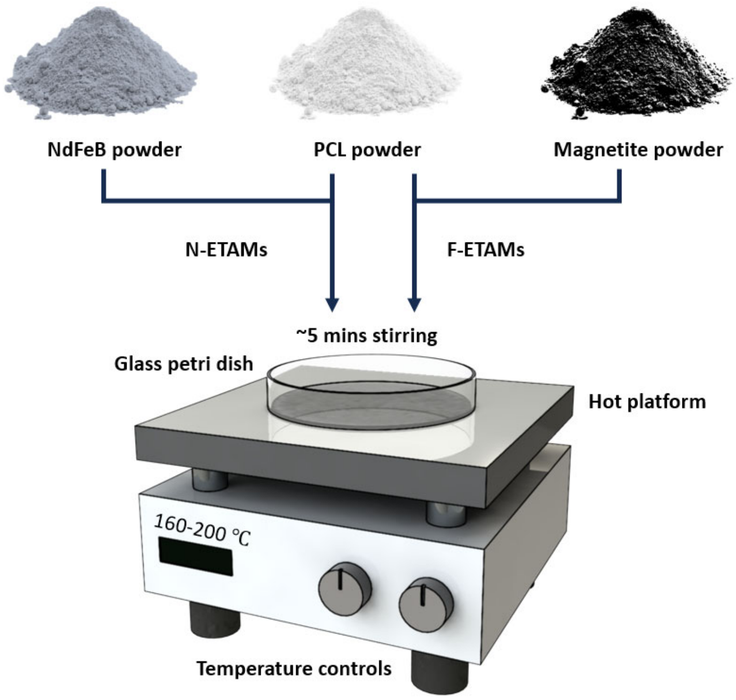


**Fig. S1. Illustration of ETAMs preparation.**

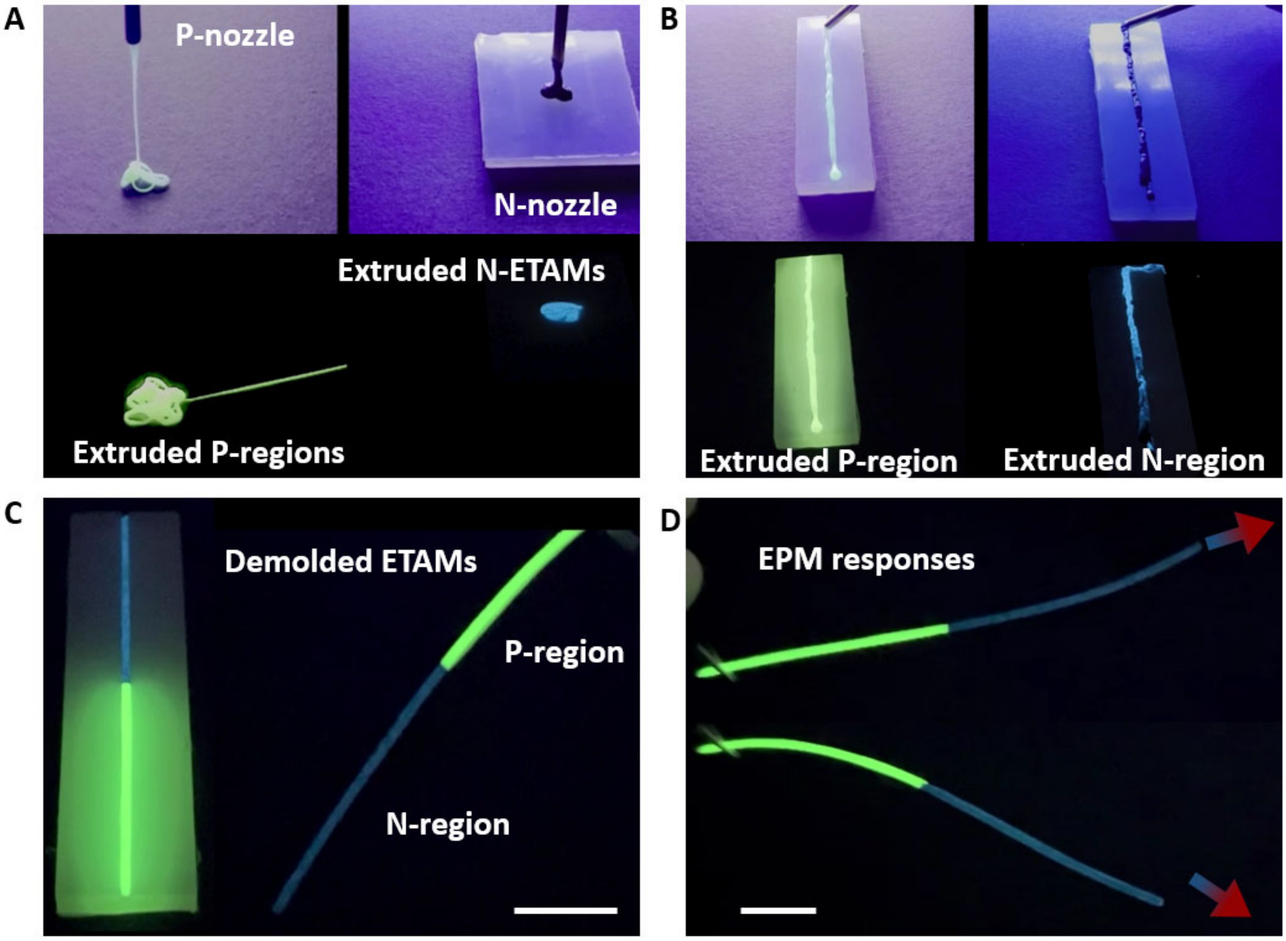


**Fig. S2. Fabrications of ETACs. (A) Fused extrusions of P-regions and N-ETAMs. (B) Manually fused extrusions of ETAMs for P-regions and N-regions. (C) Printed ETACs in a flexible mold, which can be easily demolded. (D) The printed ETACs can respond to the EPM field. Scale bar: 1 cm.**

**Section 2. Microscopic analysis**

In this section, we list all recorded data as supplementary data. We conducted SEM observations on the ETAMs samples. N-ETAMs and F-ETAMs were further conducted surface element scanning to locate MNPs clusters. For a single case, we provide SEM image and element distribution maps with respect to C element, O element, Fe element, and Nd element.

We should especially point out that, in Fig. S14, slight Nd elements can be observed in P-region. This is caused by fabrication imperfections. However, side effects of such imperfection have not been observed during the practical tests and predictions. We also note that the platinum element was observed in SEM images due to the platinum coating for sample preparations. It does not exist in real cases for applications.

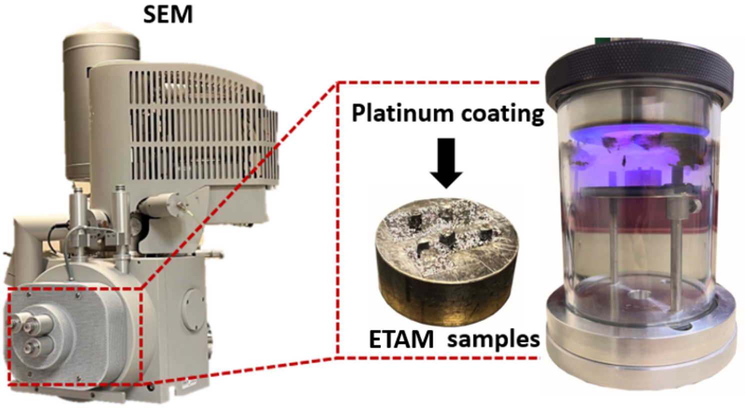


**Fig. S3. Experimental setups of SEM observations.**

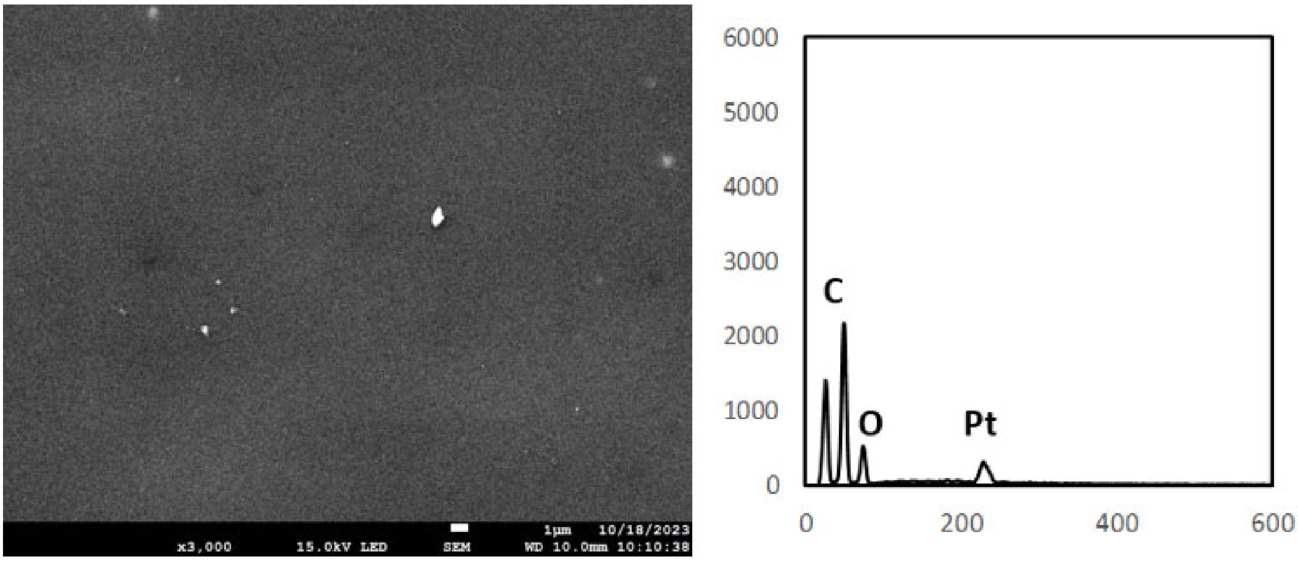


**Fig. S4. SEM image of P-regions (PCL) and surface element scanning result.**

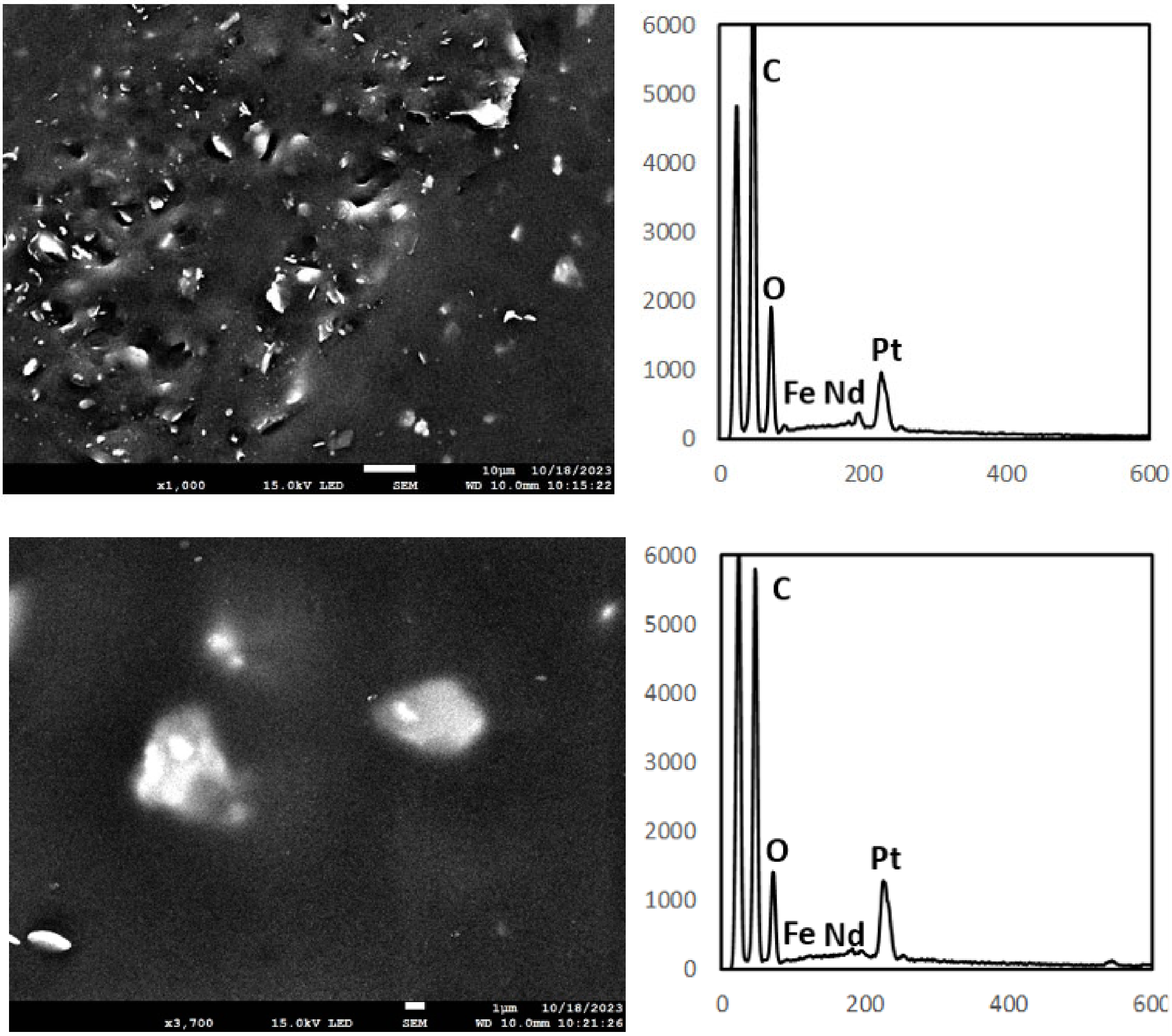


**Fig. S5. SEM images of N-ETAMs (80% NdFeB-PCL-12% Wax) and surface element scanning result.**

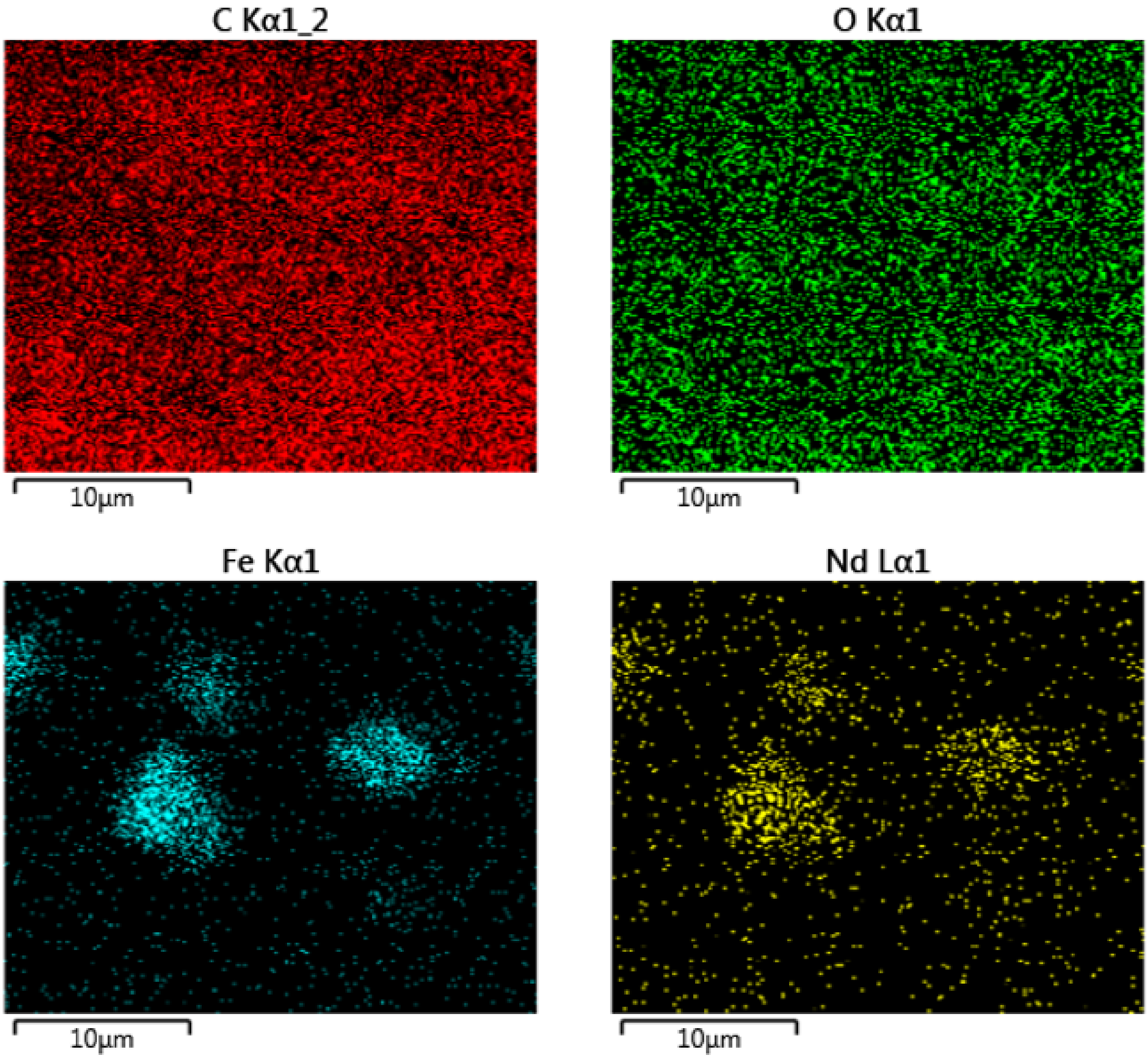


**Fig. S6. Element scanning map of N-ETAMs (80% NdFeB-PCL-12% Wax).**

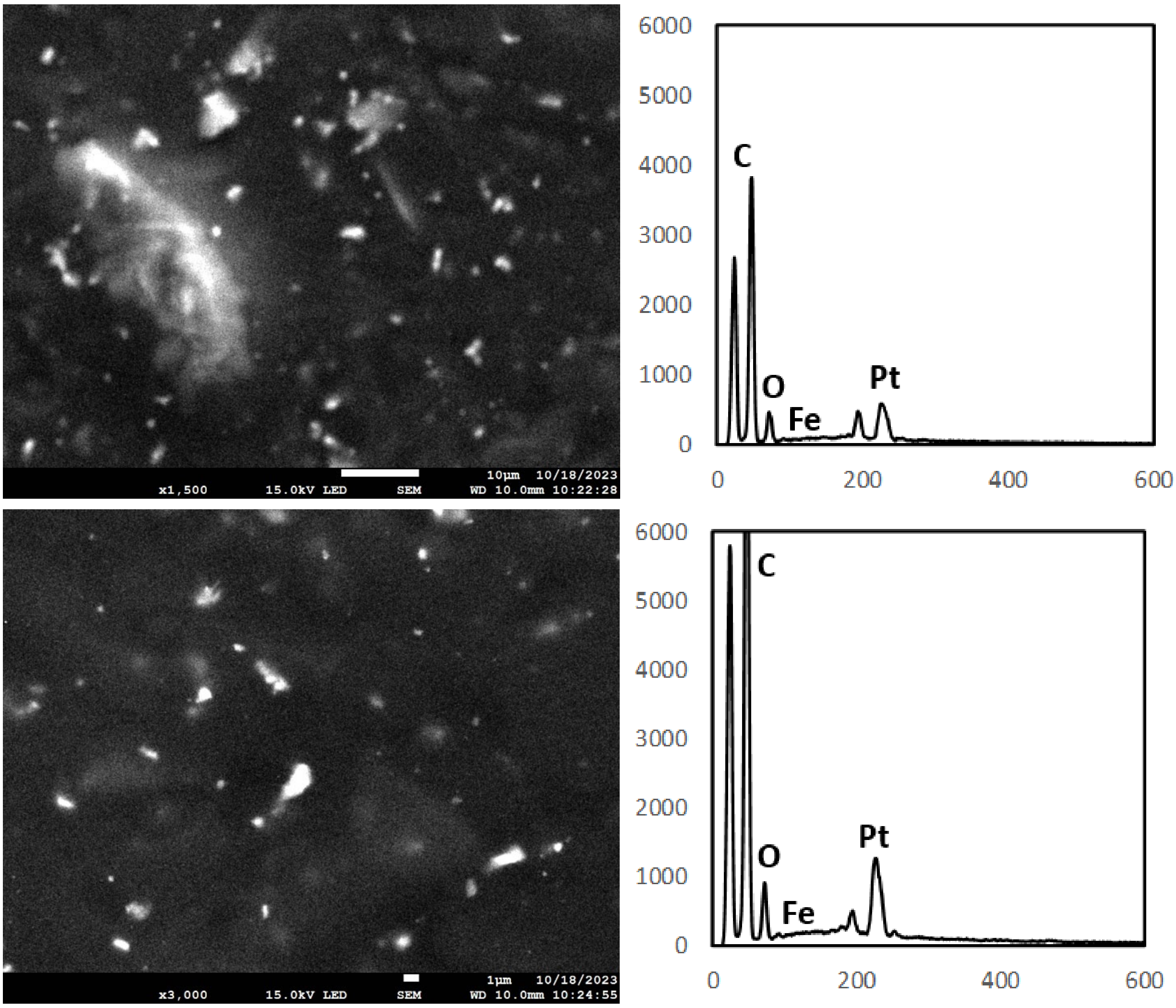


**Fig. S7. SEM images of F-ETAMs (80% $Fe_3O_4$-HMA) and surface element scanning result.**

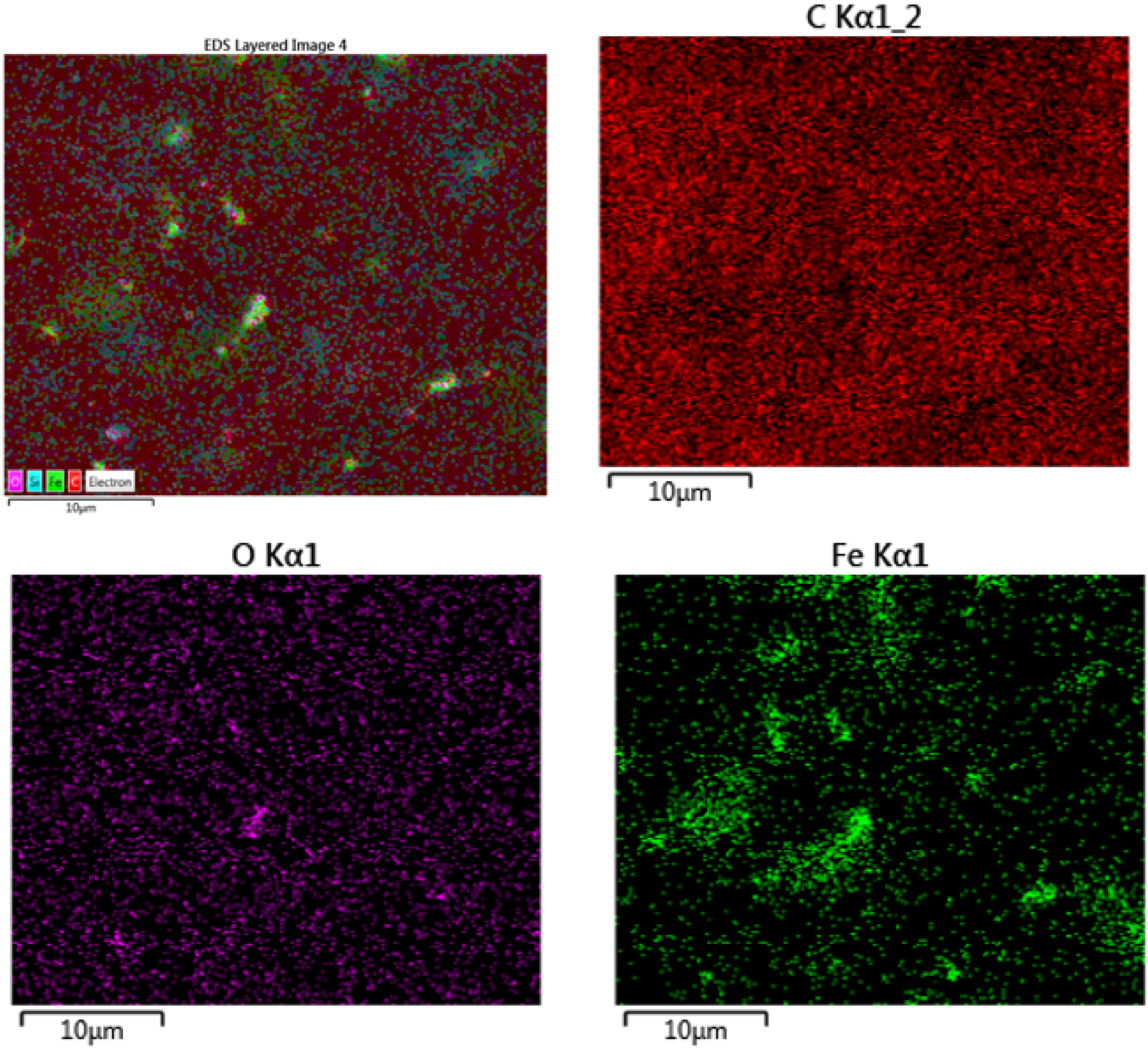


**Fig. S8. Element scanning map of F-ETAMs (80% $Fe_3O_4$-HMA).**

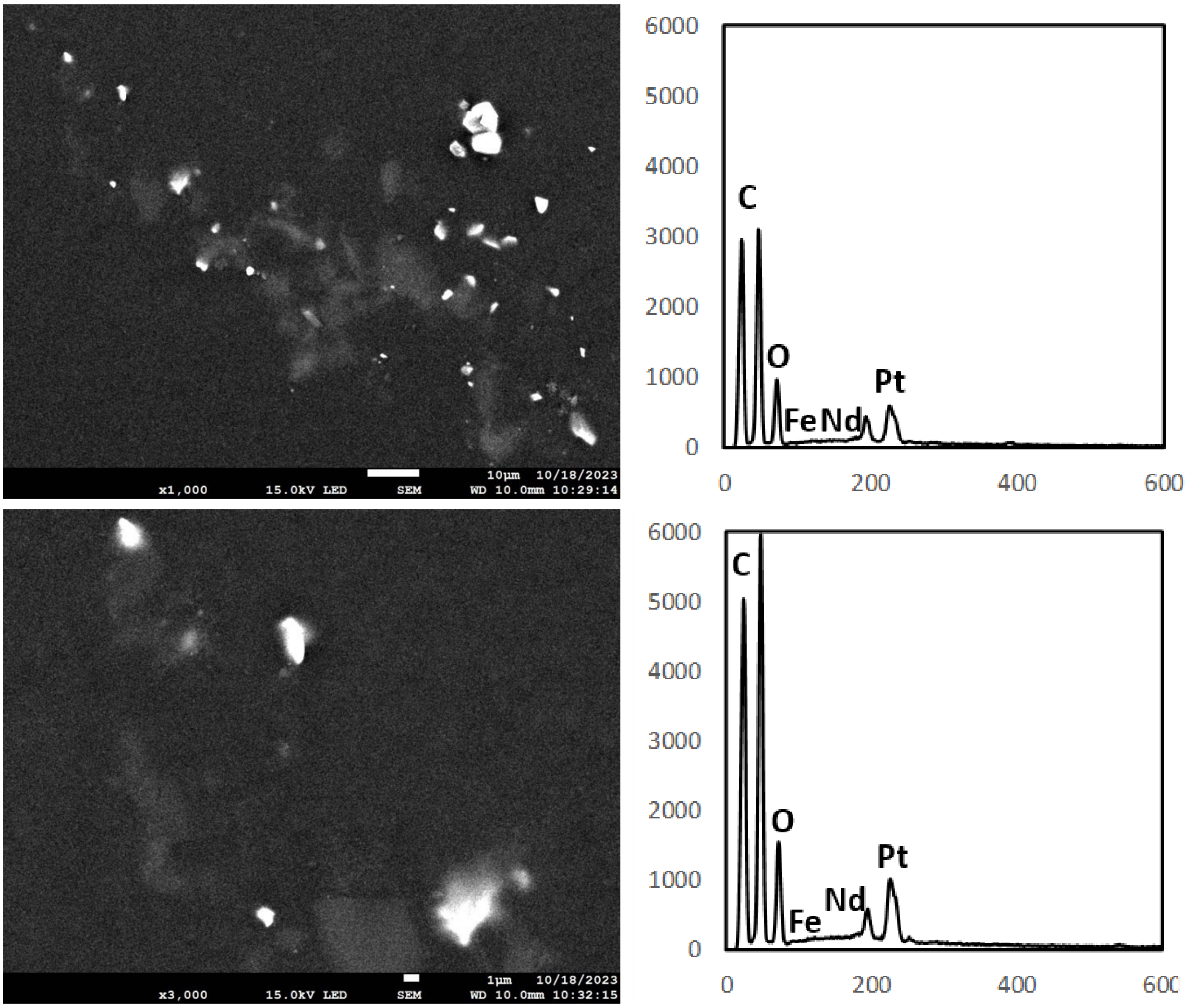


**Fig. S9. SEM images of N-ETAMs (80% NdFeB-PCL) and surface element scanning result.**

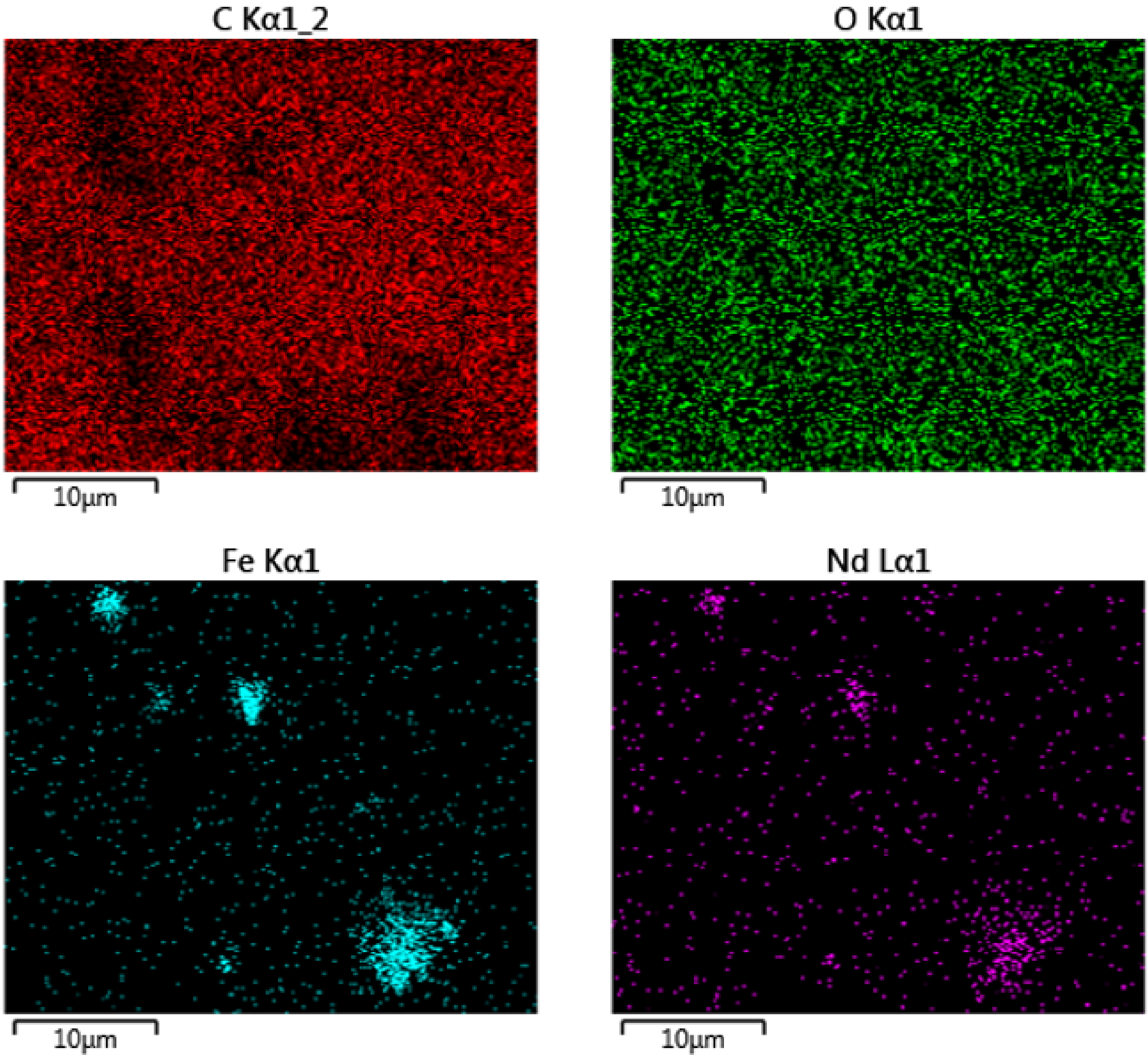


**Fig. S10. Element scanning map of N-ETAMs (80% NdFeB-PCL).**

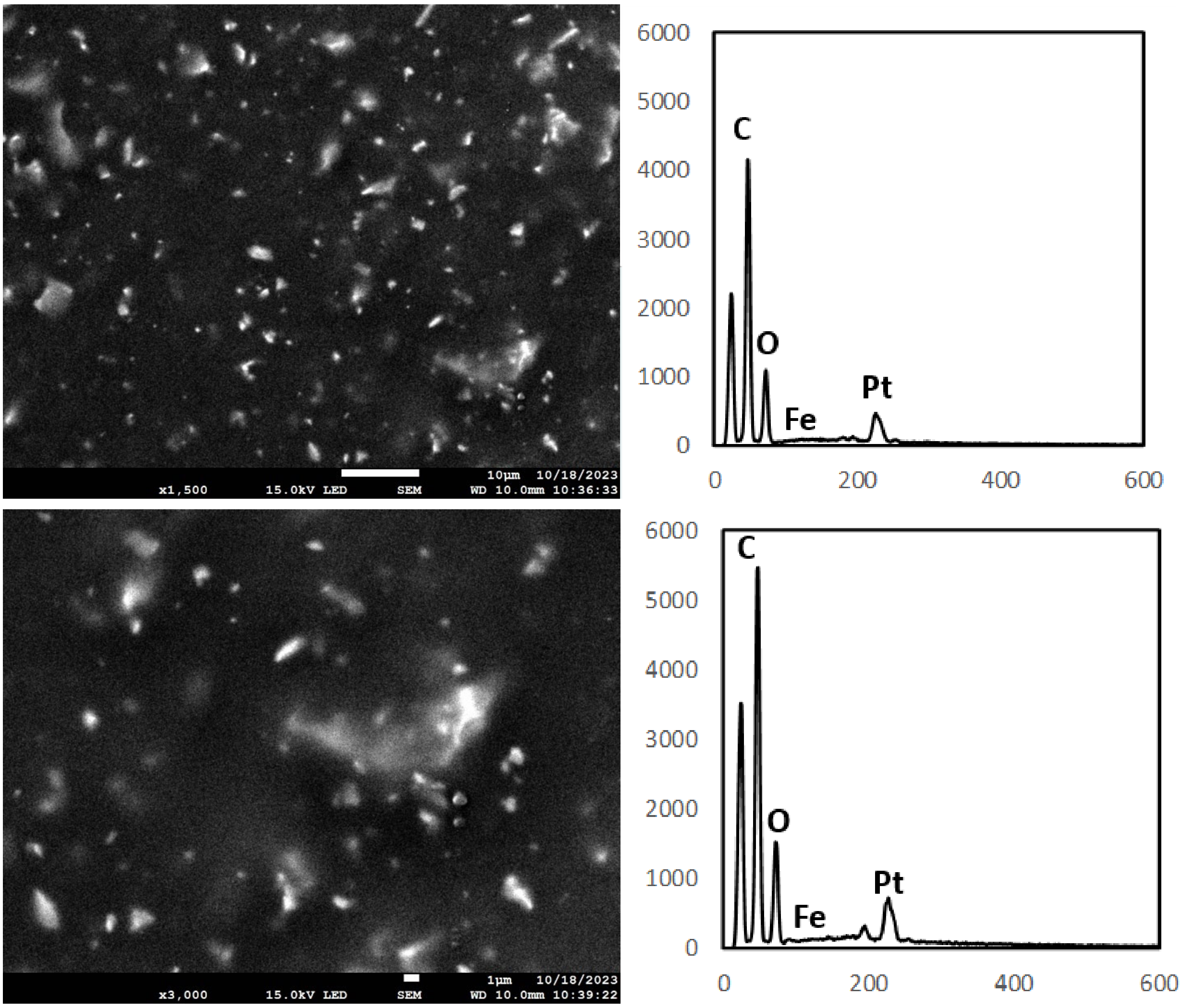


**Fig. S11. SEM images of F-ETAMs (80% $Fe_3O_4$-PCL) and surface element scanning result.**

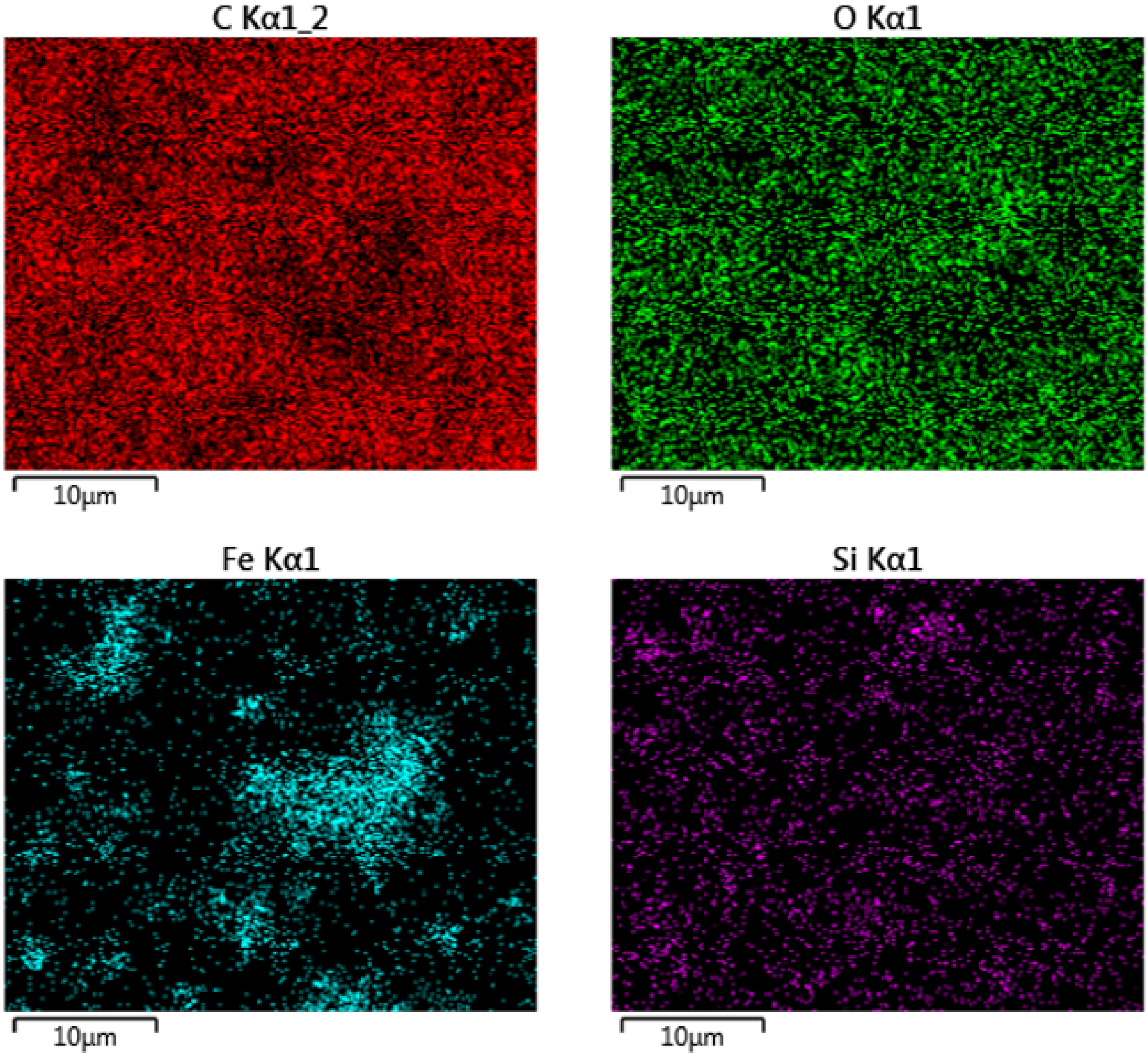


**Fig. S12. Element scanning map of F-ETAMs (80% $Fe_3O_4$-PCL).**

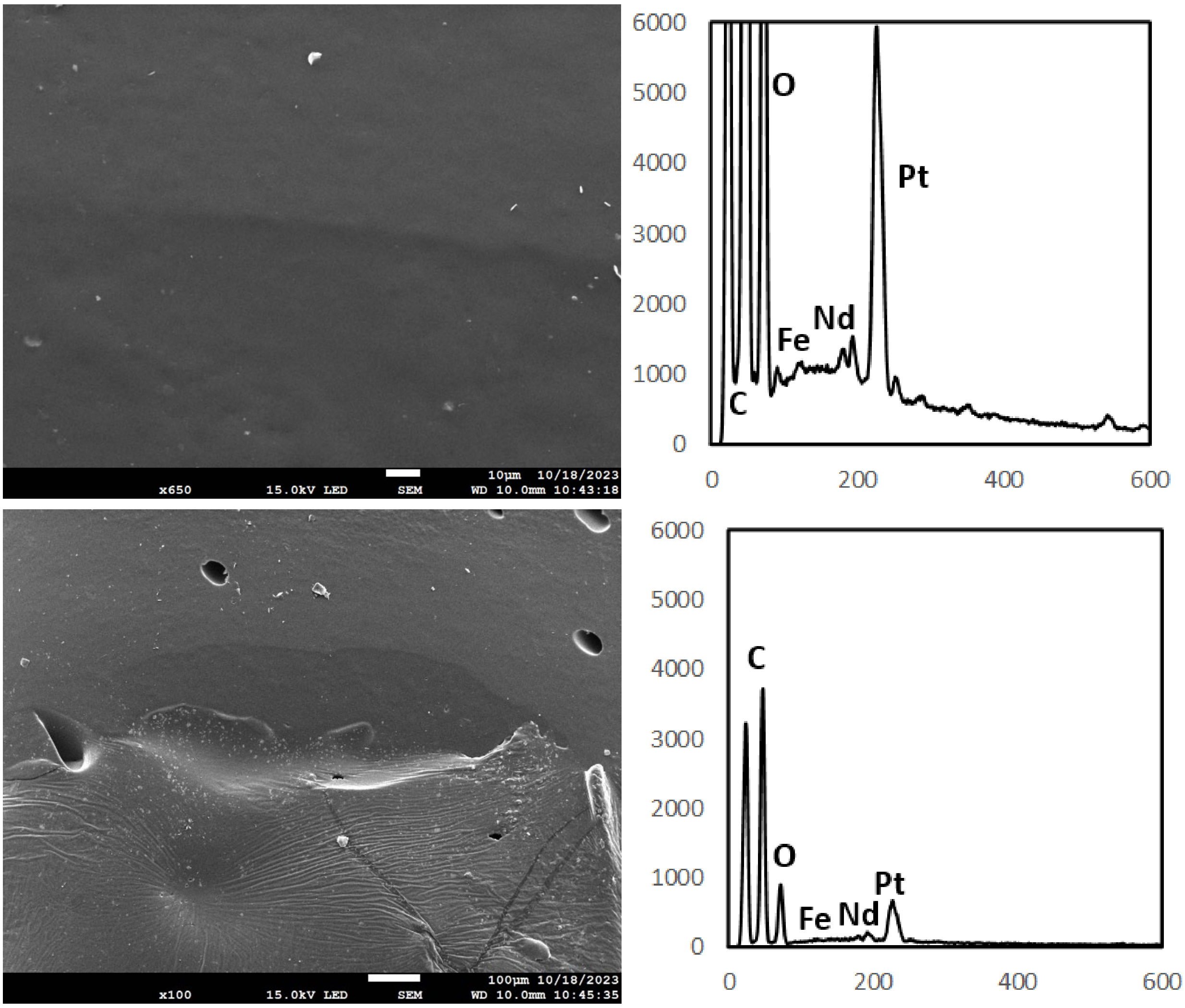


**Fig. S13. SEM images of P-N connection region and surface element scanning result.**

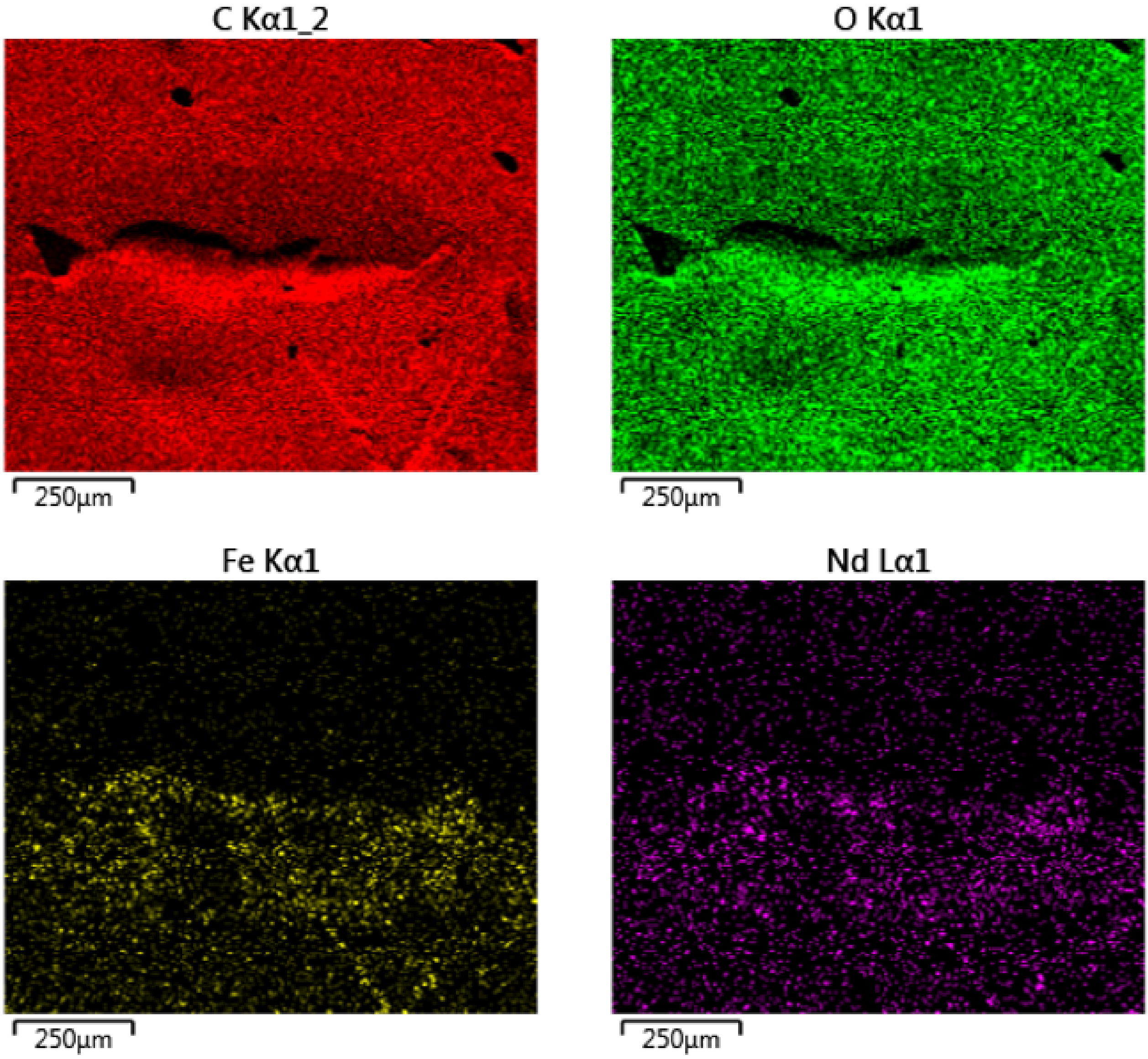


**Fig. S14. Element scanning map of P-N connection region.**

**Section 3. Stretching test**

This section provides recorded data as supplementary data for stretching and VSM tests.

Elasticity of ETAMs applied at the tip. In addition to PCL, the commercially available hot melt adhesives (HMA) were also applied as the alternative base material for ETAMs. We note that the biocompatibility of HMA was not validated for biomedical uses, which, however, can be applied to non-living involved scenarios (e.g., structure repair in narrow crevices). Wax was also considered to further enhance the flowability of ETAMs in the fluid state. Additive fraction refers to the mass ratio between MNPs and base materials: Max., Mid., and Min. refer to 100%, 80%, and 60%, respectively. Max., Mid., and Min. of the wax amount refers to 0%, 6%, and 12% of base materials mass. We should note that the wax-mixed case was only tested in the stretch experiment, which did not apply to all other cases in our work. Regarding ETAMs' elasticity in the elastomer state, the PCL-F, PCL-N, commercially available hot melt adhesive (HMA)-F, HMA-N, PCL-wax-F, and PCL-wax-N are tested (Fig. S14). The mass fraction between MNPs and base materials varied from 60%-100%.

These materials were tested with Young's modulus of 10-30 MPa, which showed satisfactory elasticity for continuum uses. The magnetization properties of the ETAMs with different MNP mass fractions were tested using a vibrating sample magnetometer (VSM, Fig. 2C).

In Figs. 18-23, the figure legends "1", "2", and "3" refers to the order listed in Table S1.

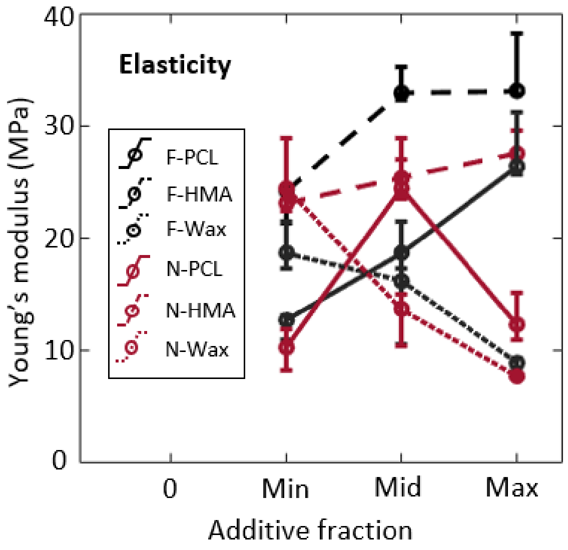


**Fig. S15. Result of ETAMs stretching test.**

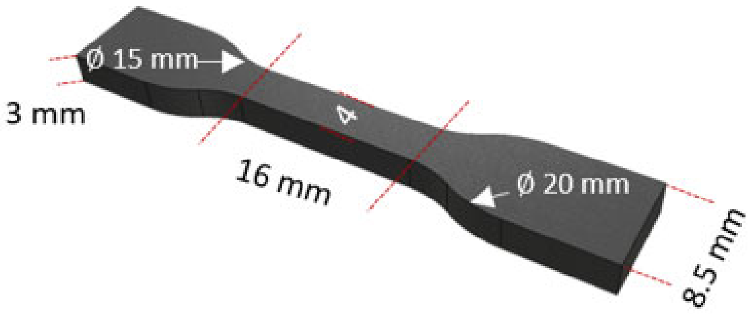


**Fig. S16. Dimensions of ETAMs stretching sample.**

**Table. S1. Stretch results for all studied cases.**

| Cases (Base material-Mass ratio-MNP type) | Young's modulus -1 (MPa) | Young's modulus -2 (MPa) | Young's modulus -3 (MPa) |
|---|---|---|---|
| PCL-60%-F | 12.7 | 13.1 | 10.9 |
| PCL-80%-F | 21.5 | 17.3 | 18.7 |
| PCL-100%-F | 31.2 | 25.6 | 26.3 |
| PCL-60%-N | 8.2 | 10.2 | 11.8 |
| PCL-80%-N | 28.9 | 23.9 | 24.4 |
| PCL-100%-N | 10.9 | 15.1 | 12.3 |
| HMA-60%-F | 24.2 | 21.3 | 24.2 |
| HMA-80%-F | 32.9 | 32.2 | 35.1 |
| HMA-100%-F | 32.7 | 38.2 | 33.1 |
| HMA-60%-N | 22.4 | 24.3 | 23.1 |
| HMA-80%-N | 26.9 | 23.4 | 25.3 |
| HMA-100%-N | 27.5 | 29.5 | 26.9 |
| PCL-60%-F-6%-Wax | 18.9 | 10.5 | 16.1 |
| PCL-60%-F-12%-Wax | 8.1 | 8,9 | 9.3 |
| PCL-60%-N-6%-Wax | 13.8 | 14.9 | 10.4 |
| PCL-60%-N-12%-Wax | 7.5 | 7.7 | 7.7 |

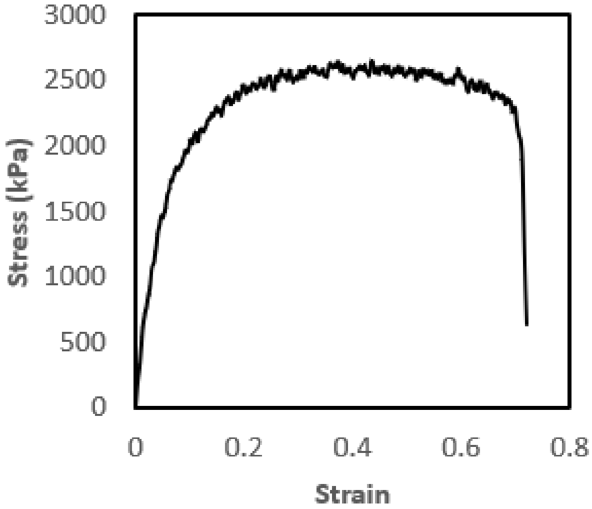


**Fig. S17. Strain-stress relation of HMA-60%-F. Elastic deformation, plastic deformations, and failure were observed.**

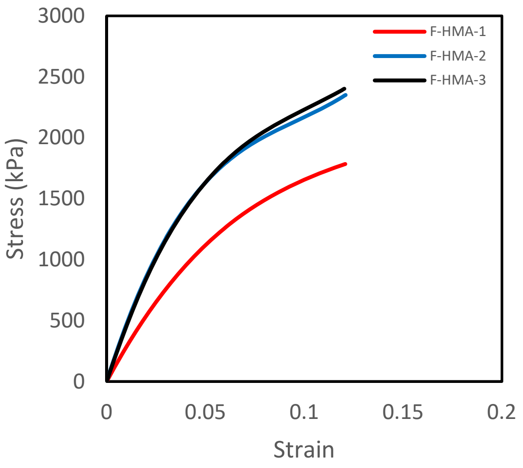


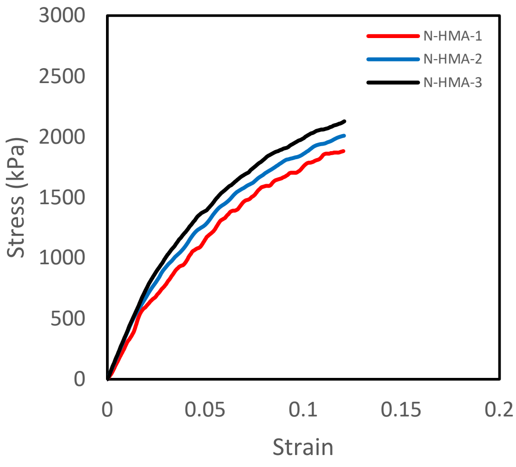


**Fig. S18. Stretch Results of Fe3O4-HMA**

**Fig. S19. Stretch Results of NdFeB-HMA**

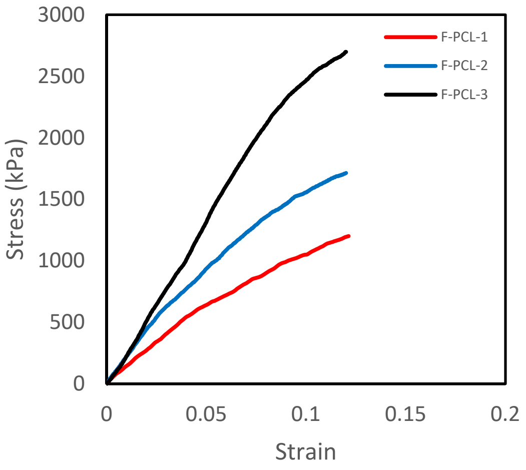


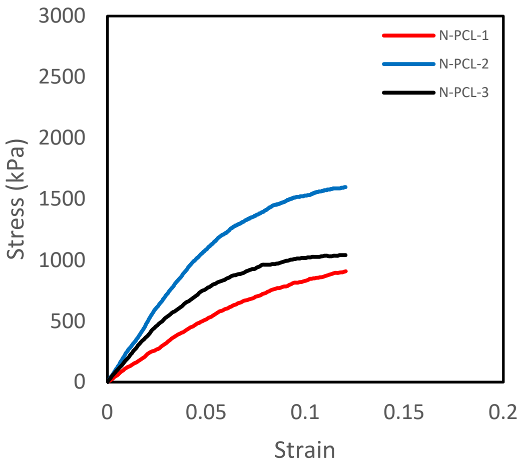


**Fig. S20. Stretch Results of Fe3O4-PCL**

**Fig. S21. Stretch Results of NdFeB-PCL**

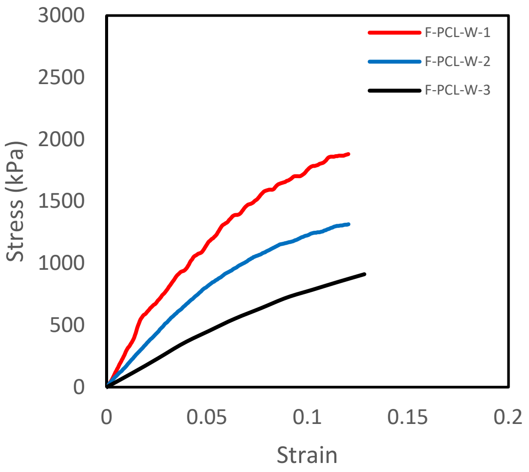


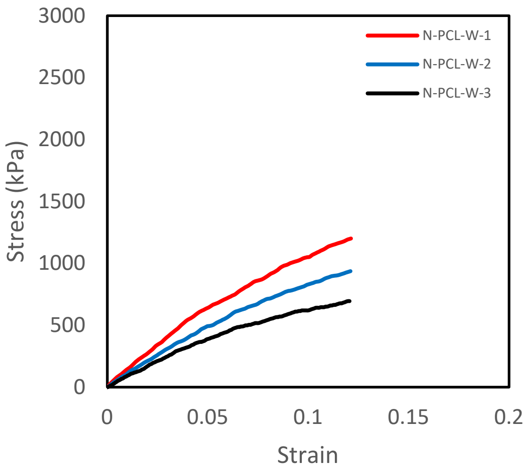


**Fig. S22. Stretch Results of Fe3O4-PCL-Wax**

**Fig. S23. Stretch Results of NdFeB-PCL-Wax**

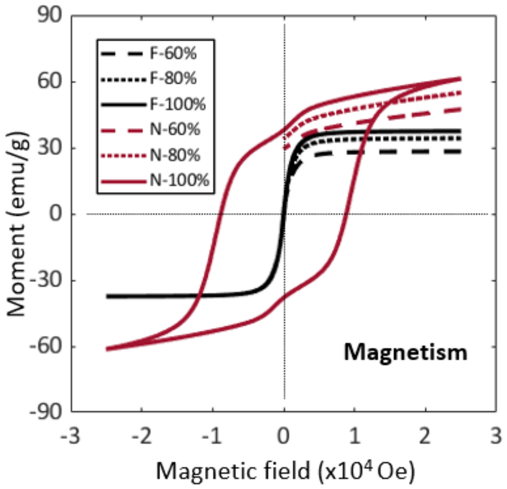


**Fig. S24. B-H curves of ETAMs were tested by a vibrating sample magnetometer (VSM) with respect to different MNP types and fractions.**

**Section 4. Graded-stiffness at N-tips**

This section explains fabrication techniques and bending tests we conducted to achieve the graded stiffness.

Commercially available PCLs have the same material properties but different initial stiffness. Using this property, we prepared two types of PCL powders with different initial stiffness, defined as soft PCL and rigid PCL. By giving different mixing ratios between the soft and rigid PCL powders under the same NdFeB proportion, the bending response can be varied due to the stiffness differences. As we mentioned in the main text, according to the practical tests, by connecting the flexible tip materials (10-30 MPa) to the rigid P-regions (more than 50 MPa), ETACs were found with connection failures and inefficient stress transitions due to the stiffness discontinuity. It reveals that the continuum tip can be further functionally divided to fulfill the flexibility and rigidity with a smooth stiffness transition. Therefore, we divided the continuum tip into the tip, middle, and bottom regions. We prepared 1:1, 10:1, and 6:1 (soft: rigid) for the tip region, 5:1, 4:1, and 3:1 for the middle region, and 2:1, 1:1, and 7:10 for the bottom region. The detailed case table is shown in Table S2.

Then, we investigated the graded stiffness effects by separately adjusting the stiffness at the tip, middle, and bottom regions. The bending results are shown in Figs. S25-35.

**Table S2. Case table of stiffness adjustment**

| Graded stiffness region | Mix ratio soft: rigid (“Name”) | PCL (Soft) (g) | PCL (Rigid) (g) | NdFeB (g) |
|---|---|---|---|---|
| Tip | 1:1 (“1/0”) | 10 | 0 | 20 |
| | 10:1 (“10”) | 9.09 | 0.91 | 20 |
| | 6:1 (“6”) | 8.57 | 1.43 | 20 |
| Middle | 5:1 (“5”) | 8.33 | 1.67 | 20 |
| | 4:1 (“4”) | 8 | 2 | 20 |
| | 3:1 (“3”) | 7.5 | 2.5 | 20 |
| Bottom | 2:1 (“2”) | 6.67 | 3.33 | 20 |
| | 1:1 (“1”) | 5 | 5 | 20 |
| | 7:10 (“0.7”) | 4 | 6 | 20 |

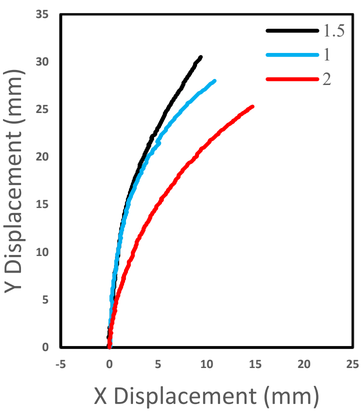


Fig. S25 . Bending Results of 1/0-3-change

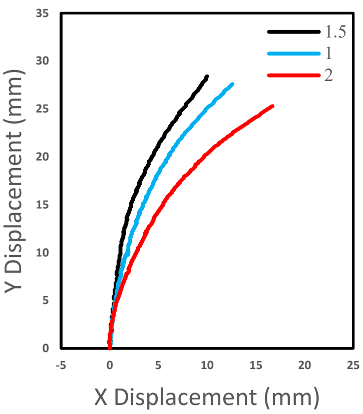


Fig. S26 . Bending Results of 1/0-4-change

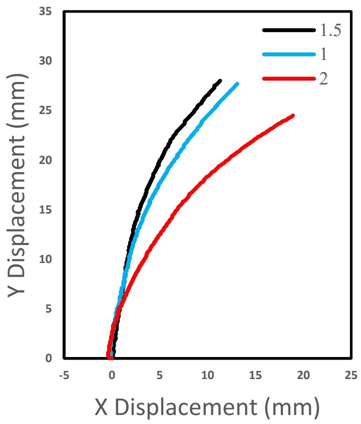


Fig. S27 . Bending Results of 1/0-5-change

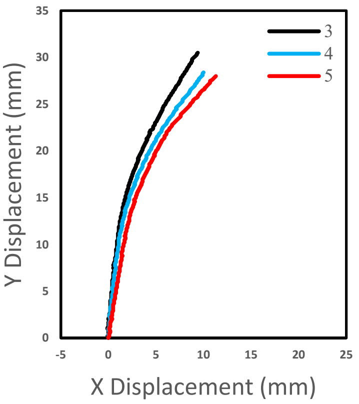


Fig. S28. Bending Results of 1/0-change-1.5

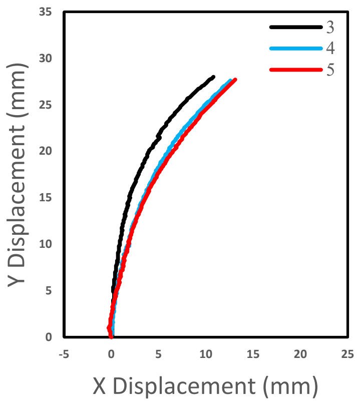


Fig. S29. Bending Results of 1/0-change-1

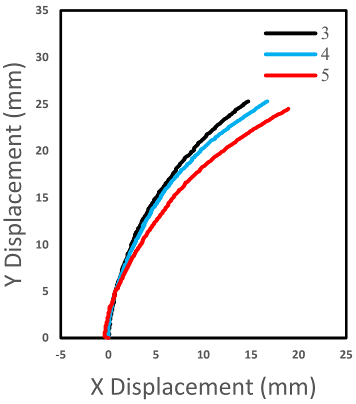


Fig. S30. Bending Results of 1/0-change-2

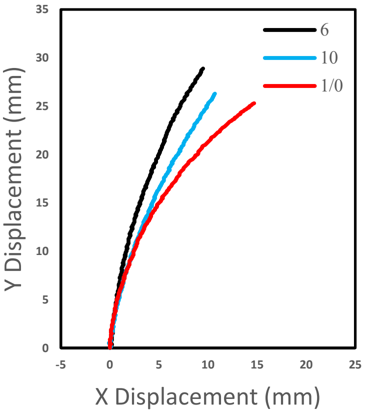


Fig. S31. Bending Results of change-3-2

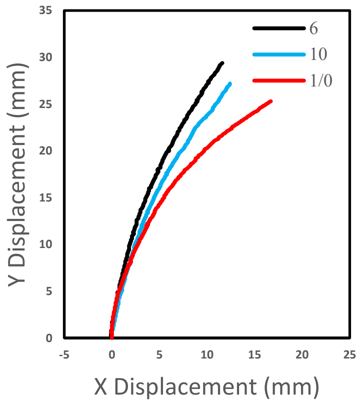


Fig. S32. Bending Results of change-4-2

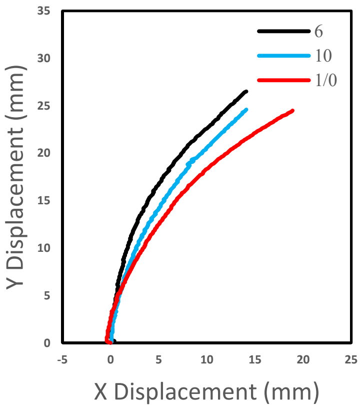


Fig. S33. Bending Results of change-5-2

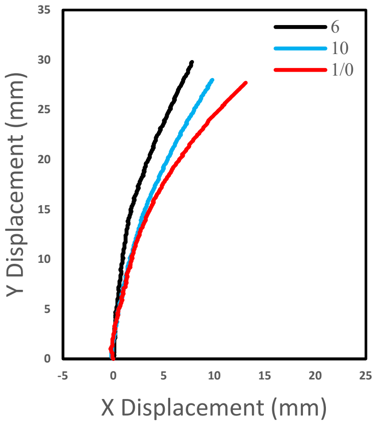


**Fig. S34. Bending Results of change-5-1**

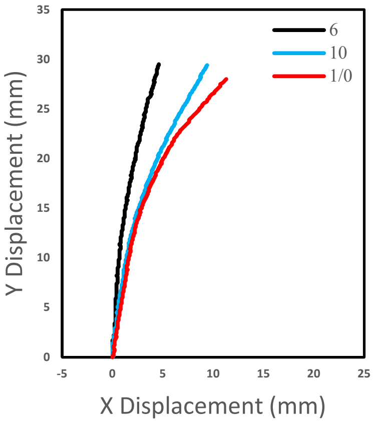


**Fig. S35. Bending Results of change-5-1.5**

**Section 5. Numerical setup**

In this section, we introduce the numerical model in detail. We built two numerical models with COMSOL Multiphysics to respectively simulate the ETACs' bending behaviors under gradient fields and uniform fields.

Regarding the gradient field cases (Fig. S36), a Hyperelastomer-magnetics coupled model was developed by applying the system coupling method. The geometric model contains an airfield and a continuum. The Neo-Hookean model was assigned to the continuum. It should be noted that the bending performances of classic elastomer and Hyperelastomer do not have obvious differences. This is because the continuum is subject to torque instead of stretching during bending motions. It is known that the Neo-Hookean model provides accurate hyperelastomeric descriptions when the strain is lower than 100%. Within this region, hyperelastomeric performances and elastomeric performances are similar. Fixed support was applied to a continuum's end as the boundary condition. The dynamic mesh technique was applied to adapt the mesh deformations during the bending. The residual flux density model was applied to describe the magnetization strength of the continuum, which was 24 mT. The gradient field was defined by the relative permeability model. The continuum and magnetic fields were interacted by the system coupling technique.

Regarding the uniform field case (Fig. S36), the geometry setup is the same as that of the gradient case. The difference mainly lies in the continuum-magnetic coupling strategy. Same as the common way to do so, we applied the Maxwell tensor at the opposite end of the fixed continuum's surface, instead of applying the system coupling technique. The magnetic field strength was defined by giving a magnetic flux density to the whole air region.

We should note that involving EPM fields enhances the magnetic strength that the common Helmholtz cannot reach. The Helmholtz coil we applied can generate a maximum magnetic field of ~24 mT, while the permanent magnet can generate more than 300 mT at its surface. However, we found slight differences when comparing bending performances under high-strength gradients and uniform fields. Given the high calculation consumption of the gradient field case (2~3 hrs for one case), we applied the uniform field model to describe the bending performances under high-strength gradient fields (30 seconds for one case). The gradient magnetic field strength value at the bent tip region was chosen as the uniform value.

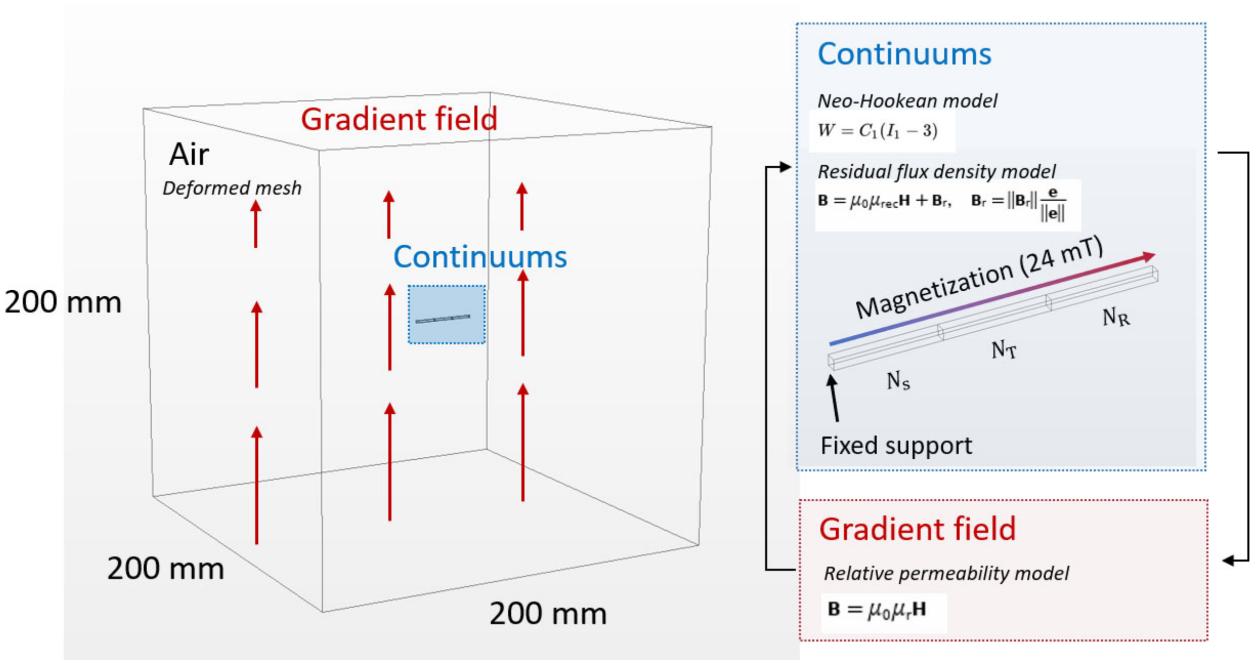

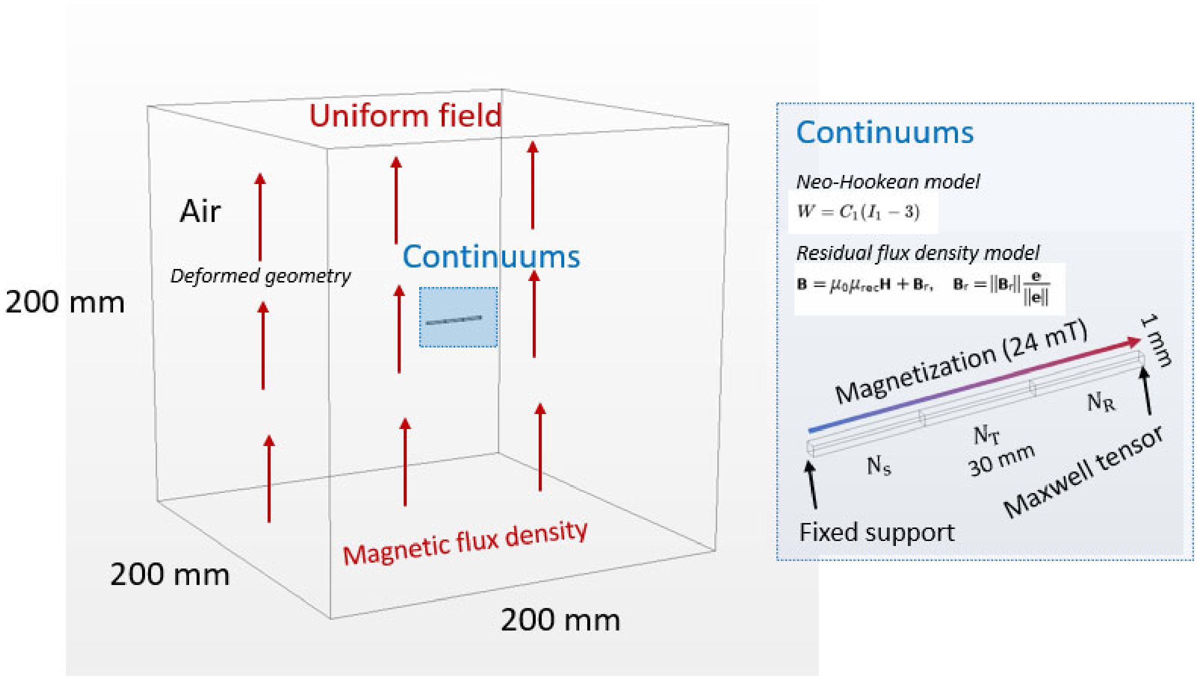


**Fig. S36. Numerical setup of continuums' bending behaviors in gradient and uniform fields.**

**Section 6. Biocompatibility**

In this section, we provide the detailed results of the biocompatibility tests.

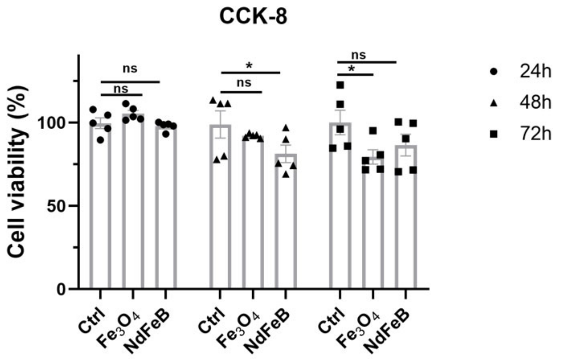


**Fig. S37. CCK-8 results showing the viability of cells co-cultured with magnetite- and neodymium-based ETAMs for 24, 48, and 72 hrs. N = 5 biological replicates. *, $p \leq 0.05$ (two-way ANOVA).**

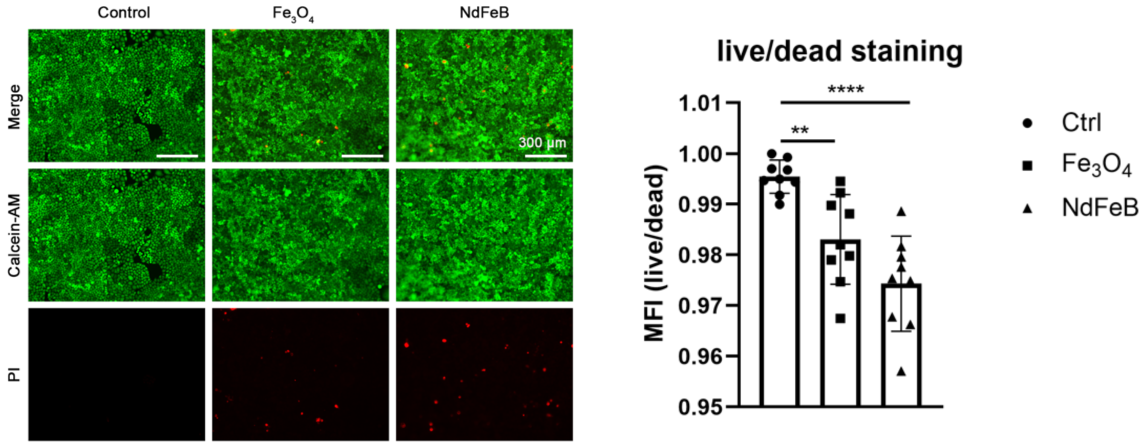


**Fig. S38. Live/dead staining results. (A) Representative images showing the live (green) and dead (red) cells after 72 h of co-culture with ETAMs. N = 5 biological replicates. (B) Quantitative analysis of the mean fluorescence intensity (MFI) of Calcein acetoxymethyl ester (Calcein-AM, for live cells) and Propidium Iodide (PI, for dead cells). N = 3 biological replicates, with 3 fields randomly selected for each biological replicate for MFI analysis. **, $p \leq 0.01$; ****, $p \leq 0.0001$ (one-way ANOVA).**

**Section 7. Experimental setup**

This section introduces all experimental setups adopted in the main text.

Uniform fields for bending tests. As shown in Fig. S39, a continuum holder was designed and 3D printed to fixed support under testing continuums. The holder was fixed inside the platform in a Helmholtz coil. The magnetic field was horizontally generated, which is vertical to the axial direction of the continuum. The camera was put on the top of the coil to capture the top view of the bent deformation. The captured image was post-processed with Mathematica (Wolfram Inc.). The image was cropped and binarized to record the outline of the deformed continuum.

Gradient bending tests. As shown in Fig. S40, the test system contains a permanent magnet (N52) and a continuum holder. The continuum was inserted into the install hole designed in the holder for fixed supports. Unlike the uniform fields test, the holder is straightly adjustable along the N direction of the magnet. The magnetic field was recorded by a magnetometer with respect to the distance to the magnet surface center. The undeformed continuum's tip was aligned to the center of the magnet surface.

Continuums carriers. As shown in Fig. S41, the dimensions for the channel and three objects are presented. The channel was put on a 5 mm acrylic board as the support platform. The permanent magnet was manipulated under the acrylic board to control the deformation.

Complex aerodigestive tract navigations. As shown in Fig. S42, a complex aerodigestive tract model was applied to conduct the navigation tests. The average inner diameter of the channels is ~2.5 mm. The phantom was put on a 5 mm acrylic board as the support platform. The permanent magnet was manipulated under the acrylic board to control the deformation.

The bronchi phantom applied to conduct navigation tests is shown in Fig. S43.

Thermal effect on bending performances. As shown in Fig. S44, all prepared continuums were first put in an oven under 24, 36, 48, and 60 degrees Celsius heating for 30 mins. After heating, the bending performances were tested in the Helmholtz coil. Then, these tested continuums were put in ambient (20 ℃) for 1 hr curing and retested. Repeating the curing-test loop 10 times, we observed that the curing process could be completed.

The experimental setup of the demagnetization test is presented in Fig. S45.

Local heating experiments. As shown in Fig. S46, the experimental setup of the local heating tests contains an induction heater, an acrylic platform, a silicon rubber mold with a straight groove, and an N-P-F-P-N continuum. The induction heater was constrained to move along the continuum's axial direction.

Thermodynamic experiments on induction heatings. As shown in Fig. S47, the experimental setup of the induction heating contains a holder, an induction heater, and ETAMs sample. The holder was designed with different holes at various heights to the coil to insert the sample. Hollows were designed to the holder for infrared camera capturing images during heating.

Ex vivo bronchi navigations. The *ex vivo* bronchi were pealed from commercially obtained pig lungs. The larynx, main trachea, and bronchi were preserved, and parts such as the alveoli and esophagus were discarded. After conducting bronchi navigations, the ex vivo bronchi system was further dissected for the airway foreign body removal demonstration.

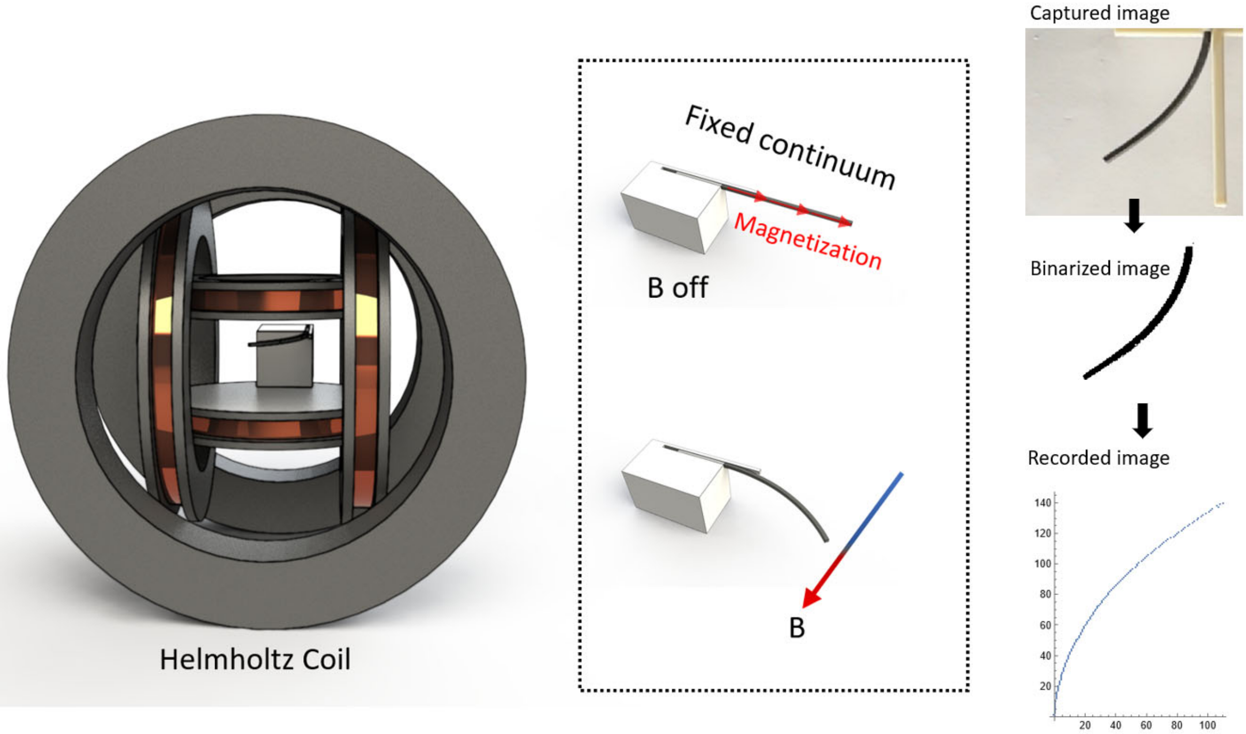


**Fig. S39. Length-width ratio effect on bending tests under Helmholtz coil control.**

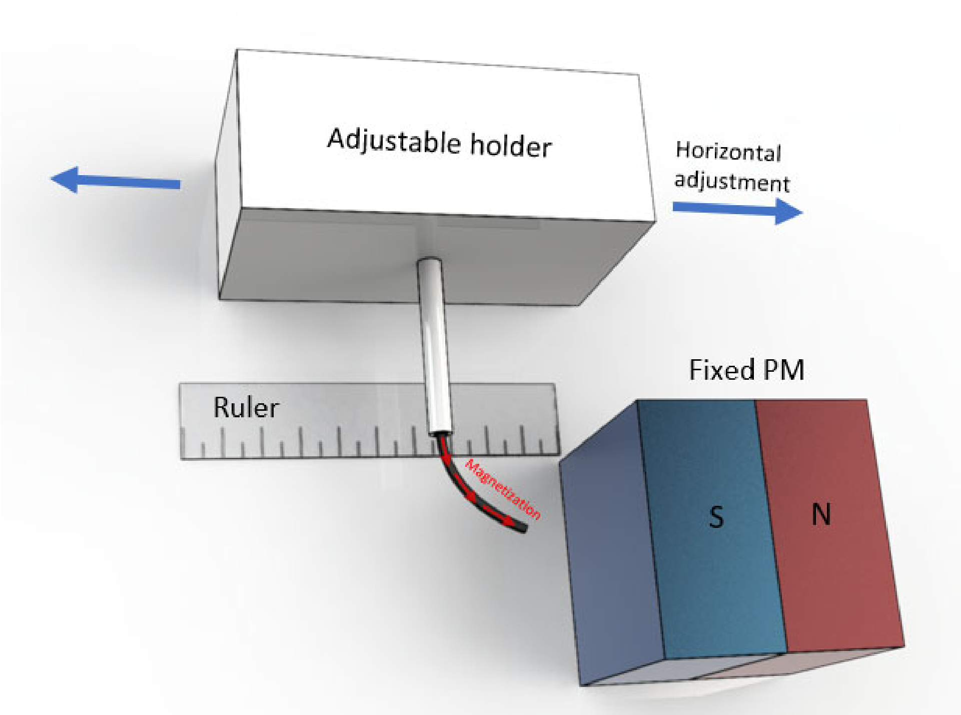


**Fig. S40. Graded stiffness effect on bending tests under EPM fields.**

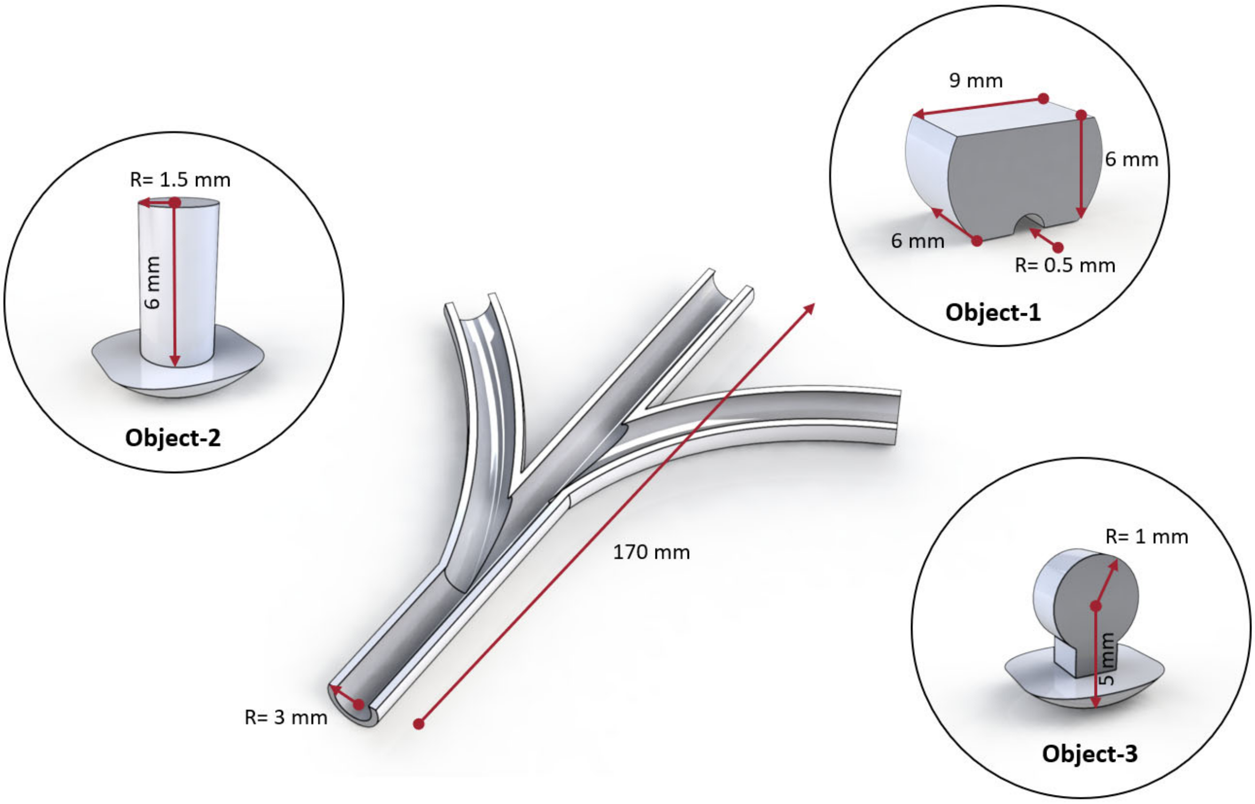


**Fig. S41. Experimental setup for “continuums-carriers” demonstration.**

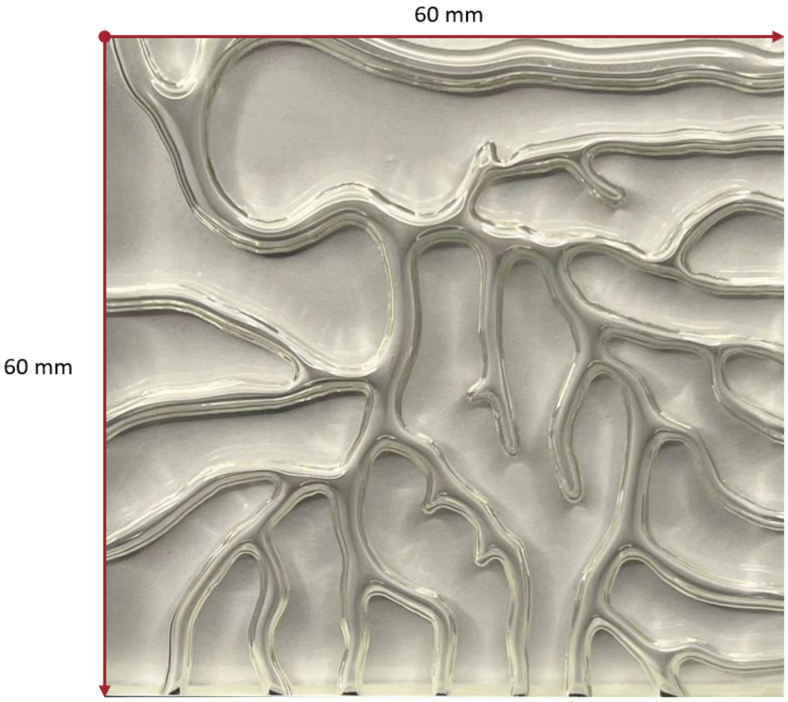


**Fig. S42. Experimental setup for complex aerodigestive tract navigations.**

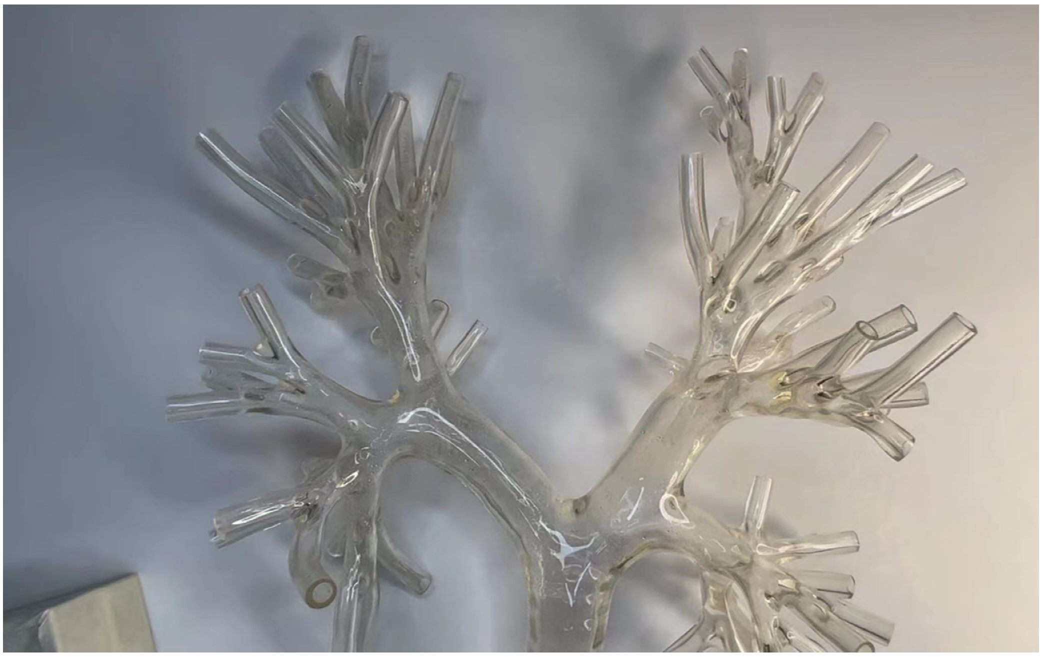

**Fig. S43. Bronchi phantom applied for navigation tests.**

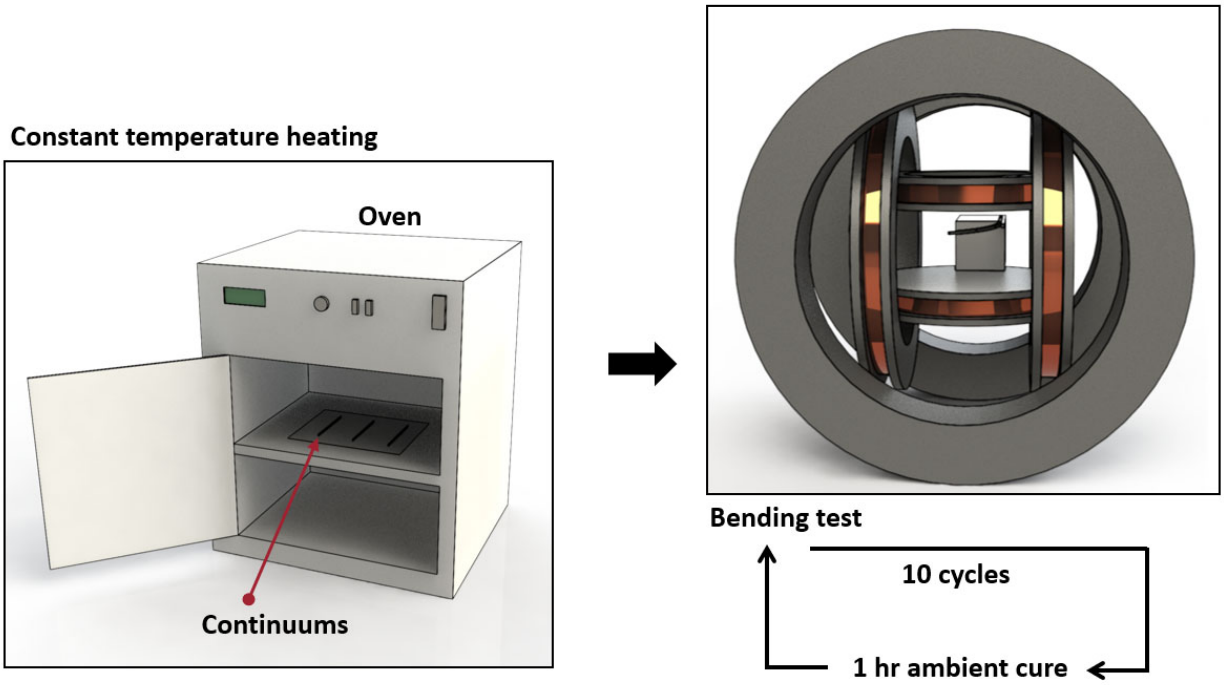


**Fig. S44. Experimental setup for thermal effects on bending performances.**

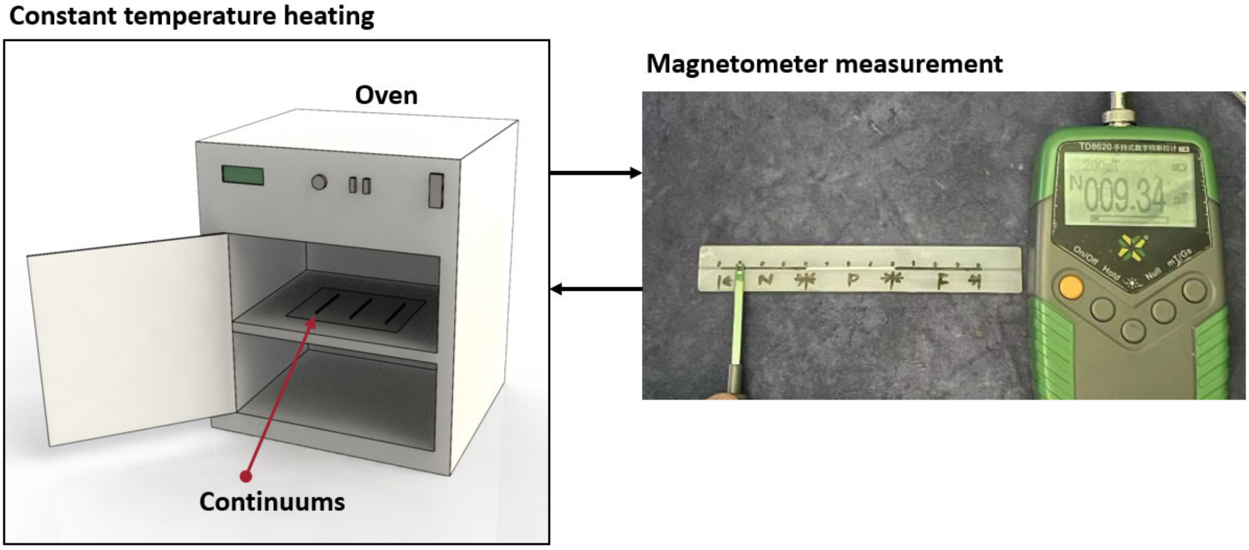


**Fig. S45. Experimental setup of demagnetization tests.**

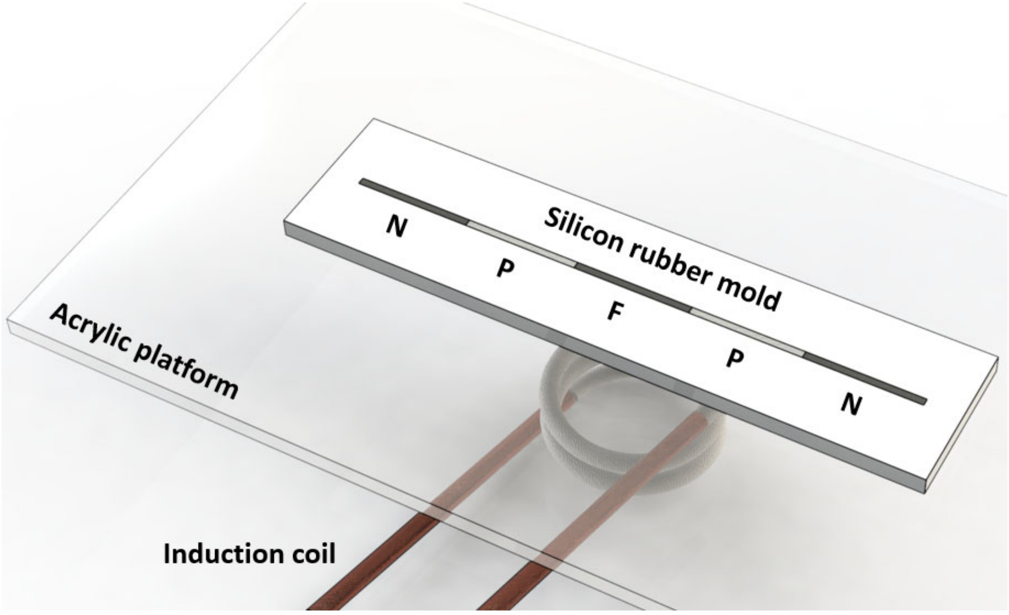


**Fig. S46. Experimental setup of local heating test.**

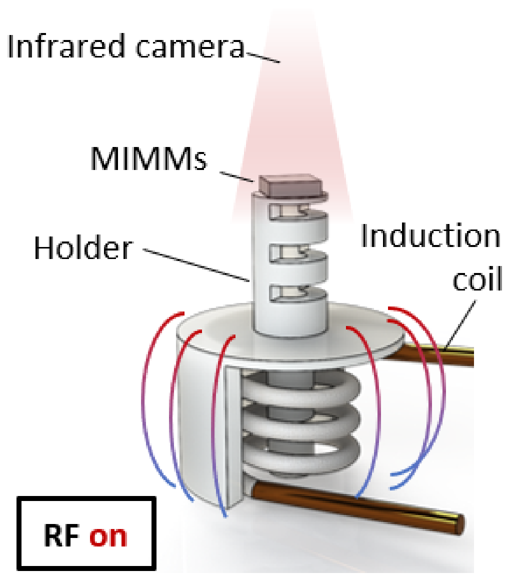


**Fig. S47. Experimental setup of thermodynamic experiment on induction heatings.**

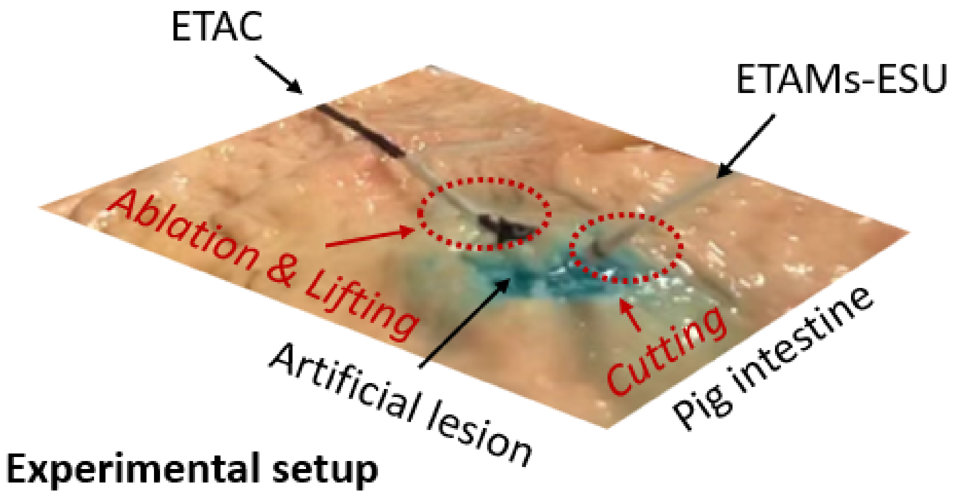


**Fig. S48. Ex vivo intestine was utilized to conduct the ESD demonstration.**

**Section 8. ETACs with/without optic fibers**

Here we discuss the consideration on whether installing the optic fibers into the ETACs. Similar to other submillimeter-scale soft continuums, ETACs can also install optical fibers for further application uses (Fig. S49). An ETAC may face difficulty performing self-division when installed with an optic fiber. As a solution, we propose the branch system division demo (Supplementary Movie- S8) to address the concern. An ETAM branch system contains a main stem and other attached branches. The optical fiber can be installed in the main stem to guarantee endoscopy purposes, and other branches can perform regrafting for various purposes, such as grasping. We successfully applied an ETAM branch system to conduct in vivo object grasping and releasing within a complex terrain to showcase its application value and controllability (Supplementary Movie- S9).

An *ex vivo* porcine lung was further applied to test the navigation ability of the endoscope-equipped ETACs. Results showed that the ETACs successfully navigate different bronchi levels, and captured images are shown in Figs. S50.

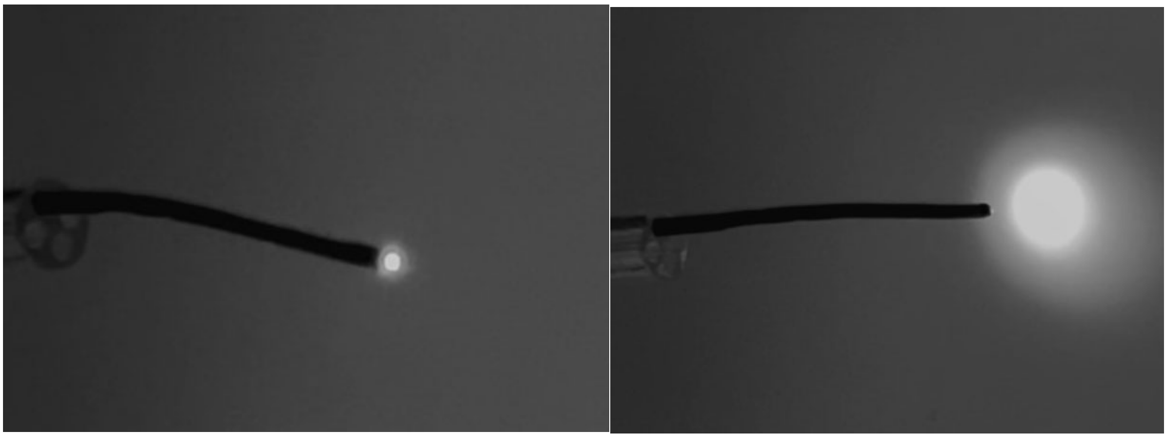

**Fig. S49. Bending performances of an optic-fiber equipped ETAC (Continuum diameter: 0.8 mm).**

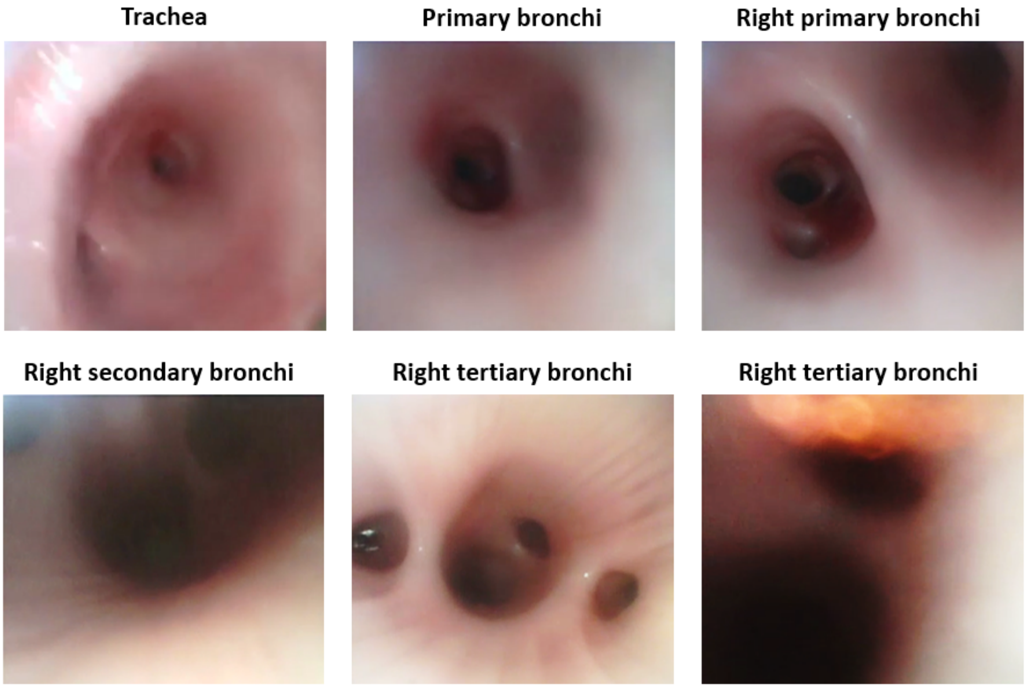


**Fig. S50. Endoscope-equipped ETAC captured bronchi images.**

**Section 9. Detailed results of complex aerodigestive tract navigations**

Fig. S51 lists the navigation process in a complex aerodigestive tract phantom.

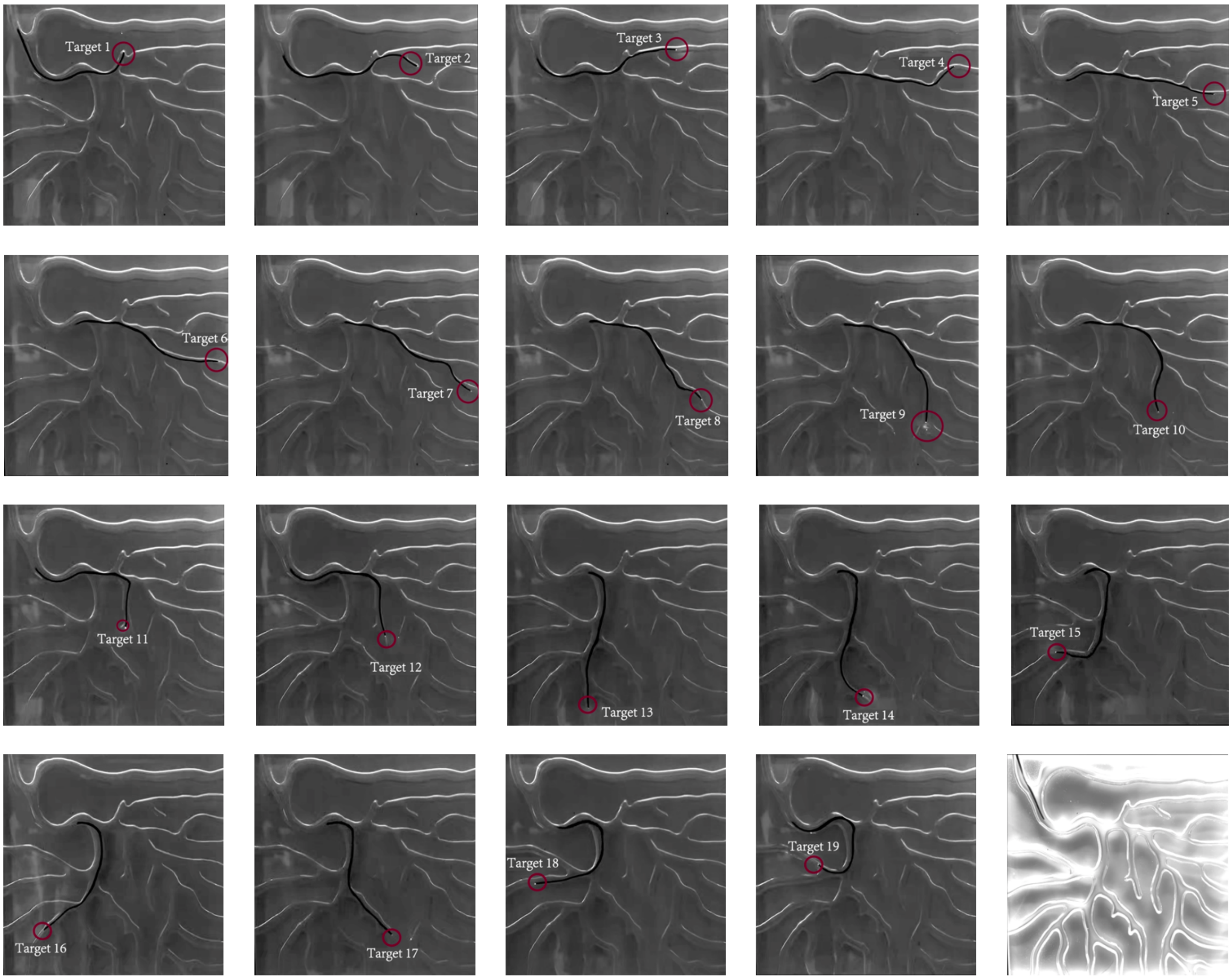


**Fig. S51. Navigation results of complex aerodigestive tract practices.**

**Section 10. Heat safety**

Heat generation is involved during regrafting in vivo, where tissue damage should be strictly forbidden. Here, we evaluate heat safety by comparing ETAMs with the heat requirements of existing biomedical devices and procedures.

Regarding heat generation in vivo, the existing literature has provided safety boundaries on temperature ranges (as shown in Fig. S52). Divided by the epidermal injury temperature curve and complete irreversible damage temperature curve, the heating temperature-time area contains the body temperature range, moderate ablation range, and ideal therapeutic ablation range. Body temperature range refers to the commonly acceptable working temperatures of biomedical devices. Moderate ablation range is applied for liver, pleural, and abdominal tumor radiofrequency ablations (RFAs). The ideal therapeutic ablation range was proven to be a satisfactory operation range between immediate coagulation and tissue vaporization/carbonization, which has a relatively high surgery efficiency.

As explained in the Discussion section, there are three terms regarding the temperature: preheating temperature (i.e., 60 °C for 10 mins in the oven and cool down to ambient while maintaining the reduced stiffness), softening temperature (i.e., 45-50 °C for ~10 s with/without tissue contact), and ablation temperature (i.e., 120 °C for 10-30 s). We note that the regrafting is located at the body temperature range, which is safe for *in vivo* applications, and the ablation temperature can be adjusted within a temperature range of 55-120 °C. We note that the provided demonstration “continuums carriers (Fig. 5)”, “ETAM end-effector (Fig. 6)”, and “submucosal dissection (Figs. 7-8)” respectively showcase the safe tissue contact (45-50 °C for ~10 s contact is regarded safe according to Fig. S52), tissue contactless regrafting (regrafting point locates at the air due to the geometry design of ETAM end-effector) and ablation.

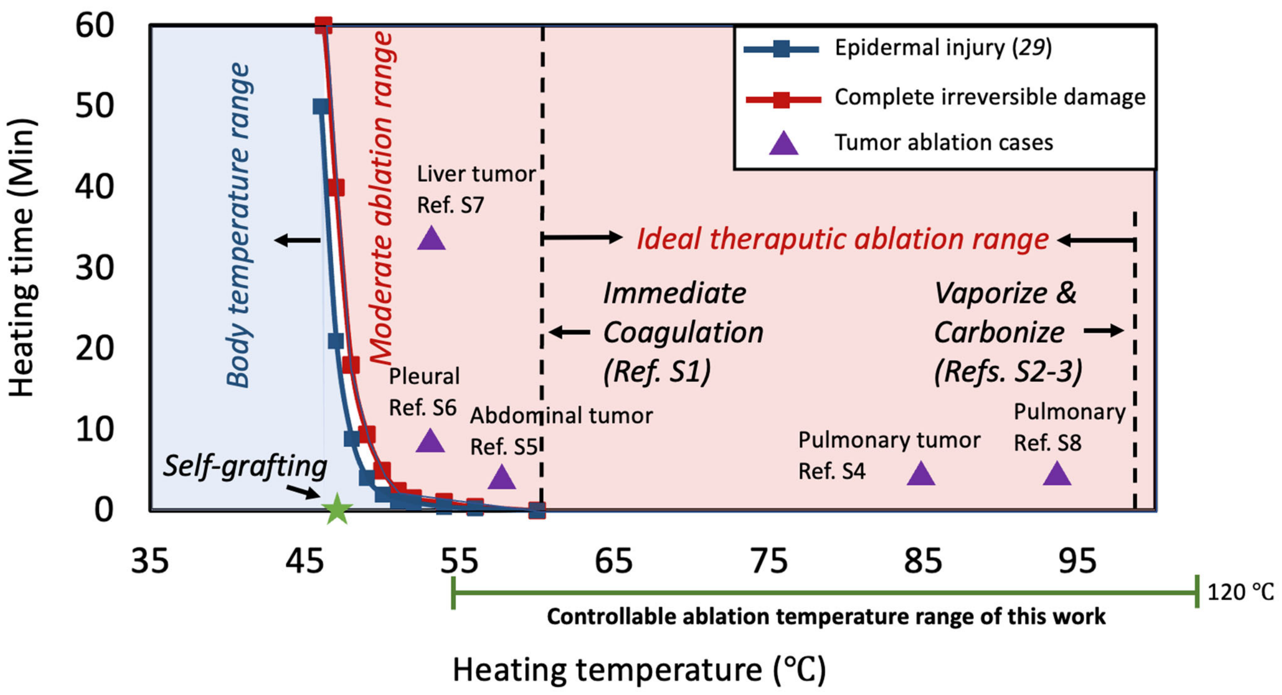


**Fig. S52. Heating temperature-time relation for biomedical uses. Regrafting is proven to be safe for *in vivo* uses.**

## Section 11. Motivations and creativities

### [Regrafting]

**Table. S3. Comparisons between regraftable FSCs and existing well-accepted biomedical devices.**

| Demonstration | Device Type | Necessary Requirements of Device | | | | Surgical Scenario | Benefits |
|---|---|---|---|---|---|---|---|
| | | Size < 1 mm (OD) | Collaboration | >200 dgrs bending with a curvature radius of 2 mm | Complete in one go? | | |
| Demo 1: Continuum carriers | Tendon-driven continuums (*S9-S12*) | × | √ | × | × | Bile duct stones removal<br>Nasolacrimal duct<br>Bladder<br>Bronchi<br>(Graft outside the fluid-laden passages) | ETACs' challenging local bending and self-graftability can complete tasks in one go which greatly enhances the surgical efficiency |
| | Fluid-driven Continuums (*S13-S15*) | × | √ | × | × | | |
| | FSCs (*1, S16-S17*) | √ | × | × | × | | |
| | Self-graftable FSCs (ETACs) | √ | √ | √ | √ | | |
| Demo 2: Airway foreign body removal | | Shape adaptability | Without disturbances | Multiple removal strategy | Stiffness tunability | Respiratory system | Low object disturbances and multiple removal strategy (grasp or stick) make ETACs more suitable for foreign body removal |
| | Retrieval baskets (*S18-S20*) | √ | × | × | √ | | |
| | Tendon-driven grasper (*S21-S23*) | × | × | × | √ | | |
| | Self-graftable FSCs (ETACs) | √ | √ | √ | √ | | |
| Demo 3: Submucosal dissection | | Grasper free | Steerable ESU | Shape adaptable RFA | Units collaboration | Gastrointestinal tract | ETACs can replace grasper and ablation function of ESU, which greatly enhance the surgery efficiency |
| | Tendon-driven continuums (*S24-S26*) | × | √ | × | √ | | |
| | Self-graftable FSCs (ETACs) | √ | √ | √ | √ | | |

In this section, we explain the motivations for investigating regraftable ETACs and highlighting their creativities by comparing them with existing well-accepted biomedical devices.

As described in the Introduction section of the main text, ETACs are designed to endow submillimeter-scaled ferromagnetic soft continuums with more complicated functions for in vivo manipulation and therapy. There are two goals for developing regraftable FSCs: one is to complete the task that other devices are difficult to realize (demo 1: continuum carriers), and the other is to improve and optimize the procedure with enhanced operating efficiency (demo 2 & 3).

To showcase the motivations and creativities of regraftable FSCs (ETACs), we carefully designed three demonstrations and compared them with existing well-accepted biomedical devices, as shown in Table S3. Regarding demo 1, given the challenging steerability (>200° with a curvature radius of 2 mm), ETACs can grab multiple objects out of confined spaces in one go, where regrafting plays an irreplaceable role, significantly increasing the surgery efficiency. Demos 2 and 3 showcase that, with regrafting, the performances of existing devices gain obvious enhancement, improving the surgery success rate, accuracy, and efficiency.

Beyond engineering breakthroughs in biomedical robotics, this work provides soft robotics with fundamental contributions from a scientific perspective: developing a phase change ferromagnetic smart material to actively switch between

two opposing characteristics: sufficient strength (e.g., ~MPa) for external loads and low fracture limit (e.g., ~kPa) for division and mergence. ETAMs provide a satisfactory answer to the fundamental question, thereby being fabricated to enable ETACs to perform regrafting. With interdisciplinary studies, regrafting mechanisms are explained in detail and are ready to provide engineers with ready-to-use knowledge for further possible engineering applications.

**[Self-division]**

Self-division is considered an essential ability of ETACs due to its contribution to robotic switchability, shape reconfigurability, and function reinforcement. Here, we explain these three significant meanings from the fundamental scientific and engineering perspectives (as shown in Fig. S53).

From the fundamental scientific perspective, self-division plays an irreplaceable role in formulating the regrafting mechanism, which contains robotic switchability, shape reconfigurability, and function reinforcement.

- Robotic switchability refers to the ability to actively generate untethered robots from a tethered robot body. This process can break the restrictive definition of a robot that should be either tethered or untethered, thereby enhancing flexibility from the robot design level, where self-division is the necessary condition (*Ref. S27: a combination between tethered and untethered robots for biomedical purposes*).
- Shape reconfigurability refers to the ability to transform robot shapes from original to new forms. Submillimeter-scale ferromagnetic continuums are designed to work in narrow and confined spaces, where *in situ* shape reconfiguration will significantly enhance robots' functionality and flexibility (*Ref. S28: the shape-changing robot traverses environments better than an equivalent but non-morphing robot, in simulation and reality*).
- Beyond shape reconfigurability, self-division endows the reconfigured robots with extra functions, i.e., function reinforcement ability. Taking the ETAM end-effector as an example, without self-division, it can only perform "open" and "grasp" motions but cannot realize the "lock" and "release" which are substantial functions to tightly grasp objects for removal. With function reinforcement of self-division, the functionality and flexibility of robots will be further enhanced (Regrafting/division reveals a fundamental concept like *in situ* 4D printing process, *Ref. 29*).

From the engineering perspective, self-division is also essential in implementing regrafting for surgical applications. We explain the necessity by two examples:

- In demo 1 (Fig. 5, continuums carriers), self-division is the precondition of realizing parallel operations *in vivo*. The parallel operation has the potential to be commonly applied in emergent surgical cases, such as heat coagulation (*multiple RFA zones for heat coagulations in Ref. 30*), multiple duct stone removal (*Ref. S31 shows that it is desired to remove multiple stones in one go*), and other first-aid in confined lumens. Given the emergence and complex *in vivo* passages, completing multiple surgical tasks (such as heat coagulation for multiple bleeding points or removing multiple embolisms) in one go is preferred for time-saving and therapeutic efficiency, which is the goal of parallel operation. It naturally indicates a device that can conduct *in situ* division. Therefore, we regard self-division as a necessary precondition for conducting parallel operations.
- In demo 2 (Fig. 6, foreign body removal), self-division is necessary to guarantee the end-effector's reusability. Without self-division, once the tips are merged, the end-effector's geometry will remain fixed. Given the need to grasp multiple objects in one go (*Ref.32, multiple foreign bodies removal*), the end-effector will be difficult to complete without self-division. Therefore, we regard self-division as a necessary condition to perform reusable functions.

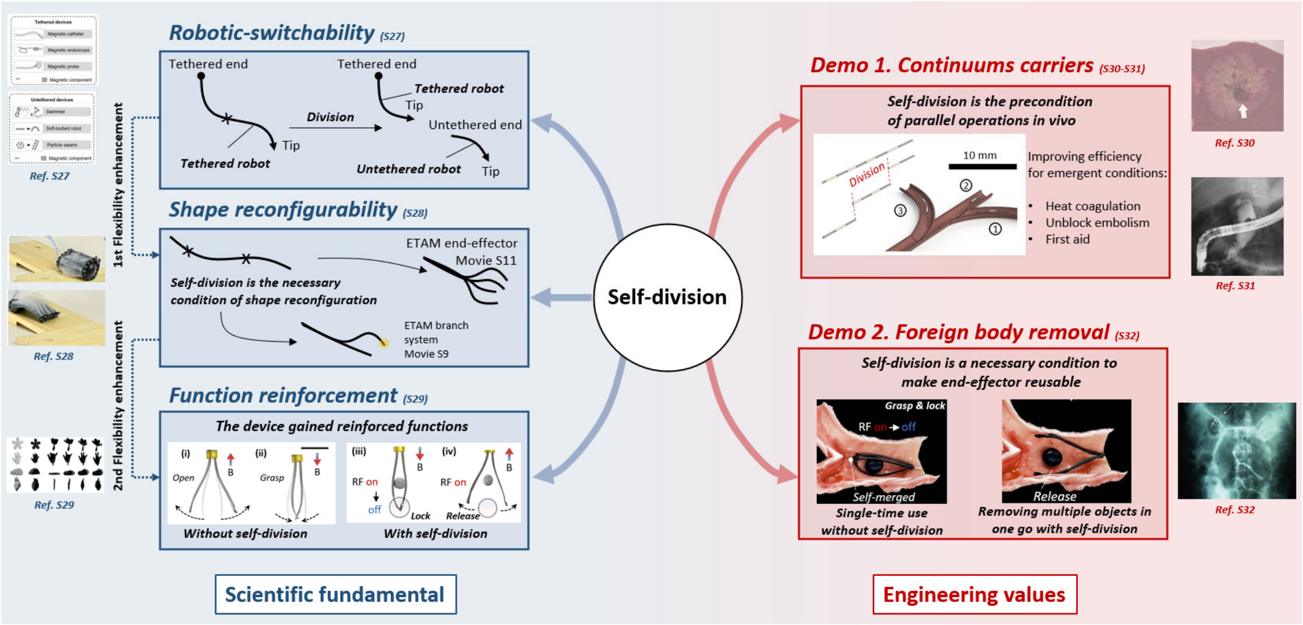


**Fig. S53. Necessity of self-division from fundamental scientific and engineering perspectives (*S27-S32*).**

**Section 12. Animal trial**

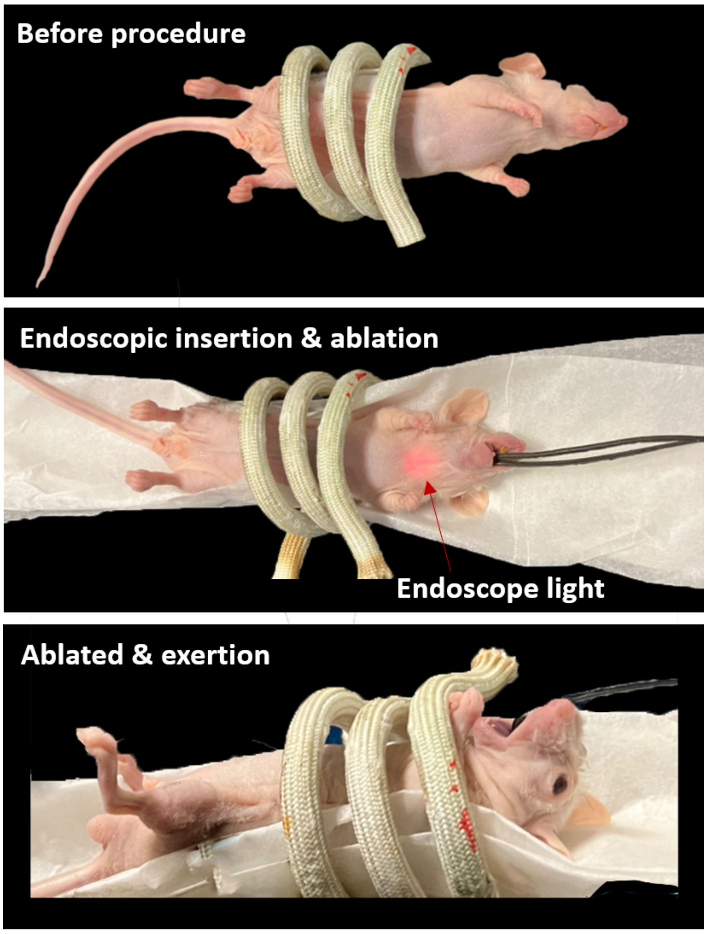


**Fig. S54.** ***In vivo*** **ablation on a hairless mouse.**

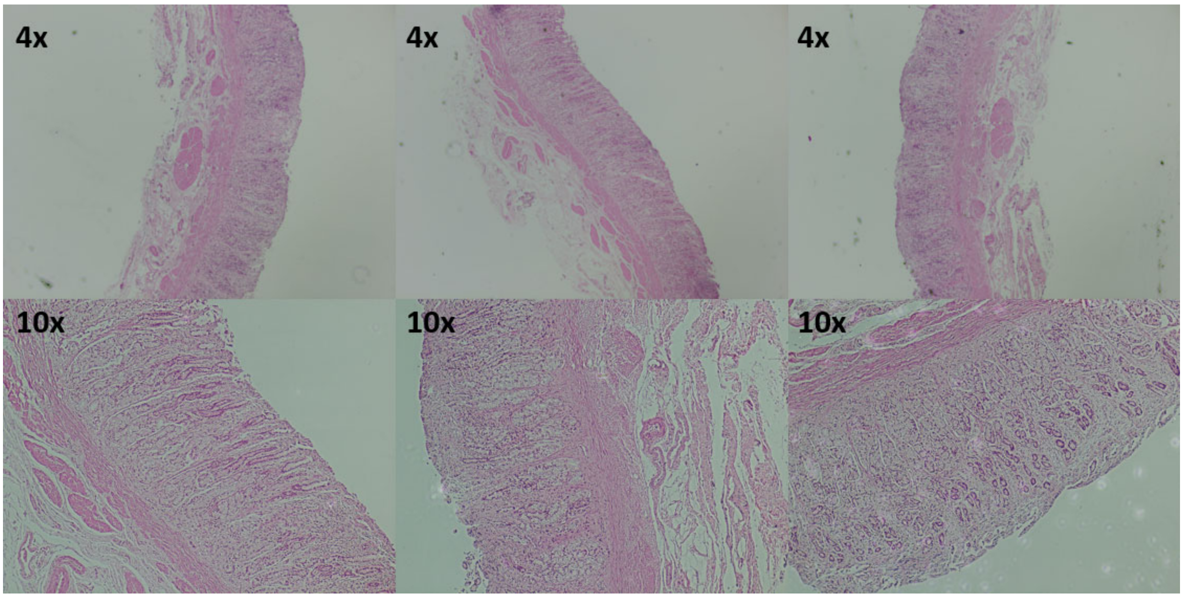


**Fig. S55.** ***Ex vivo*** **porcine stomach tissues- control group.**

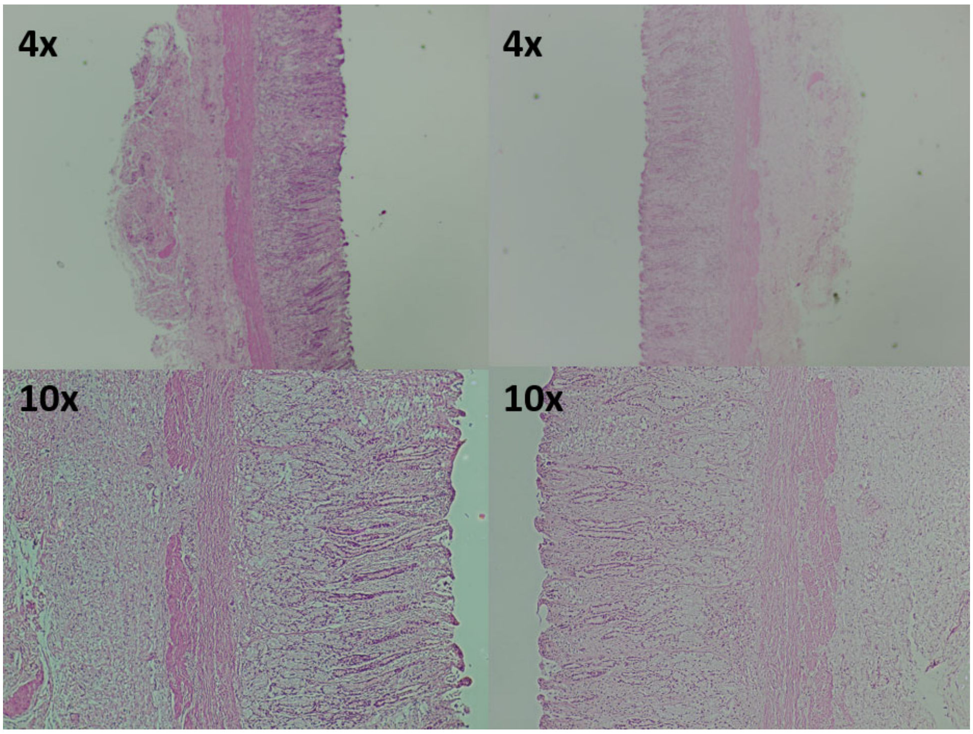


**Fig. S56. Ablated *ex vivo* porcine stomach tissues- 10 s-ablation groups.**

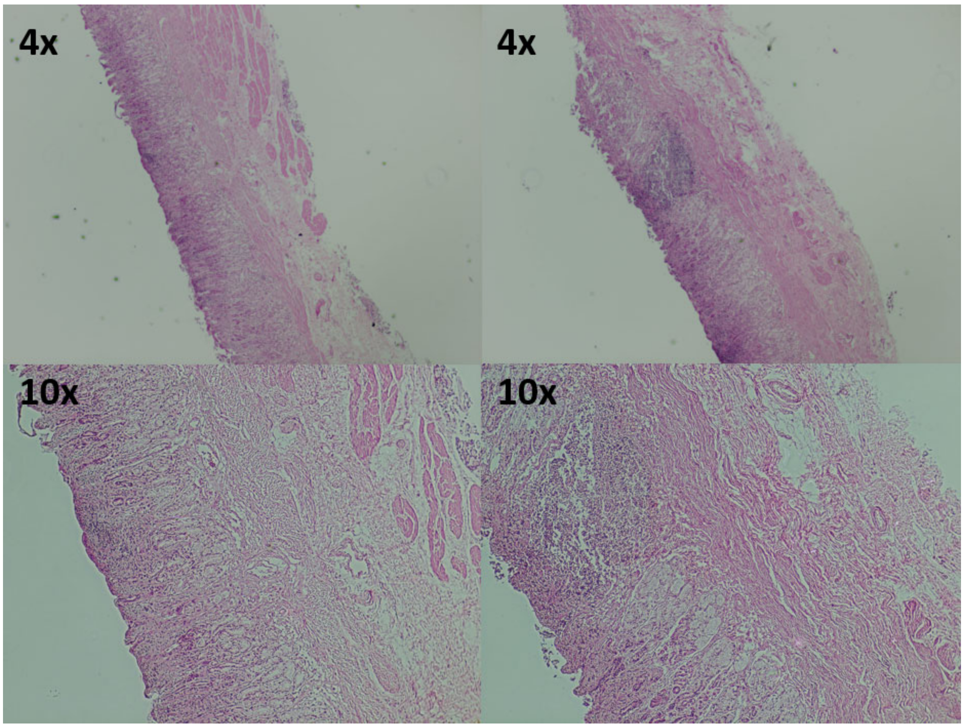


**Fig. S57. Ablated *ex vivo* porcine stomach tissues- 30 s-ablation groups.**

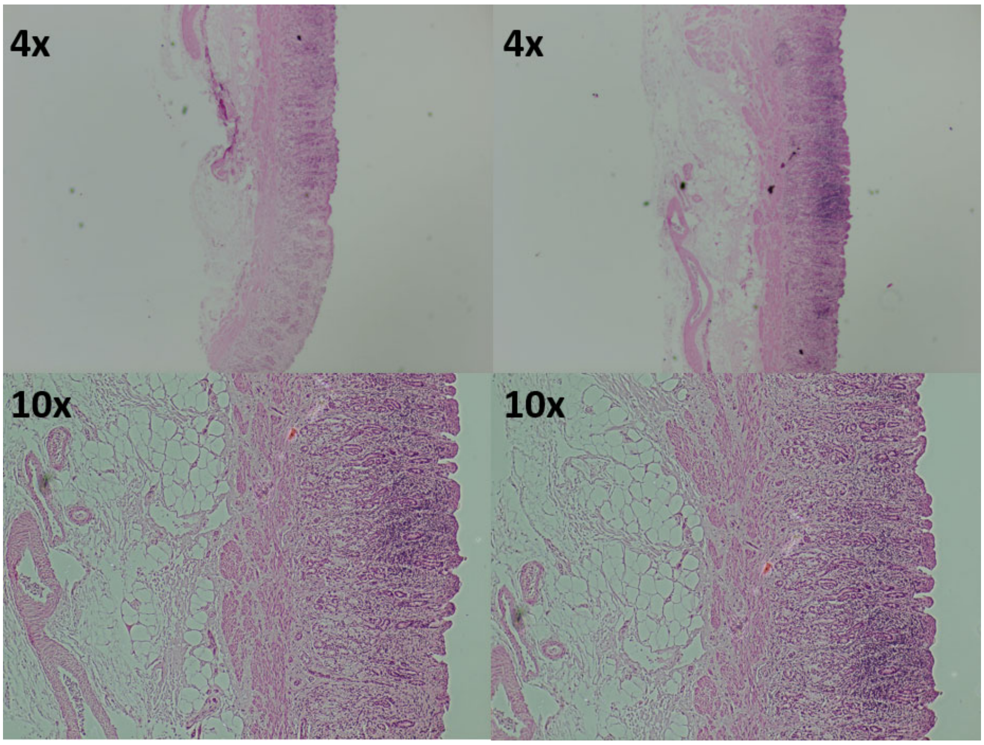


**Fig. S58. Ablated *ex vivo* porcine stomach tissues- 60 s-ablation groups.**

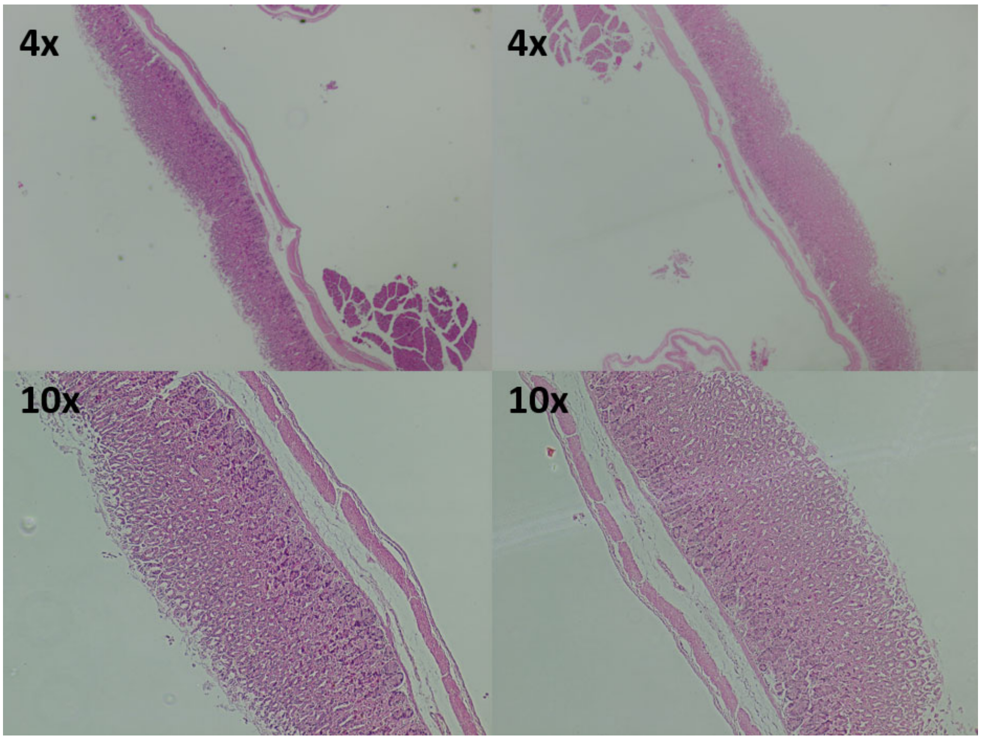


**Fig. S59. Mouse stomach tissues- control groups.**

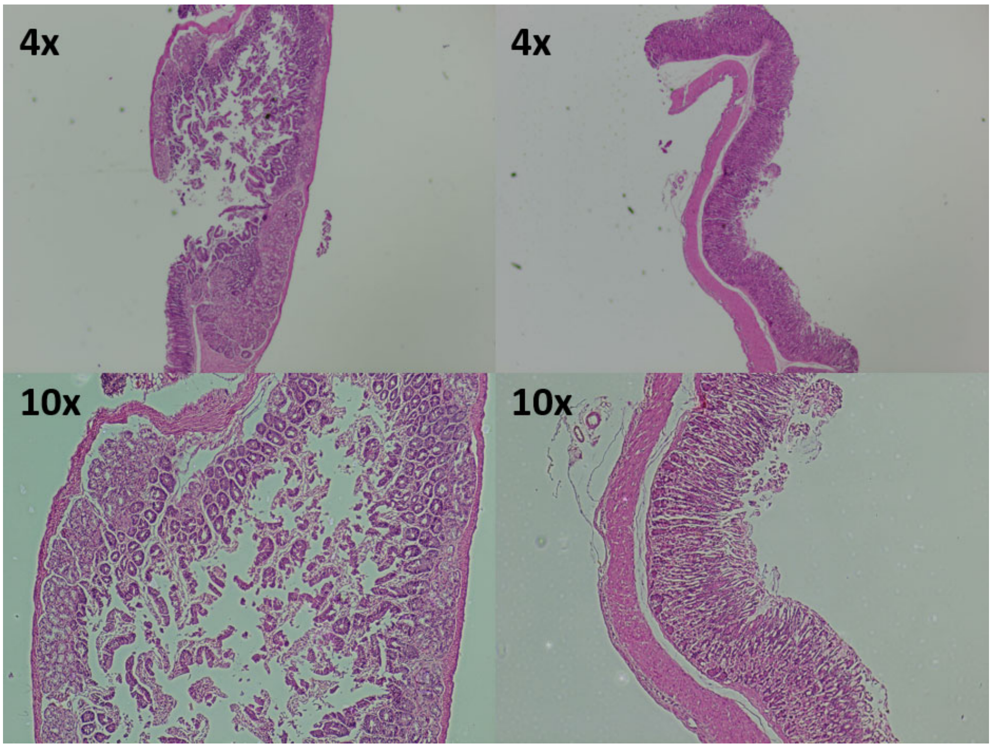


**Fig. S60. Ablated *in vivo* mouse stomach tissues.**

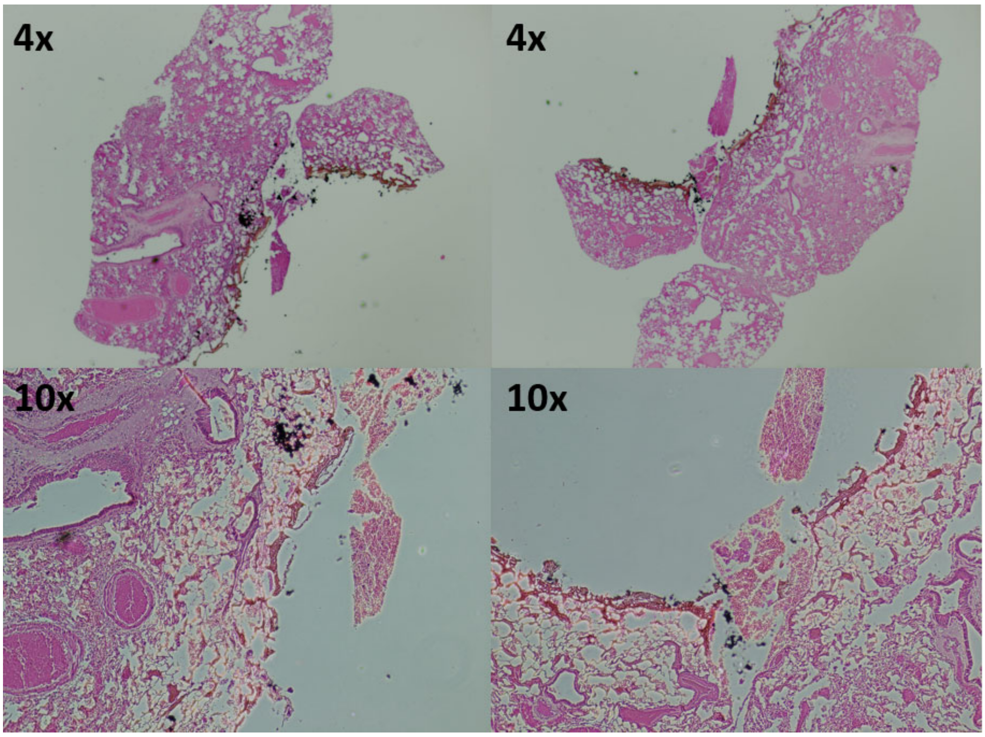


**Fig. S61. Ablated *in vivo* mouse cardia (lower esophagus) tissues.**

**Section 13. In situ grasper assembling**

Due to the flexibility and regrafting ability, an ETAC can be in situ assembled through five steps. An ETAC was first inserted into a trachea and magnetically guided to the target region (Figure S62 B-i). By applying a magnetic field in the opposite direction of ETAC's Preprogrammed magnetization profile in the axial direction, the ETAC was folded to form a circle shape at the tip region (Figure S62 B-ii). After aligning, an RF field was applied at the tip region to conduct self-mergence (Figures S62 B-iii&iv). Then, the tip of the circle region was heated to conduct self-division, forming a two-arm grasper (Figures S62 B-v). The assembled grasper can perform the same functions as demonstrated previously (Figure S62 C).

Regarding the advantages over other existing bronchoscopic forceps, as we stated in the main text, there is a risk of unsuccessful object grasping with existing devices for foreign body removal due to their grasping principle [R33-R35]. Two primary methods are commonly employed to accomplish tasks, one is using retrieval baskets which constrain objects by applying mechanical forces. The retrieval baskets are utilized to remove large objects with irregular geometries, however, during this process, there is a possibility of the object escaping due to the counterforce exerted on the basket, which may lead to further issues such as relocation or even severe channel blocking [R33]. Moreover, inserting retrieval baskets into confined body lumens can result in side effects such as mucosal injury. The other approach is using endoscopic graspers which have the limitation to adapt to oversized or fragile object such as nuts [R34-R35]. In this work, we provide an alternative approach to complete airway foreign body removal with minimal object disturbances. The proposed ETAM-grasper has the potential to grasp irregular large geometries while  preventing damage to fragile objects.

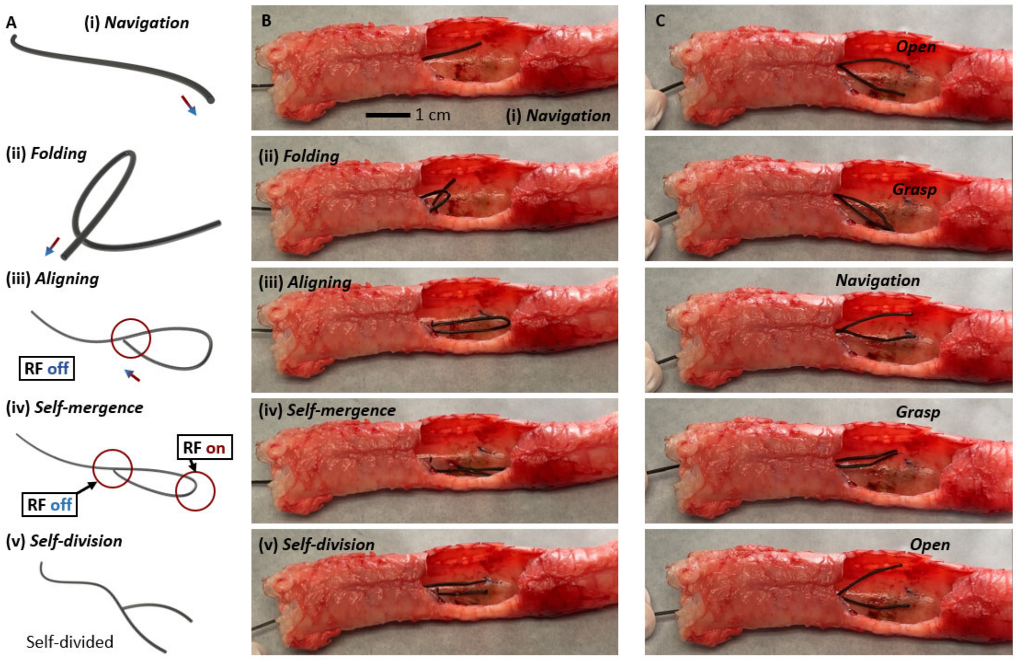


**Fig. S62.** In situ assembly. **(A)** There are five steps to assemble an ETAC into a grasper, including navigation to the target, magnetic folding, aligning, self-mergence, and self-division. **(B)** Real-time figures showing the assembling process in an ex vivo pig trachea. **(C)** The in situ assembled grasper can conduct the same functions as shown in the previous demonstration, such as navigation, grasping, and releasing.

**Section 14. Movement precision of ETACs**

Regarding the movement precision of the ETAM aiding the ESU, we have conducted a series of movement precision tests. In the continuum control area, there are two main test methods to determine the moving precision of a continuum robot: movement resolution and position repeatability. As shown in Figure S63 A, we installed an ETAM-ESU in a Helmholtz coil where a grid mesh was placed beneath the ESU for motion measurement. The magnetization profile of the ETAM-ESU was preprogrammed along the axial direction and the magnetic field direction was perpendicular (defined as y-direction) to the magnetization profile for bending actuation. The movement resolution results are shown in Figure S63 B-C. The ETAM-ESU performs satisfactory linear controllability with the increment of actuation voltage (namely magnetic moment). As shown in Figure S63 D, the position repeatability showed ~5% averaged positioning error, and it was observed that the position repeatability is higher in the low magnetic moment range than in the high moment range. This is because the positioning error accumulates with the increment of the magnetic moment.

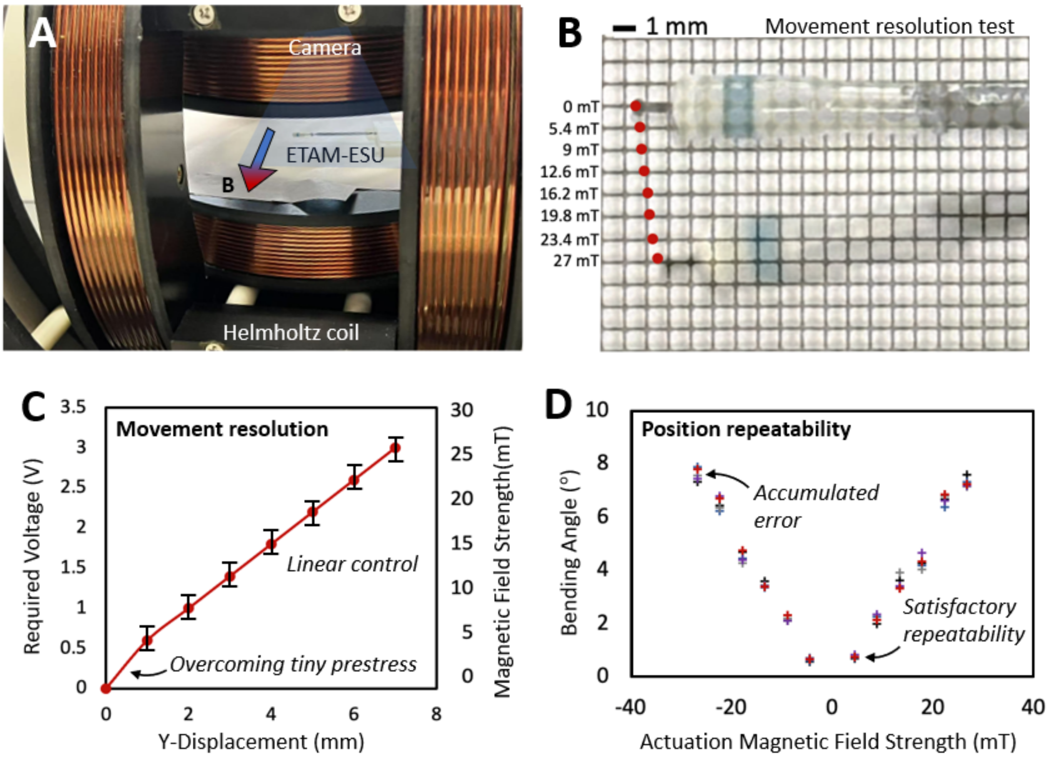


**Figure S63. Movement precision test of ETAM-ESUs**. (**A**) Experimental setup of movement precision. (**B**) Real-time image of the movement resolution test results. (**C**) Movement resolution results showing a linear control relation between actuation required voltages and Y-direction displacement. (**D**) Position repeatability results showing a relation between the actuation magnetic field strength and bending performances.

**Section 15. Temperature monitoring**

The device temperature changes with multiple parameters, such as heating time, the RF coil distance to the device, and the environmental conditions (mucous in lumens). Considering all these factors, we have designed an experiment for temperature monitoring. As shown in Figure S64 A, we installed a thickness-adjustable holder on an RF coil and an ETAM was placed at the middle of the holder. An infrared camera was utilized to record the temperature changes at the top of the holder (Figure S64 B). The temperature was collected and plotted as shown in Figure S64 C. When the heating distance is close to the ETAM (less than 1 cm which is impractical for practical application), the temperature significantly increases in less than 1 minute. In such cases, although the relation between heating time and temperature is linear, the over-sensitive temperature changes will be difficult to control for operational safety. With an increment in heating distance, the heating speed tends to slow down and become more controllable (such as at 12-16 cm heating distance). Nonlinear curve fitting can be adopted to establish an empirical formular to predict the temperature changing. Moreover, lubrication from mucous was observed to slow down the heating speed due to heat dissipation, which can also be considered as an influence parameter to be included in the empirical formula.

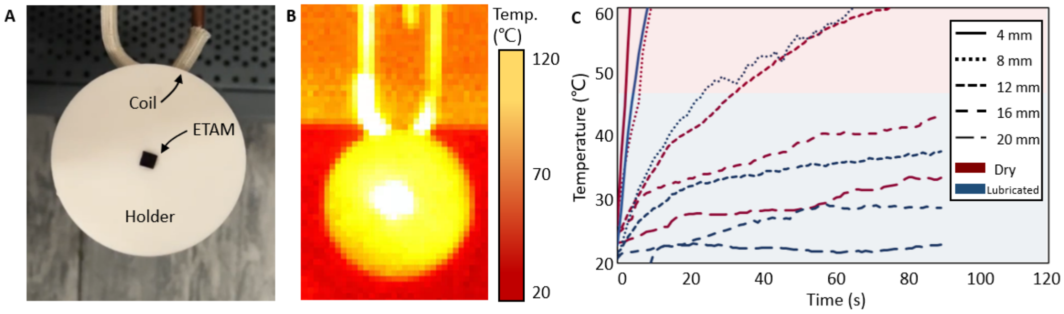


**Figure S64.** Empirical temperature monitoring method. (**A**) Experimental setup for temperature monitoring test. (**B**) Obtained infrared image for data collection. (**C**) Experimental results showing relations between heating time, device temperature, heating distance, and lubrication status.

We understand the heating distance may still not be practical for some specific scenarios, such as in the digestive and respiratory systems. We regard this issue as an industrial problem that will be effectively addressed by increasing the size and power of the RF machine [36].

Here, we provide our rationale regarding the concerns on heating efficiency and resulting field safety in detail. Current laboratory RF equipment is limited by its size and power which are much lower than existing industrial and medical devices [36-38]. For example, the RF frequency of existing medical devices ranges from 128 MHz (3T) to 342 MHz (8T) which are more than 400 times higher than our laboratory RF machine [37]. While providing satisfactory RF efficiency, such industrial RF machines can support relatively long-distance heating. The maximum coil diameter we found is 56 cm, which is acceptable for practical application [37-38]. Regarding the field safety consideration, there exists animal safety evaluation research for extreme RF exposure. Researchers found that 10–12 T RF exposure for a continuous 28 days was relatively safe for mice. It was also reported that 3.5–23.0 T RF exposure for 2 h and 7.0–33.0 T for 1 h did not have severe long-term detrimental effects on mice [39-40]. Therefore, we regard the heating efficiency and field safety as well-proven issues which can support our proposed regrafting technique.

**Section 16. Lubrication needs**

Here, we address three concerns: 1) Could lubrication enhance the performance of uniform modulus ETACs? 2) Will the regrafting function be affected by lubrication? 3) How do the natural fluids within bodily conduits affect regrafting function?

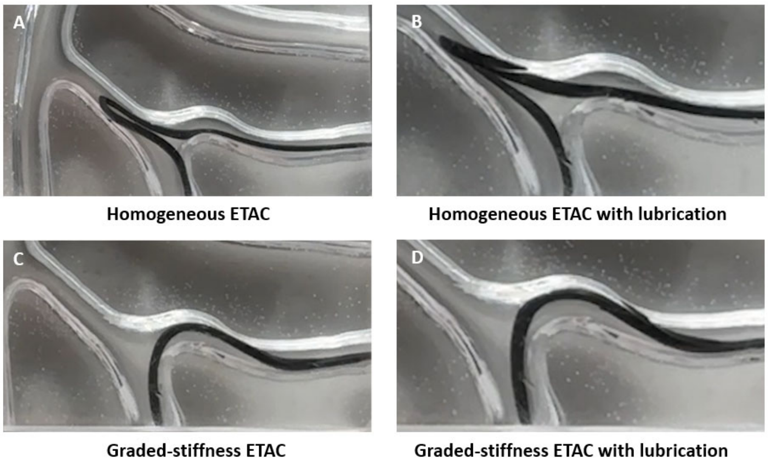


**Figure S65.** Comparison test between uniform modulus ETAC, uniform modulus ETAC with lubrication, graded-stiffness ETAC, and graded-stiffness ETAC with lubrication.

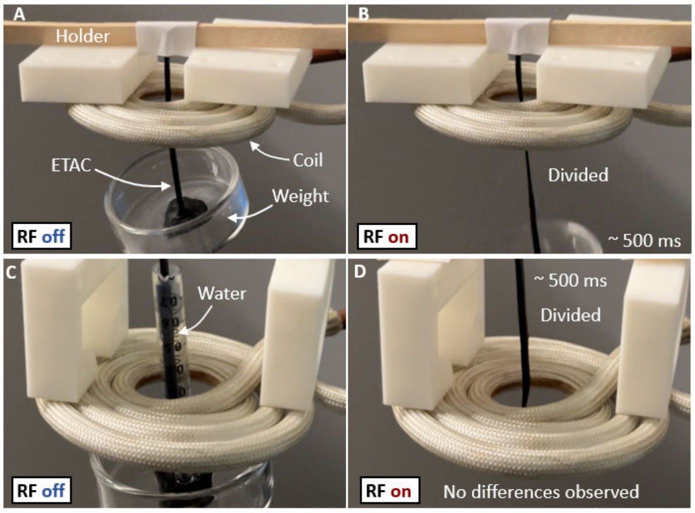


**Figure S66.** Qualitative comparison tests on the heat dissipation effect of lubrication. (**A**) Experimental setup of the non-lubrication test. (**B**) Self-division of the non-lubrication test. (**C**) Experimental setup of the lubricated test. (**D**) Self-division of the lubricated test.

Regarding the first question, we have newly conducted a comparison test between uniform modulus ETAC, uniform modulus ETAC with lubrication, graded-stiffness ETAC, and graded-stiffness ETAC with lubrication, as shown in Figure S65. To maintain ETACs' simple structural design and regrafting normal function, we applied medical device lubricants as instructed by medical doctors (No. LUB0005, Health&Beyond Hygienic Product Inc., China) instead of using lubrication coating layers. The results show that the lubrication did not improve a uniform modulus ETAC's navigation performance. The uniform modulus ETAC failed to complete a ~270° steering due to the unoptimized force transmission which is not directly related to lubrication conditions. When the graded stiffness structure is applied to ETAC's tip, no obvious differences were observed between ETACs with/without lubrication.

Regarding the second and the third questions, we have newly conducted a qualitative experiment and a quantitative test to investigate the heat dissipation effect of lubrication. As shown in Figure S66 A, we installed an ETAC inside an RF coil with a weight attached at the end of the ETAC. The ETAC completed self-division in ~500 ms after turning on the RF machine (Figure S66 B). We further installed an ETAC inside a tube filled with water for comparison (Figure S66 C). No obvious differences were observed with respect to the division shapes and time (Figure S66 D). This is because the heating power is relatively high and the heating distance is short enough to minimize the performance differences. The results indicate that improving the heating efficiency will minimize the heat dissipation effect from lubrication. Results shown in Figure S66 provide a quantitative comparison between ETACs with and without lubrication. For practical uses, heat dissipation can be systematically tested to develop a temperature monitoring empirical formula.

**Section 17. Softening and melting point**

Given ETAM's wide material modifying space, there will generate hundreds types of ETAMs with different specific values for softening and fluidification. Quantifying every type of ETAMs is time consuming, which is considered as an engineering activity. In this work, we would like to provide a quantification principle to guide engineers in future studies.

A Young's modulus and a viscosity value are respectively defined as the softening point and fluidification point. Based on test experiences, when the ETAM stiffness is reduced below 1 MPa (namely heated to the kPa level), it can be divided with a force lower than 2 N. Regarding the fluidification point, 440 Pa·s is the highest viscosity we collected where liquid ETAM can conduct flowing motions under magnetic actuation, which is defined as the fluidification point. It should be noted that different ETAMs require varied temperature to achieve the above softening and viscosity values. By recording the softening and fluidification temperatures of different ETAMs, a material property database can be established. Here, we provide preliminary results in Figure S67.

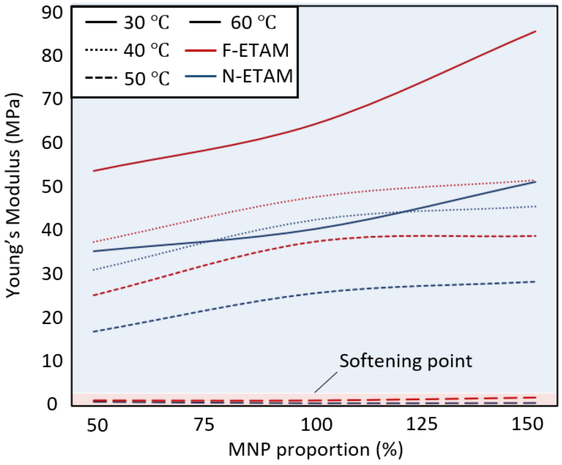


**Figure S67.** Relation between ETACs' stiffness, MNP proportions, ETAC types, and temperature.

**Section 18. Heating area issues**

We propose two methods to reduce the distance effect on the heating area: 1) customize RF coil geometry and 2) utilize a heat distribution center to conduct regrafting.

The first approach is customizing RF coil geometries to fit different conditions. The width and depth of generated RF fields strongly depend on the geometries of RF coils. As shown in Figures S68 A&B, for example, the solenoid coil generates a long depth and small width RF field for long-distance heating scenarios. However, disk coils generate short depth and large width fields, which fit for wider area heating when there are multiple regions to be heated. We understand that only adjusting coil geometries may still face division precision issues. Therefore, adopting our proposed second method will further reduce the heating area effect and improve the division precision.

The second method is utilizing the heat distribution center to conduct regrafting. Given the RF field's gradient property, every RF field has a central region providing the strongest heating effect. We regard this region as the heating center (Figure S68 C). It is true that the RF field becomes more diffuse as the distance from the coil increases. However, the heating center still exists and its position can be adjusted by moving coils. After a few seconds of cooling, the heating center remains in the softened state while other heated regions are at a lower temperature. At this time, the heating center is the place to conduct regrafting, including division and mergence.

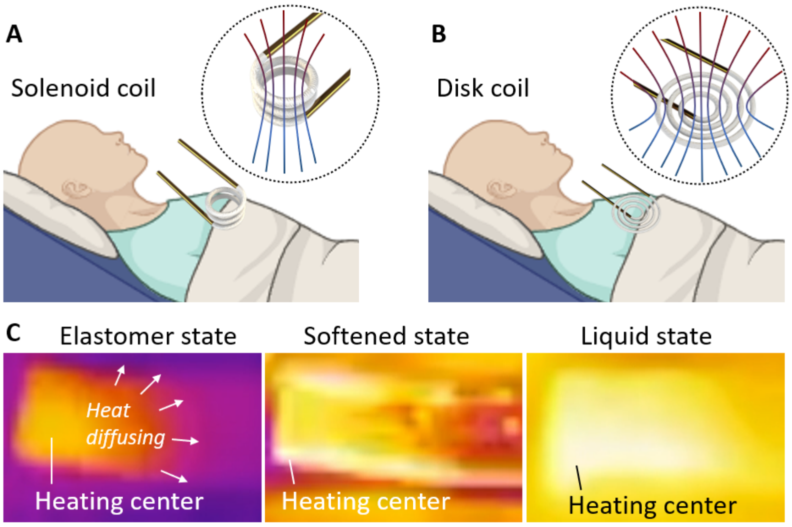


**Figure S68.** Two methods to reduce heating area: customizing RF coil geometry and utilizing heat distribution center. (**A**) The RF field generated by a solenoid coil. (**B**) The RF field generated by a disk coil. (**C**) Infrared images showing the heating center's existence. Created in BioRender. Yang, Y. (2025) https://BioRender.com/7idnddp

**Section 19. Magnetization change issue**

Magnetic particles have the chance to rotate under an external magnetic field rather than transmitting torques when the PCL matrix is significantly softened. However, such a phenomenon was not obviously observed in our case due to the following reasons:

1) The magnetization re-arrangement phenomenon strongly depends on the softening condition of the PCL matrix. It is known that there is a temperature range greater than 40 degrees Celsius for the softening state, which is a transitional state between the elastomer state and the fluid state. The temperature we chose to conduct actuation is located at the lower region of the softening state temperature range. Under such conditions, the PCL matrix has a relatively satisfactory ability to transmit torque.
2) In Figure 2B, the tiny errors between heating and after curing the ETACs are mainly due to the stiffness differences, which are also well-proven by numerical simulations. The cure time we selected is 10 hrs for 86% ~100% stiffness recovery rate. Given our experiences, within 24 hrs curing, there are no obvious bending angle differences between the original ETAC and cured ETAC.
3) We also conducted magnetization profile measurements to further support our claim. As shown in Figure S69 A, an ETAC was magnetized in the vertical direction, and five measurement points were selected to record the magnetic strength change before and after bending actuation under the softening state. Figure S69 B shows no obvious changes in magnetic strength observed.

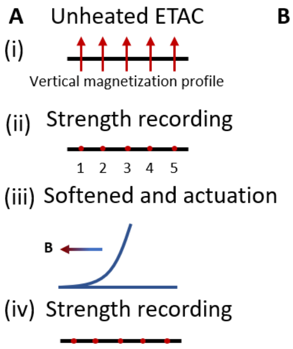


B

| Point No. | Strength before (mT) | Strength after (mT) |
|---|---|---|
| 1 | 8.57 | 8.66 |
| 2 | 7.94 | 7.92 |
| 3 | 8.12 | 8.09 |
| 4 | 7.71 | 7.80 |
| 5 | 6.75 | 6.62 |

**Figure S69.** Magnetic strength comparisons. (**A**) Experimental principle. (**B**) Quantitative comparison

## Section 20. Detailed explanation of self-division

As described in the main text, self-divisions can be completed manually (Figure 3E) or magnetically (Figure 3G). Figure S70 A presents manual and magnetic division comparisons. The manual approach is simple and direct, while the magnetic approach shows apparent advantages in performing remote division in confined lumens (Figure S70 B). The applied forces are marked in the figure. We note that the magnetic twisting is realizable when the holding force is sufficient by a stronger magnetic field strength.

Regarding the force application distance at the magnetic tip end, the control distance strongly depends on the magnetic field strength generated by the actuation device. According to our optimized test results (we optimized the permanent control distance for practical uses by improving the permanent magnet strength and improving ETAC's magnet particle content, resulting in an actuation distance more than 10 cm from the ETAC tip), the control distance for self-division ranges from 5 cm to 12 cm, which is suitable for various BME application scenarios.

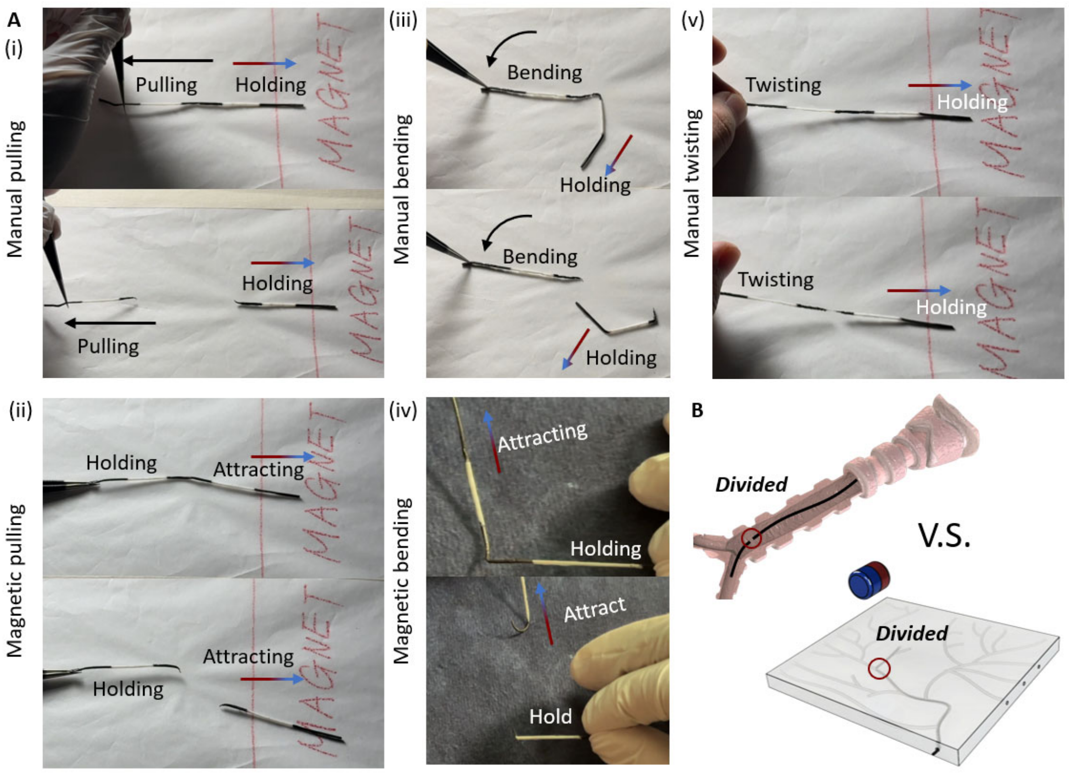


**Figure S70.** Comparisons between the manual and magnetic division approach. (**A**) (i-ii) pulling division, (iii-iv) bending division, and (v) twisting division. (**B**) Application scenarios for manual and magnetic divisions.

**Section 21. Self-alignment during the mergence process**

The alignment issue reveals ETAC's another advantage of conducting self-alignment due to the continuum's nature. We have conducted a series of division-mergence tests to showcase the self-alignment performances. Due to the continuous magnetization distribution, the divided segments can be regarded as multiple magnets that maintain their original magnetization profiles. Regardless of the division methods adopted (Figure S71) or the geometries at the division region, by bringing the two division ends close to a short distance, the divided two segments can attract and align with each other. We also observed that the two segments cannot easily align with each other due to environmental friction. This issue can be addressed by adjusting the main segment's position by manually pushing/pulling its remote end to shorten the distance between the two segments. During this process, no external magnetic field is required.

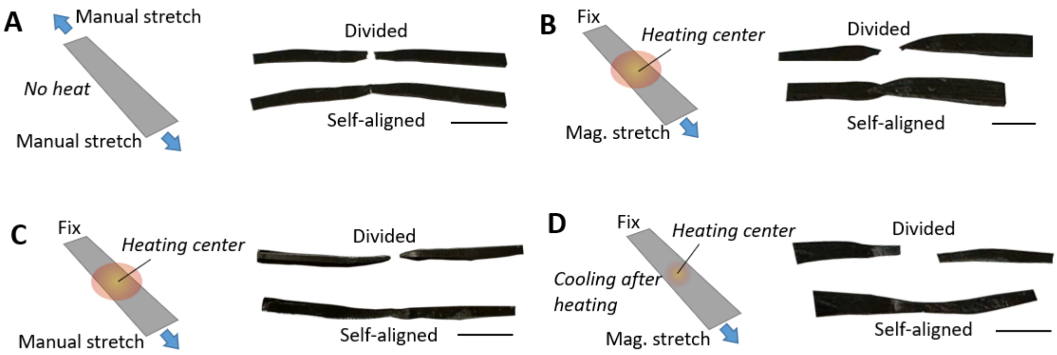


**Figure S71.** Self-alignment of separated segments. (**A**) Self-alignment of manually divided segments. (**B**) Self-alignment of magnetically divided segments after RF heating. (**C**) Self-alignment of manually divided segments after RF heating. (**D**) Self-alignment of magnetically divided segments after an RF heating-cooling loop.

**Section 22. Predictions of self-merged ETAM-devices**

The deformation prediction after ETACs' self-mergence is convenient and direct which is an advantage of ETAC's simple structure design. As described in the main text, F-regions and N-regions are respectively employed to conduct division/mergence tasks and deformation tasks, due to the RF-responsive capability of F-regions and the dipolar magnetic performances of N-regions. When F and N-regions are simultaneously under an external magnetic field, F regions tend to be attracted under the gradient magnetic field direction, while N-regions perform dipolar performances with respect to the field direction. Such deformations are directly predict by numerical simulations.

We newly calculated 3 cases for comparisons and illustrations. As shown in Figure S72, we preset 3 designs of self-merged structures with different magnetic profiles. Our developed FE model (shown in Supplementary Materials S5) successfully predicted the desired deformations.

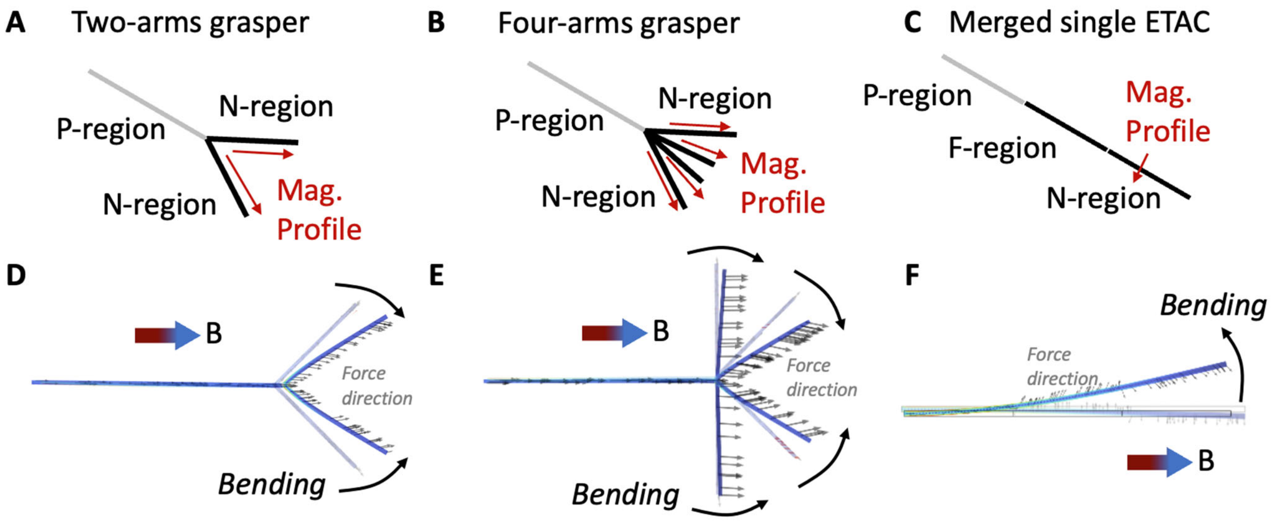


**Figure S72**. Actuation prediction of self-merged ETACs. (**A-C**) Different self-merged structures with various magnetization profiles. (**D-F**) Numerical simulation results showing the desired deformations.

**Section 23. Softening effect by preheating**

The softening effect by preheating is an interesting and unique phenomenon observed during ETAC testing and therefore utilized to further enhance the ETACs' functionality.

Given the phase change effect, ETAM respectively experiences reversible softening and hardening effects by heating and cooling within a few seconds to minutes (as shown in the top part of Figure S73). We defined the solidified, softened, and fluidified ETAMs as the elastomer state, softened state, and fluid state, respectively. However, we found certain types (flexible:rigid PCL powders≥ 2:1) of our customized ETAMs experienced an intermediate state between the elastomer state and softened state. We call these materials 4-states-ETAMs and the other types of ETAMs which only experience elastomer, softened and fluid states are called 3-states-ETAMs. By preheating 4-states-ETAMs to 60℃, the overall thermoplastic base materials can be melted. After cooling the fluidified 4-states-ETAMs to the ambient temperature within a few seconds, the 4-states-ETAMs will stay in the intermediate state for ~10 hours to cure (Figure S73). During this period, the 4-states-ETAMs remain at a stiffness that is higher than the softened stiffness and lower than the elastomer stiffness, showing enhanced flexibility for more delicate deformations.

When the terrains are too complex to navigate, it is necessary to conduct preheating to 4-states-ETAMs for enhanced flexibility. During the experiment ETACs are of ambient temperature and in the intermediate state.

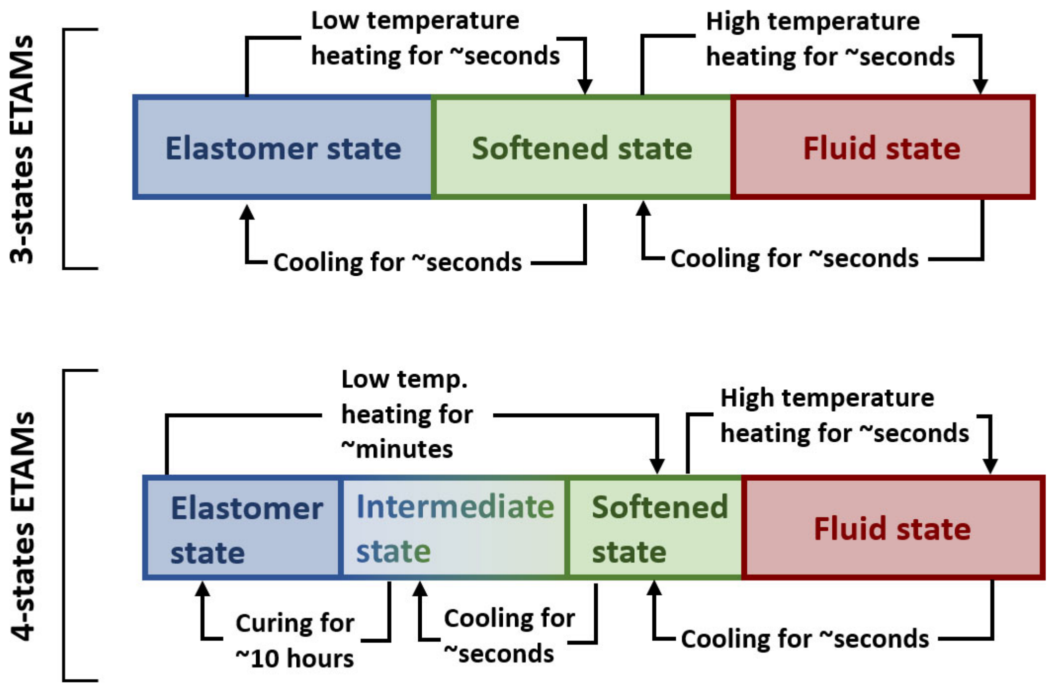


**Figure S73.** The phase transition process of 3-states ETAMs and 4-states-ETAMs.

**Section 24. In vivo meshing**

This demonstration aims to showcase that ETACs can navigate through a confined and curved lumen to an open space, where the ETAC can operate in vivo meshing for varied purposes, such as in situ tissue protection and in situ stent repairment. The ETAC navigated through a confined and curved lumen controlled by magnetic fields (Fig. S74 i). It is accurately targeted at a point on the inner wall of the open space, where the ETAC's tip can attach. An RF field was applied at the attachment region to soften the ETAC tip for adhesion (Fig. S74 ii). After cooling the attachment region for a few seconds, another RF field was applied to the end of the exposed ETAC for self-division (Fig. S74 iii). This process generated an untethered segment with one end bonded to the environment. By guiding the other end to the opposite target and conducting another RF mergence, the untethered segment can be fixed on the environment (Fig. S74 iv). By repeating the abovementioned steps multiple times, the in vivo meshing can be realized (Fig. S74 v-vi). This demonstration also suggests the possibility of in vivo printing, which follows a similar concept to in vivo meshing. The in vivo meshing demonstration holds the potential to conduct multiple point-ablations to realize submucosal lifting remotely, aiding the traditional ESU in completing ESD. It also has the potential to complete multiple regions of ablation and simultaneous lifting, as shown in Figure S75.

We present potential biomedical applications in Figure S76. Given the regrafting function, ETACs hold the potential to conduct procedures requiring passing through narrow bottlenecks, such as endoscopic retrograde cholangiopancreatography and transurethral procedures, which the common functional continuum cannot easily insert. ETACs also have the potential to operate minimally invasive surgeries for small-size animals, such as nasal passage intubation, RF ablation in the gastrointestinal tract (demonstrated in Figure 8), and respiratory system navigation. The abovementioned lumens are of narrow sizes ranging from 1 mm to 5 mm, which are challenging for existing continuum robots to navigate and operate complex tasks.

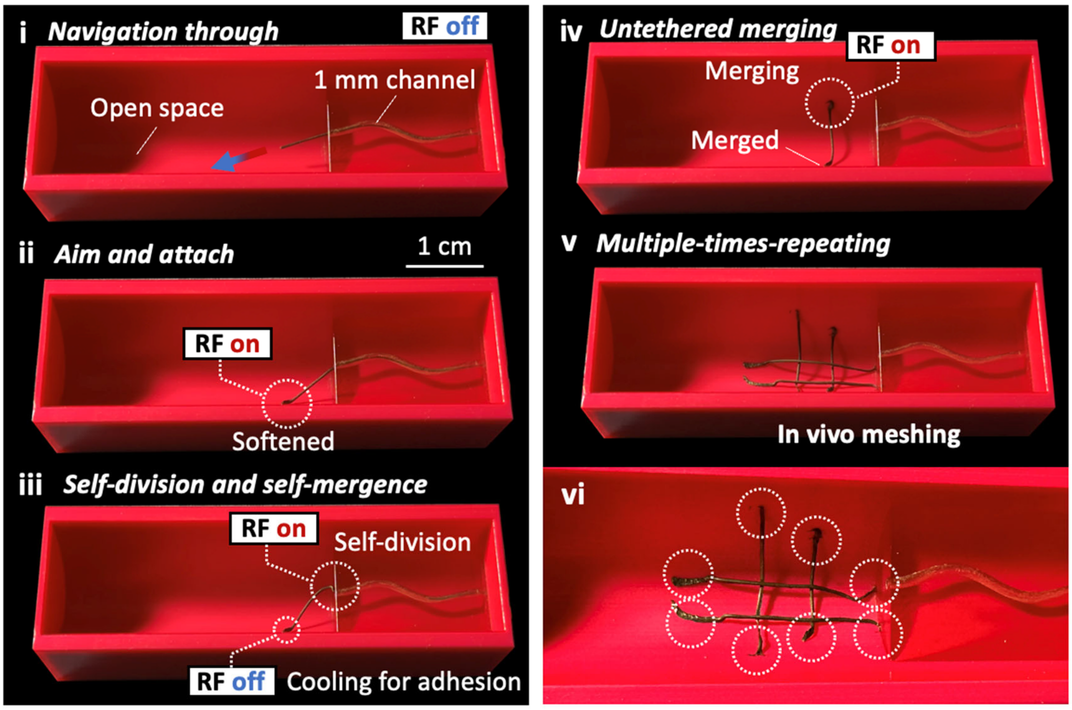


**Figure S74.** In vivo meshing. **(i)** ETAC navigated through a confined and curved lumen (with an inner diameter of 1 mm) to an open space. **(ii)** ETAC was magnetically guided to aim at the target point and approach by insertion. **(iii)** Self-division and self-mergence were used to bond the tip to the environment and split it into an untethered segment. **(iv)** Untethered merging

with the external environment. **(v)** In vivo meshing can be realized by repeating multiple times. **(vi)** Zoom-in view of the formed mesh.

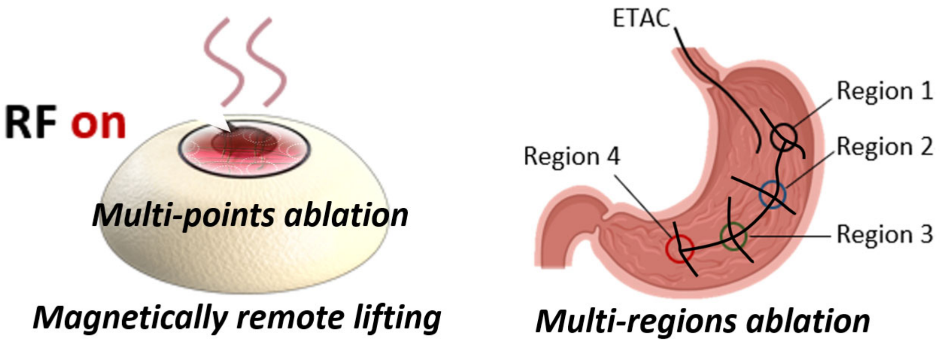


**Figure S75.** Potential applications of in vivo meshing demonstration.

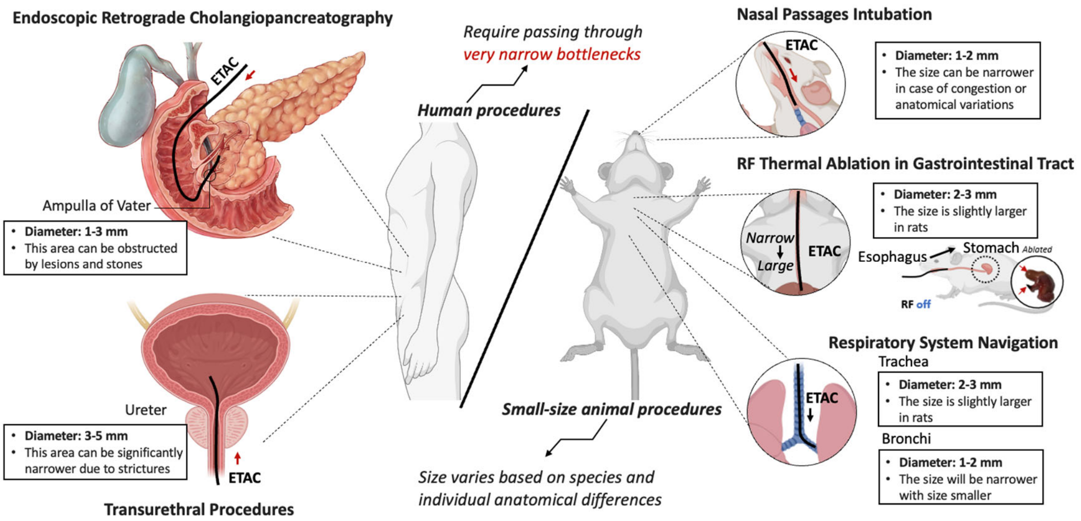


**Figure S76.** Biomedical application scopes of ETACs requiring passing through narrow bottlenecks. Human procedures include endoscopic retrograde cholangiopancreatography and transurethral procedures. Small-size animal procedures include nasal passage intubation, RF thermal ablation in the gastrointestinal tract, and respiratory system navigation. Created in BioRender. Yang, Y. (2025) https://BioRender.com/pbrbpao

Here, we would like to provide a more detailed explanation of why it is feasible to achieve self-mergence with the presence of body fluids or lubricants. Moreover, we set up a more stringent and extreme condition to support our claim: the in vivo meshing demonstration should be realized underwater, and the mesh should maintain its structure under external magnetic attractions.

There are two fundamental requirements to achieve self-mergence in a fluid environment: 1) keeping the heat accumulation rate higher than the heat dissipation rate and 2) applying an external force (i.e., magnetic force) to expel fluids from the space between contact interfaces. Regarding the first requirement, the feasibility of self-mergence in the presence of body fluids or lubricants strongly depends on the RF power. According to our test, under a 770 kHz RF field, the heat accumulation rate is higher than the heat dissipation rate. The RF frequency of existing commercial heaters can achieve GHz levels, which can provide more efficient heating with a relatively large heating distance. Regarding the second requirement, applying an external magnetic force during or immediately after RF heating leads to reliable mergence between ETACs or

between an ETAC and the environment. It should be noted that the necessity of applying external force depends on the heat dissipation rate, which is determined by the amount of body fluids or lubricants present.

As shown in Figure S77, the in vivo meshing underwater was successfully realized. The ETAC was inserted through a bottleneck terrain to a large space that was filled with water. The self-division can be conducted both in the air or underwater (as proven in the first version of the response letter). Under magnetic guidance, the separated ETAC can be guided to the target region. Both ends of the separated ETAC can firmly adhere to the phantom underwater by activating the RF heater and applying a downward magnetic force. The mesh can be constructed by repeating the above step three more times. To validate the structural stability of the mesh, we pumped the water out of the phantom and applied a permanent magnet to attract the structured mesh. The mesh successfully maintained its structure under the magnetic influence. Regarding removing the firmly structured mesh from the target area, we can activate the RF heating field while applying a repelling magnetic field to the mesh. The key principle of this process is to create tiny spaces between the EATC-environment interfaces for refilling fluids.

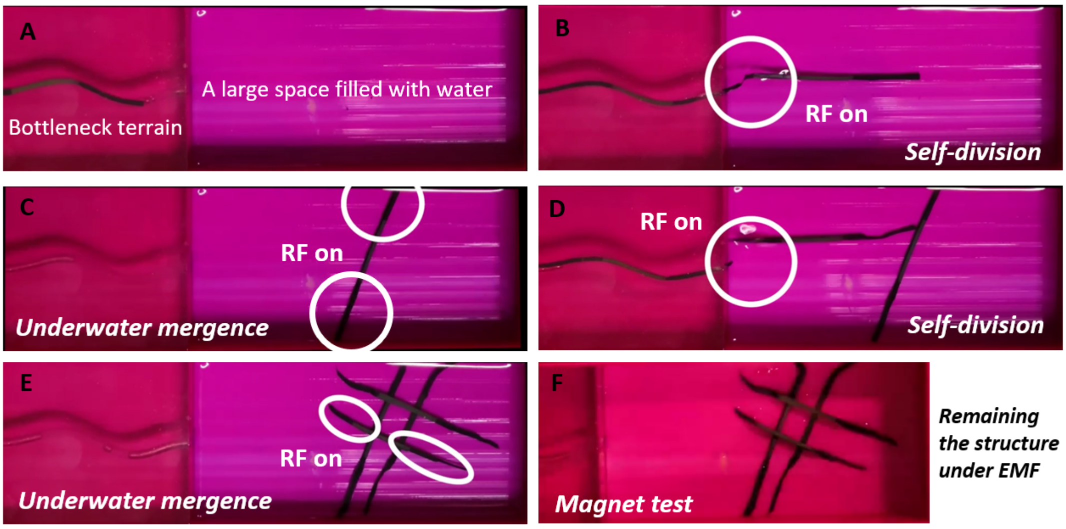


**Figure S77.** In vivo meshing underwater was successfully realized, proving that the presence of the body fluid or lubricants also allows the self-mergence.

**Section S25. Mobility of separated sub-ETACs and their alignment strategies**

In this section, we first investigated the separated ETACs' inherent mobility, realizing the active motion to desired locations. Next, using the established control strategy, the separated ETACs (untethered) can align accurately with the ETAC main body (tethered) for precise self-mergence. Therefore, the gripper demo has been updated with a deeper understanding of mobility characteristics and alignment strategy.

**The inherent mobility of separated ETACs and alignment strategy**

Multiple factors determine the control strategy and alignment of separated ETACs, including the magnetization profile, magnetic particle types, alignment locations, etc. Therefore, we have designed the experiment as shown in Table S4.

To eliminate the control instability associated with manual operation, we utilized a robot arm (Kuka LBR Med 7 R800) to manipulate an external permanent magnet according to the planned control strategy. Figure S78 illustrates the experimental setup. The distance between the magnet and ETACs ranges from 5~8 cm, which can be increased by using a bigger magnet with stronger magnetic field strength. Compared to Helmholtz coil control, we also note that applying an external permanent magnet provides a more convenient and direct method to generate a gradient field for ETACs' movement. An electromagnet field can also realize the same effect by preprogramming field changes.

**Table S4.** Case table of the experimental design

| Case No. | Magnetization direction | Particle type | Alignment location | Magnetization profile alignment |
|---|---|---|---|---|
| 1 | 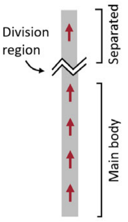  Axial | NdFeB | Face-to-face (F2F) | 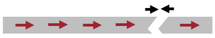 |
| 2 | | | | 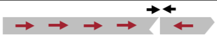 |
| 3 | | | Edge-to-edge (E2E) | 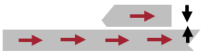 |
| 4 | | | | 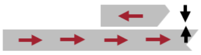 |
| 5 | | | Point-to-point (P2P) | 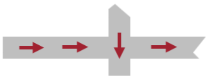 |
| 6 | 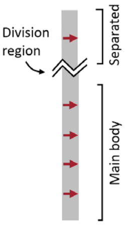  Radial | NdFeB | Face-to-face (F2F) | 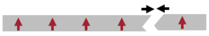 |
| 7 | | | | 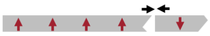 |
| 8 | | | Edge-to-edge (E2E) | 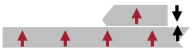 |
| 9 | | | | 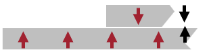 |
| 10 | | | Point-to-point (P2P) | 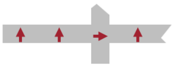 |
| 11 | | NdFeB | Face-to-face (F2F) | 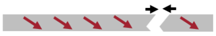 |
| 12 | | | | 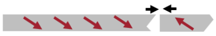 |
| 13 | | | Edge-to-edge (E2E) | 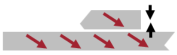 |

| | | | | |
|---|---|---|---|---|
| 14 | 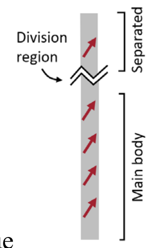 Oblique | | | 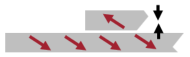 |
| 15 | | | Point-to-point (P2P) | 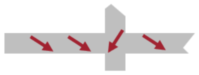 |
| 16 | 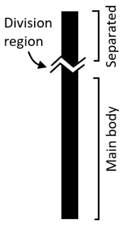 No dir. | $Fe_3O_4$ | Face-to-face (F2F) | 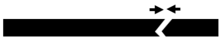 |
| 17 | | | Edge-to-edge (E2E) | 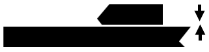 |
| 18 | | | Point-to-point (P2P) | 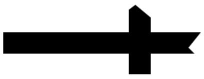 |
| 19 | 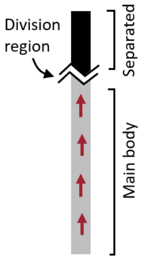 Axial | NdFeB vs. $Fe_3O_4$ | Face-to-face (F2F) | 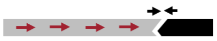 |
| 20 | | | Edge-to-edge (E2E) | 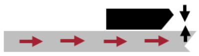 |
| 21 | | | Point-to-point (P2P) | 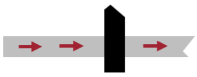 |

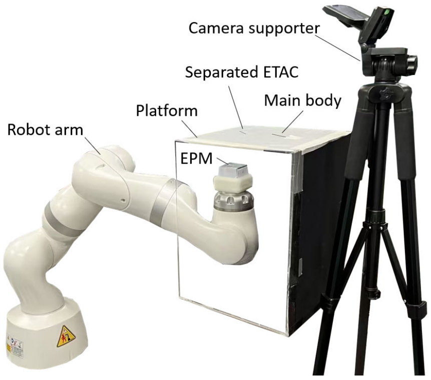


**Figure S78**. Experimental setup

Figure S79 shows all experimental results and the corresponding control strategies. We demonstrate how a separate (untethered) ETAC is magnetically guided to align with a tethered ETAC main body. The untethered-tethered ETAC alignment is regarded as the most common scenario for self-mergence. Typically, the main body is tethered and inserted manually or via an insertion mechanism, and a single separated ETAC is preferred rather than multiple separated ETACs for better controllability. Regarding the scenario of multiple separated ETACs aligning with a tethered ETAC, we believe it can be further realized for more specific tasks in future works. In this work, we would like to discuss solely the alignment of a single untethered ETAC with the main body. This approach is sufficient to support all the demonstrations presented in the main text.

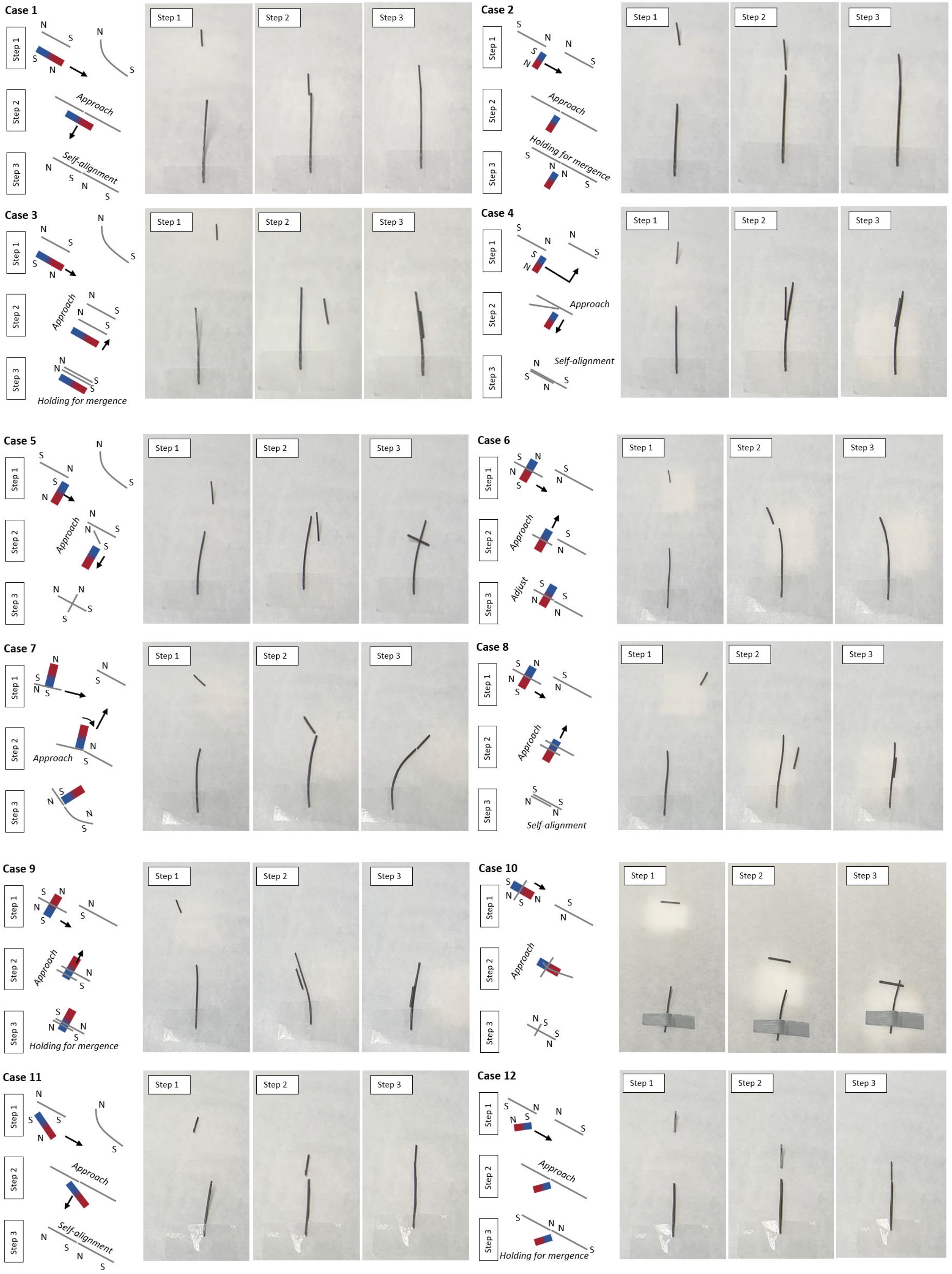

Case 1
Step 1
Step 2
Step 3
Approach
Self-alignment
Case 2
Approach
Holding for mergence
Case 3
Approach
Holding for mergence
Case 4
Approach
Self-alignment
Case 5
Approach
Case 6
Approach
Adjust
Case 7
Approach
Case 8
Approach
Self-alignment
Case 9
Approach
Holding for mergence
Case 10
Approach
Case 11
Approach
Self-alignment
Case 12
Approach
Holding for mergence

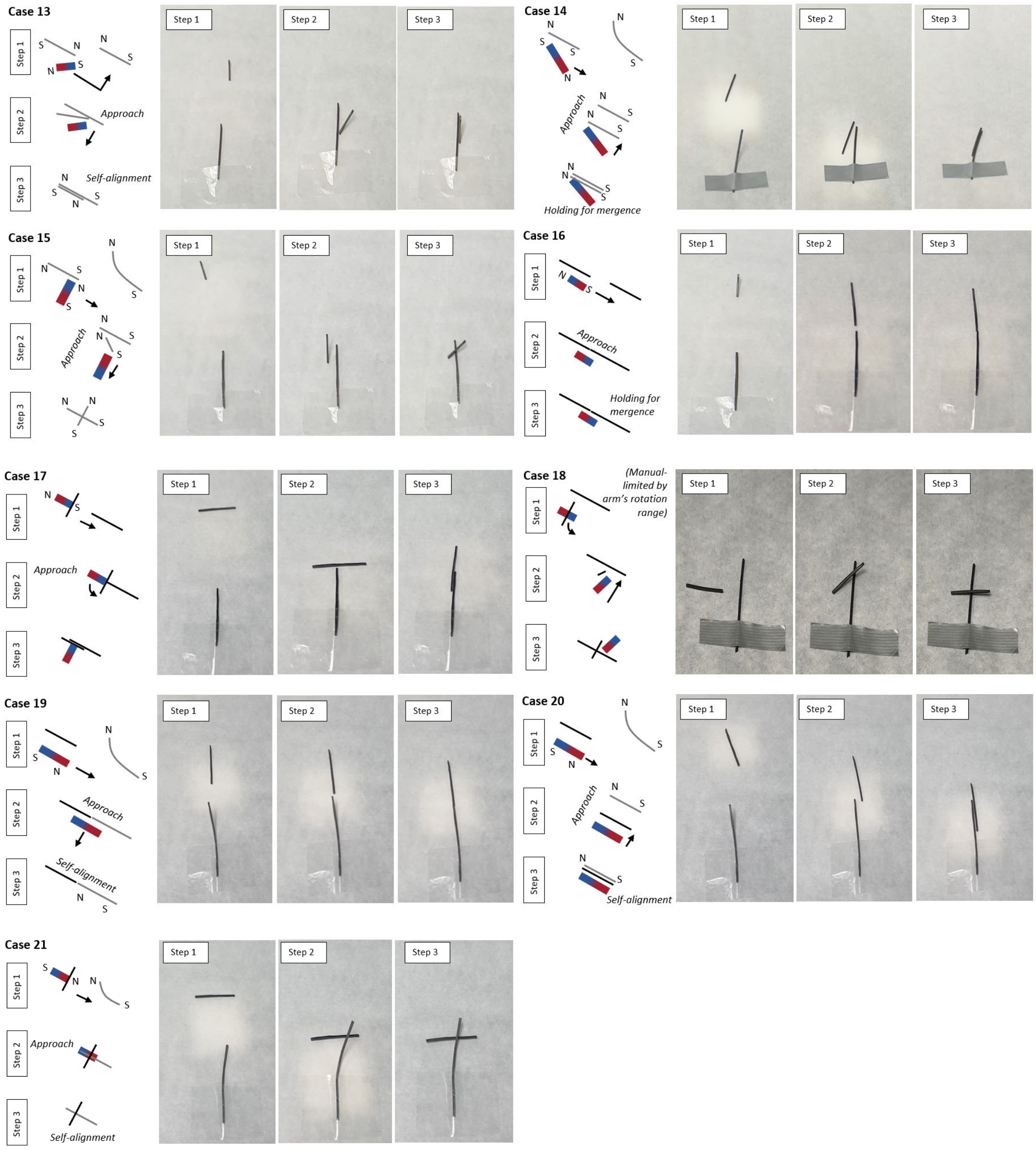


**Figure S79**. Experimental results of separated ETACs' mobility characteristics and alignment strategy

The results indicate that there are three basic principles for conducting alignment: 1) self-alignment by utilizing the same magnetization direction (e.g., Case 1, 4, 8, 11, 13, 19, 20, 21), 2) holding for mergence by utilizing external magnetic fields to counteract repulsion (e.g., Case 2, 3, 9, 12, 14, 16), and 3) neglectable magnetization influence (e.g., Case 5, 6, 7, 10, 15, 17, 18). Each case was conducted at least three times to ensure repeatability.

**Section S26. Self-division strategies and their stability analysis**

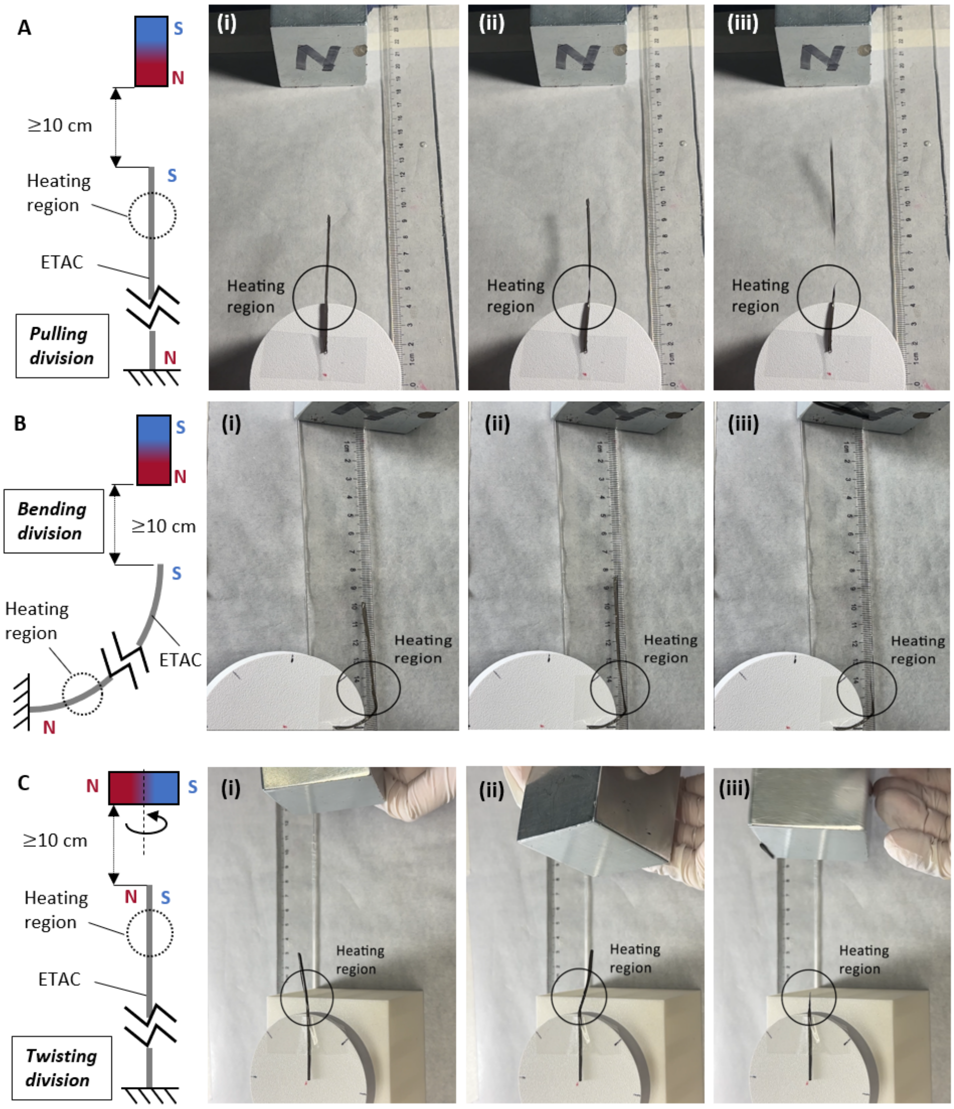


**Figure S80.** Self-division strategies including: (**A**) pulling division, (**B**) bending division, and (**C**) twisting division.

The division processes are also shown in Supplementary Movie S20. It can be seen that the actuation distance between the permanent magnet and ETAC is more than 10 cm, which is consistent with our claim and the self-divisions have been successfully realized as the planned strategies. The actuation distance can be further increased by increasing the magnet size and the magnetic nanoparticle proportions of the ETAC.

Although all division strategies were successfully realized, we found that self-division is more complicated to conduct than self-emergence. There are two reasons: 1) compared with the self-emergence that commonly requires a single RF heating field, the self-division requires both heating and external magnetic moment for splitting. 2) The magnetic moment generated by the field is required to be relatively large for division. Moreover, a stable rotational magnetic field with an appropriate rotation speed is required in specific cases, such as twisting division. Regarding the large bending back-and-forth in Figure 3G and its corresponding movie, such a phenomenon was also avoided in the abovementioned demonstration. The large bending back-and-forth was led by the insufficient heating temperature and the relatively low magnetic strength of the utilized small

permanent magnet. By increasing the heating power and utilizing a bigger permanent magnet, the large bending back-and-force was avoided, as shown in Figure S80 and Supplementary Movie S20.

**Section 27. Potential biomedical scenarios and their corresponding required actuation distances**

We have provided a detailed list showing the potential biomedical scenarios that could satisfy such actuation distance, as shown in Table S5.

**Table S5.** Potential biomedical scenarios and their corresponding required actuation distances.

| **Potential biomedical scenarios** | **Required actuation distance** | **Supporting literature** |
|---|---|---|
| Trachea & bronchi navigation (Navigation and regrafting) | Skin-trachea mean distance 9.2±1.9 mm<br>Skin-upper trachea distance ~9.2 mm<br>Skin-lower trachea distance ~30.5 mm | Bermede, O., Sarıcaoğlu, M. C., Baytaş, V., Hasde, A. İ., İnan, M. B., & Akar, A. R. (2021). Percutaneous ultrasound-guided versus bronchoscopy-guided dilatational tracheostomy after median sternotomy: A case-control study. Turkish Journal of Thoracic and Cardiovascular Surgery, 29(4), 457. |
| Endoscopic retrograde cholangiopancreatography (Navigation and regrafting) | Skin-ampulla of Vater mean distance 65 mm | Peng, D., Tao, W., Cheng, Y., Zou, Y. Y., Qian, K., & Zhang, W. (2020). The Shortest Distance from the Skin to Pancreas and the Lower Sternum Angle can Influence Short-Term Surgical Outcomes of Laparoscopy-Assisted Distal Gastrectomy for Gastric Cancer. |
| Transurethral procedures (Navigation) | Skin-ureter mean distance 83.2 mm | Shan, C. J., Mazzucchi, E., Payão, F., Gomes, A. C., Baroni, R. H., Torricelli, F. C., ... & Srougi, M. (2014). The skin-to-calyx distance measured by renal ct scan and ultrasound. International braz j urol, 40, 212-219. |
| Endoscopic submucosal dissection (Navigation and regrafting) | Skin-stomach mean distance 30.9 mm<br>Skin-stomach maximum distance 52 mm | Kiran, G., Yilmaz, I., Aydin, S. E. R. D. A. R., Sanlikan, F., & Ozkaya, E. (2022). The shortest distance between the skin and the peritoneal cavity is obtained with fascial elevation: a preliminary prospective laparoscopic entry study. Facts, Views & Vision in Obgyn, 14(2), 171. |
| Small-size animal nasal passage intubation, RF thermal ablation in the gastrointestinal tract, respiratory system navigation | Mean diameter of mice nasal passage 1-2 mm<br>Mean diameter of mice esophagus 1-2 mm<br>Mean diameter of mice trachea 1-2 mm<br>Mean diameter of mice bronchi 1-2 mm | Alvites, R. D., Caseiro, A. R., Pedrosa, S. S., Branquinho, M. E., Varejão, A. S., & Maurício, A. C. (2018). The nasal cavity of the rat and mouse—source of mesenchymal stem cells for treatment of peripheral nerve injury. The Anatomical Record, 301(10), 1678-1689. Jelvehgaran, P., de Bruin, D. M., Khmelinskii, A., Borst, G., Steinberg, J. D., Song, J. Y., ... & van Herk, M. (2019). Optical coherence |

| | | |
|---|---|---|
| | | tomography to detect acute esophageal radiation-induced damage in mice: A validation study. Journal of biophotonics, 12(9), e201800440.<br>Kishimoto, K., & Morimoto, M. (2021). Mammalian tracheal development and reconstruction: insights from in vivo and in vitro studies. Development, 148(13), dev198192. |

## References for Supplementary Materials


1. Chen, C. K., Chou, H. P., & Sheu, M. H. (2013). Image-guided lung tumor ablation: Principle, technique, and current status. *Journal of the Chinese Medical Association*, *76*(6), 303-311.
2. Welter, S., Cheufou, D., Sommerwerck, U., Maletzki, F., & Stamatis, G. (2012). Changes in lung function parameters after wedge resections: a prospective evaluation of patients undergoing metastasectomy. *Chest*, *141*(6), 1482-1489.
3. Hiraki, T., Mimura, H., Gobara, H., Sano, Y., Fujiwara, H., Iguchi, T., ... & Kanazawa, S. (2009). Two cases of needle-tract seeding after percutaneous radiofrequency ablation for lung cancer. *Journal of Vascular and Interventional Radiology*, *20*(3), 415-418.
4. McTaggart, R. A., & Dupuy, D. E. (2007). Thermal ablation of lung tumors. *Techniques in Vascular and Interventional Radiology*, *10*(2), 102-113.
5. Brace, C. L., Hinshaw, J. L., & Lubner, M. G. (2011). Thermal ablation for the treatment of abdominal tumors. *JoVE (Journal of Visualized Experiments)*, (49), e2596.
6. Tajiri, N., Hiraki, T., Mimura, H., Gobara, H., Mukai, T., Hase, S., ... & Kanazawa, S. (2008). Measurement of pleural temperature during radiofrequency ablation of lung tumors to investigate its relationship to occurrence of pneumothorax or pleural effusion. *Cardiovascular and interventional radiology*, *31*, 581-586.
7. Sawada, M., Watanabe, S., Tsuda, H., & Kano, T. (2002). An increase in body temperature during radiofrequency ablation of liver tumors. *Anesthesia & Analgesia*, *94*(6), 1416-1420.
8. Liu, B. D., & Zhi, X. Y. (2015). Expert consensus on image-guided radiofrequency ablation of pulmonary tumors—2015 edition. *Annals of Translational Medicine*, *3*(9).
9. Kato, T., Okumura, I., Song, S. E., Golby, A. J., & Hata, N. (2014). Tendon-driven continuum robot for endoscopic surgery: Preclinical development and validation of a tension propagation model. *IEEE/ASME Transactions on Mechatronics*, 20(5), 2252-2263.
10. Wang, F., Wang, H., Luo, J., Kang, X., Yu, H., Lu, H., ... & Jia, X. (2021). FIORA: A flexible tendon-driven continuum manipulator for laparoscopic surgery. *IEEE robotics and automation letters*, 7(2), 1166-1173.
11. Amanov, E., Nguyen, T. D., & Burgner-Kahrs, J. (2021). Tendon-driven continuum robots with extensible sections—A model-based evaluation of path-following motions. *The International Journal of Robotics Research*, 40(1), 7-23.
12. Ba, W., Dong, X., Mohammad, A., Wang, M., Axinte, D., & Norton, A. (2021). Design and validation of a novel fuzzy-logic-based static feedback controller for tendon-driven continuum robots. *IEEE/ASME Transactions on Mechatronics*, 26(6), 3010-3021.
13. Huang, X., Zou, J., & Gu, G. (2021). Kinematic modeling and control of variable curvature soft continuum robots. *IEEE/ASME Transactions on Mechatronics*, 26(6), 3175-3185.
14. Yang, Y., Lai, J., Xu, C., He, Z., Jiao, P., & Ren, H. (2024). Lightweight Pneumatically Elastic Backbone Structure with Modular Construction and Nonlinear Interaction for Soft Actuators. *Soft Robotics*, 11(1), 57-69.
15. Li, Y., Ren, T., Chen, Y., & Chen, M. Z. (2020, May). A variable stiffness soft continuum robot based on pre-charged air, particle jamming, and origami. *In 2020 IEEE International Conference on Robotics and Automation (ICRA)* (pp. 5869-5875). IEEE.
16. Zhang, T., Yang, L., Yang, X., Tan, R., Lu, H., & Shen, Y. (2021). Millimeter-scale soft continuum robots for large-angle and high-precision manipulation by hybrid actuation. *Advanced Intelligent Systems*, 3(2), 2000189.
17. Wang, L., Zheng, D., Harker, P., Patel, A. B., Guo, C. F., & Zhao, X. (2021). Evolutionary design of magnetic soft continuum robots. *Proceedings of the National Academy of Sciences*, 118(21), e2021922118.
18. Kim, K., Lee, H. J., Yang, E. A., Kim, H. S., Chun, Y. H., Yoon, J. S., ... & Kim, J. T. (2018). Foreign body removal by flexible bronchoscopy using retrieval basket in children. *Annals of thoracic medicine*, 13(2), 82-85.
19. Kim, K., Lee, H. J., Yang, E. A., Kim, H. S., Chun, Y. H., Yoon, J. S., ... & Kim, J. T. (2018). Foreign body removal by flexible bronchoscopy using retrieval basket in children. *Annals of thoracic medicine*, 13(2), 82-85.
20. Inoue, T., Ibusuki, M., Kitano, R., Sakamoto, K., Kimoto, S., Kobayashi, Y., ... & Yoneda, M. (2023). Comparison of the mechanical properties of retrieval basket catheters for bile duct stones: An experimental study. *Indian Journal of Gastroenterology*, 42(5), 651-657.
21. Do, P. T., Le, Q. N., Luong, Q. V., Kim, H. H., Park, H. M., & Kim, Y. J. (2023, April). Tendon-driven gripper with variable stiffness joint and water-cooled SMA springs. In *Actuators* (Vol. 12, No. 4, p. 160). MDPI
22. Cursi, F., Modugno, V., & Kormushev, P. (2020, October). Model predictive control for a tendon-driven surgical robot with safety constraints in kinematics and dynamics. *In 2020 IEEE/RSJ International Conference on Intelligent Robots and Systems* (IROS) (pp. 7653-7660). IEEE.

23. Fang, B., Sun, F., Wu, L., Liu, F., Wang, X., Huang, H., ... & Wen, L. (2022). Multimode grasping soft gripper achieved by layer jamming structure and tendon-driven mechanism. *Soft Robotics*, 9(2), 233-249.

24. Gao, H., Yang, X., Xiao, X., Zhu, X., Zhang, T., Hou, C., ... & Ren, H. (2024). Transendoscopic flexible parallel continuum robotic mechanism for bimanual endoscopic submucosal dissection. *The International Journal of Robotics Research*, 43(3), 281-304.

25. Li, W., Shen, M., Gao, A., Yang, G. Z., & Lo, B. (2020). Towards a snake-like flexible robot for endoscopic submucosal dissection. *IEEE Transactions on Medical Robotics and Bionics*, 3(1), 257-260.

26. Cao, L., Li, X., Phan, P. T., Tiong, A. M. H., Kaan, H. L., Liu, J., ... & Phee, S. J. (2020). Sewing up the wounds: A robotic suturing system for flexible endoscopy. *IEEE Robotics & Automation Magazine*, 27(3), 45-54.

27. Yang, Z., Yang, H., Cao, Y., Cui, Y., & Zhang, L. (2023). Magnetically actuated continuum medical robots: a review. *Advanced intelligent systems*, 5(6), 2200416.

28. Shah, D. S., Powers, J. P., Tilton, L. G., Kriegman, S., Bongard, J., & Kramer-Bottiglio, R. (2021). A soft robot that adapts to environments through shape change. *Nature Machine Intelligence*, 3(1), 51-59.

29. Hann, S. Y., Cui, H., Nowicki, M., & Zhang, L. G. (2020). 4D printing soft robotics for biomedical applications. *Additive Manufacturing*, 36, 101567.

30. Laeseke, P. F., Sampson, L. A., Haemmerich, D., Brace, C. L., Fine, J. P., Frey, T. M., ... & Lee Jr, F. T. (2005). Multiple-electrode radiofrequency ablation: simultaneous production of separate zones of coagulation in an in vivo porcine liver model. *Journal of vascular and interventional radiology*, 16(12), 1727-1735.

31. Horiuchi, A., Nakayama, Y., Kajiyama, M., Kato, N., Kamijima, T., Graham, D. Y., & Tanaka, N. (2010). Biliary stenting in the management of large or multiple common bile duct stones. *Gastrointestinal endoscopy*, 71(7), 1200-1203.

32. Islam, S. R., Islam, E. A., Hodges, D., Nugent, K., & Parupudi, S. (2010). Endoscopic removal of multiple duodenum foreign bodies: an unusual occurrence. World Journal of *Gastrointestinal Endoscopy*, 2(5), 186.

33. Bajaj, D., Sachdeva, A., & Deepak, D. (2021). Foreign body aspiration. Journal of Thoracic Disease, 13(8), 5159.

34. Varshney, R., Zawawi, F., Shapiro, A., & Lacroix, Y. (2014). Use of an endoscopic urology basket to remove bronchial foreign body in the pediatric population. International Journal of Pediatric Otorhinolaryngology, 78(4), 687-689.

35. Wankhede, R. G., Maitra, G., Pal, S., Ghoshal, A., & Mitra, S. (2017). Successful removal of foreign body bronchus using C-arm-guided insertion of Fogarty catheter through plastic bead. Indian Journal of Critical Care Medicine, 21(2), 96.

36. Avdievich, N. I. . (2011). Transverse Electromagnetic (TEM) Surface Coils for Extremities. John Wiley & Sons, Ltd.

37. Vaughan, J. T., Adriany, G., Snyder, C. J., Tian, J., Thiel, T., Bolinger, L., ... & Ugurbil, K. (2004). Efficient high-frequency body coil for high-field MRI. Magnetic Resonance in Medicine: An Official Journal of the International Society for Magnetic Resonance in Medicine, 52(4), 851-859.

38. Murbach, M., Neufeld, E., Kainz, W., Pruessmann, K. P., & Kuster, N. (2014). Whole-body and local RF absorption in human models as a function of anatomy and position within 1.5 T MR body coil. Magnetic resonance in medicine, 71(2), 839-845.

39. Black, D. R., & Heynick, L. N. (2003). Radiofrequency (RF) effects on blood cells, cardiac, endocrine, and immunological functions. Bioelectromagnetics, 24(S6), S187-S195.

40. Wang, S., Zheng, M., Lou, C., Chen, S., Guo, H., Gao, Y., ... & Shang, P. (2022). Evaluating the biological safety on mice at 16 T static magnetic field with 700 MHz radio-frequency electromagnetic field. Ecotoxicology and Environmental Safety, 230, 113125.
