# Supplementary material for: Regrafting submillimeter-scale ferromagnetic soft continuums
Source: Nat Commun. 2025 Jul 31;16:7023. doi: 10.1038/s41467-025-60928-6 (PMC12313971; doi:10.1038/s41467-025-60928-6)
Supplement: Supplementary file 2 — Description of Additional Supplementary Files [file 41467_2025_60928_MOESM2_ESM.pdf]

Description of Additional Supplementary Files

File Name: Supplementary Movie 1
Description: Fabrications of ETACs

File Name: Supplementary Movie 2
Description: Comparisons between N-tips with and without graded stiffness

File Name: Supplementary Movie 3
Description: Navigation tests

File Name: Supplementary Movie 4
Description: Bronchi phantom tests

File Name: Supplementary Movie 5
Description: Ex vivo bronchi navigation

File Name: Supplementary Movie 6
Description: Self-divisions with different strategies

File Name: Supplementary Movie 7
Description: Local heating tests under RF heating

File Name: Supplementary Movie 8
Description: ETAMs-branch system with self-divisions

File Name: Supplementary Movie 9
Description: In vivo object grasping and releasing with the ETAMs-branch system

File Name: Supplementary Movie 10
Description: Continuums carriers

File Name: Supplementary Movie 11
Description: Airway foreign body removal

File Name: Supplementary Movie 12
Description: ETAMs-aided submucosal dissection

File Name: Supplementary Movie 13
Description: Buckling phenomenon

File Name: Supplementary Movie 14
Description: Graded-stiffness effect on buckling

File Name: Supplementary Movie 15

Description: Navigation under electromagnetic fields

File Name: Supplementary Movie 16
Description: Self-division by manual and magnetic approaches

File Name: Supplementary Movie 17
Description: In vivo meshing

File Name: Supplementary Movie 18
Description: In situ grasper assembling

File Name: Supplementary Movie 19
Description: Self-alignment

File Name: Supplementary Movie 20
Description: Self-division process under external permanent magnet generated fields
