# Supplementary figures and images for: Regrafting submillimeter-scale ferromagnetic soft continuums
Source: Nat Commun. 2025 Jul 31;16:7023. doi: 10.1038/s41467-025-60928-6 (PMC12313971; doi:10.1038/s41467-025-60928-6)

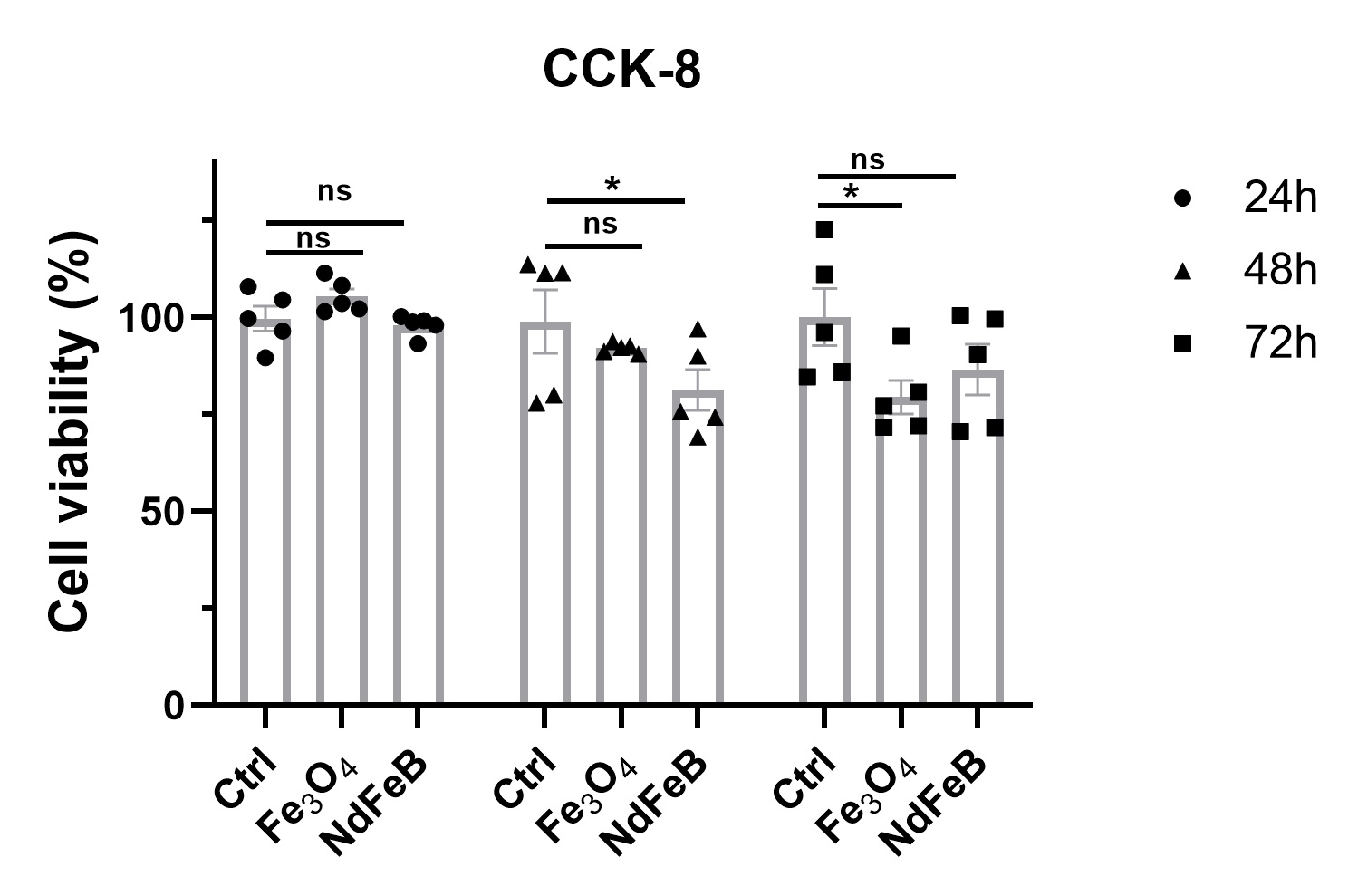

Supplement: Supplementary file 25 — Source Data [file 41467_2025_60928_MOESM25_ESM.zip › Source File/Fig. S37-38/Archive/CCK8/CCK8.jpeg]

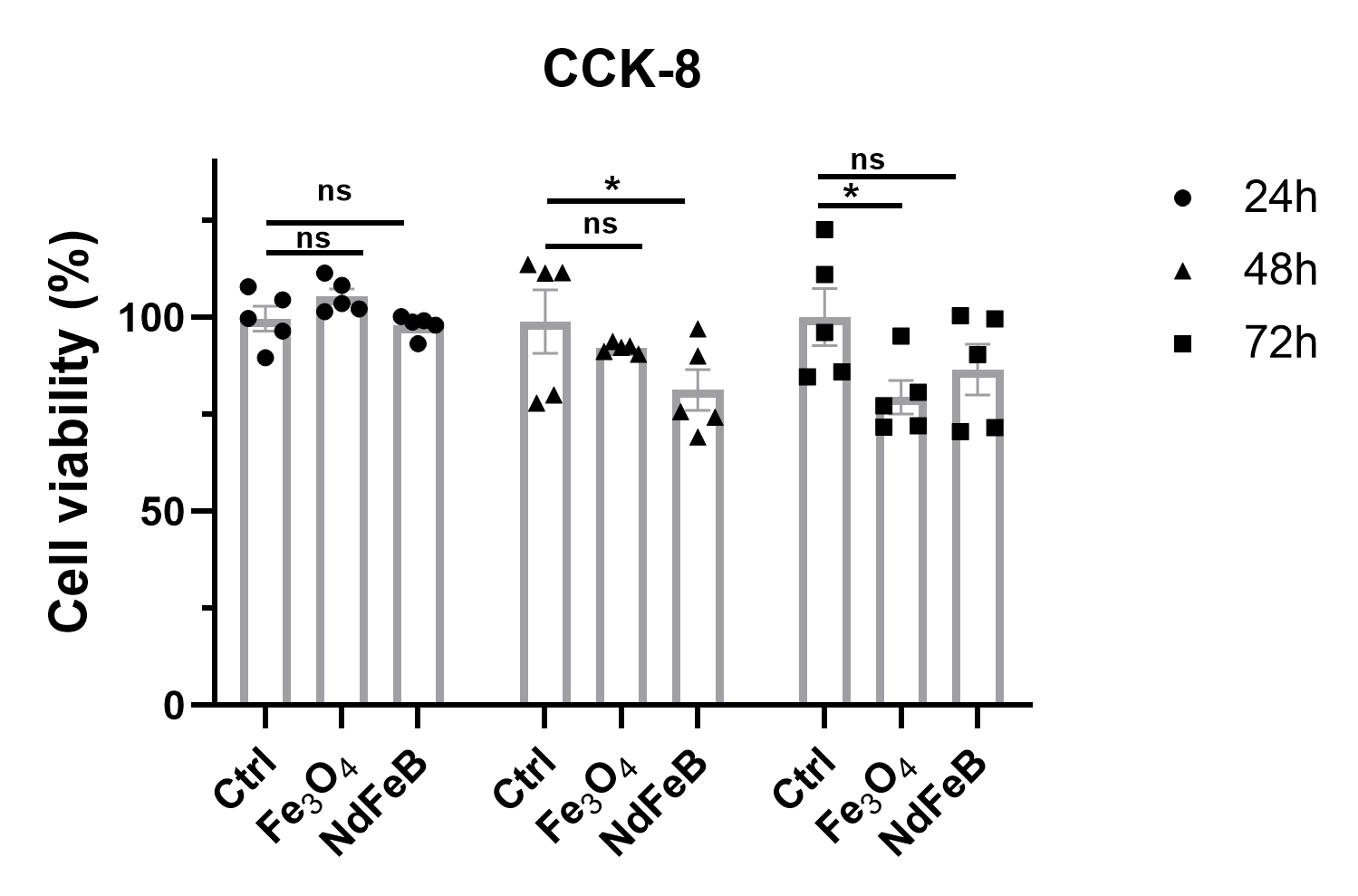

Supplement: Supplementary file 25 — Source Data [file 41467_2025_60928_MOESM25_ESM.zip › Source File/Fig. S37-38/Archive/CCK8/CCK8.tif]

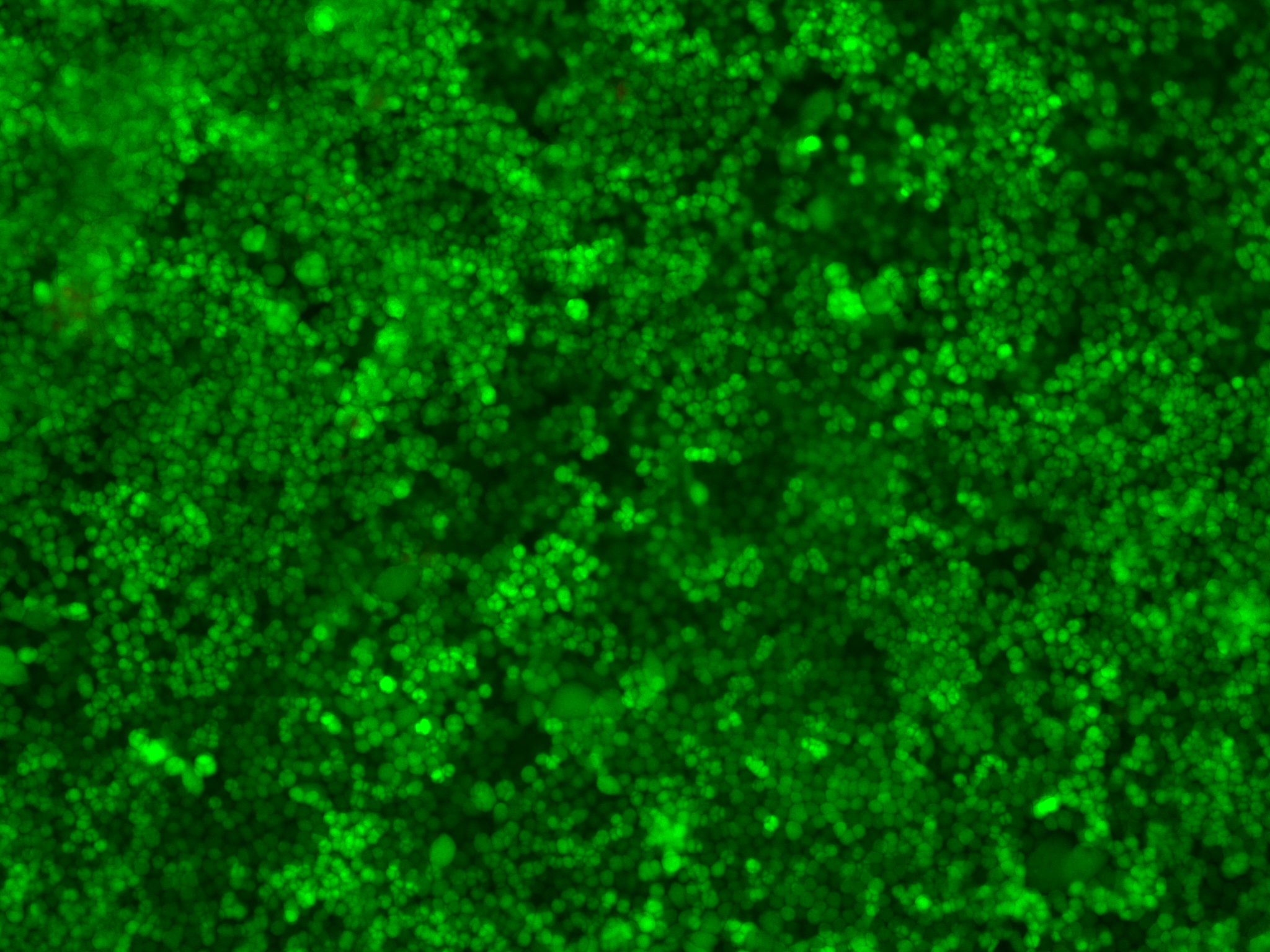

Supplement: Supplementary file 25 — Source Data [file 41467_2025_60928_MOESM25_ESM.zip › Source File/Fig. S37-38/Archive/staining/1023livedead-staining/ctrl/1.2.tiff]

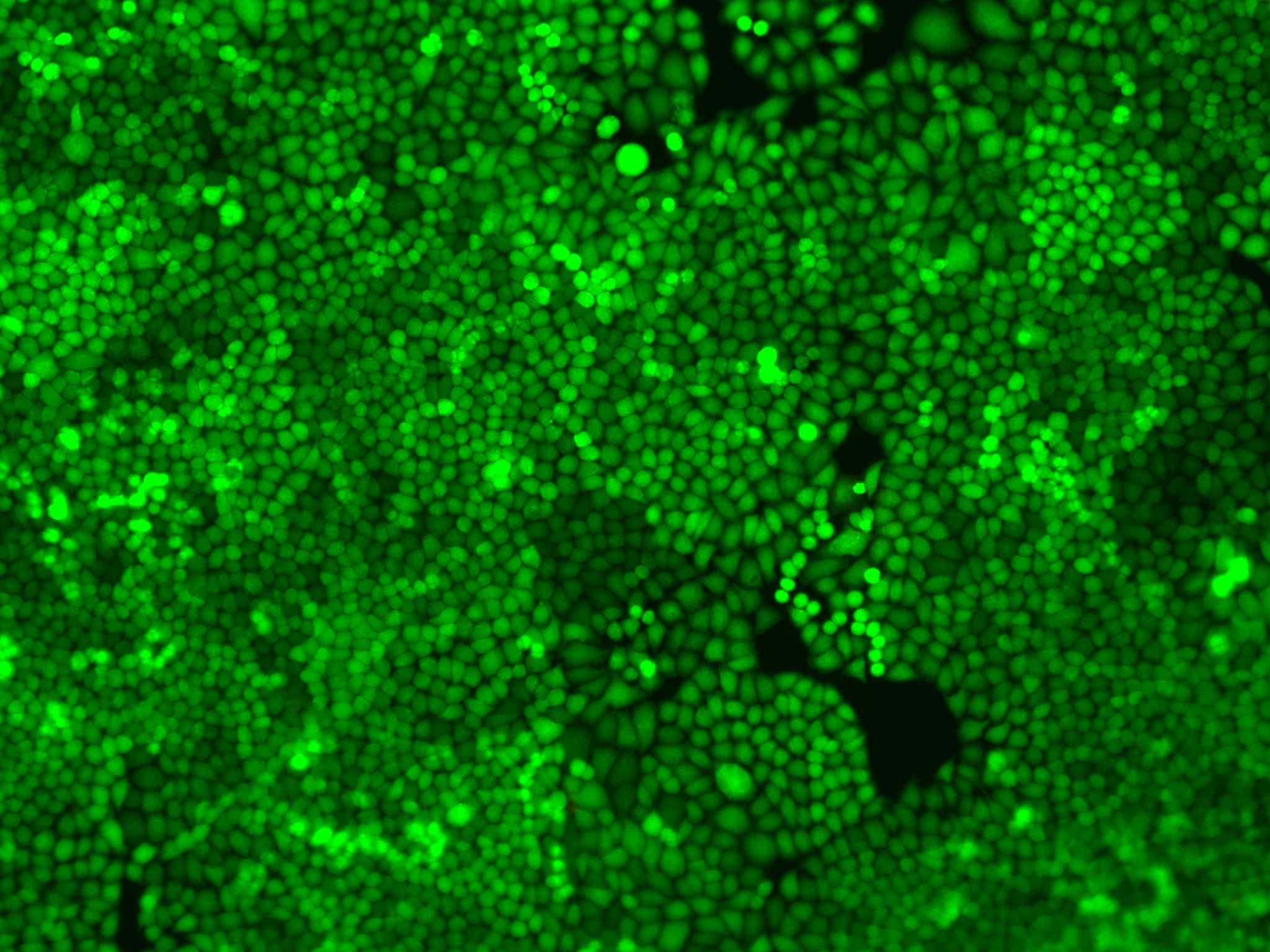

Supplement: Supplementary file 25 — Source Data [file 41467_2025_60928_MOESM25_ESM.zip › Source File/Fig. S37-38/Archive/staining/1023livedead-staining/ctrl/1.3.tiff]

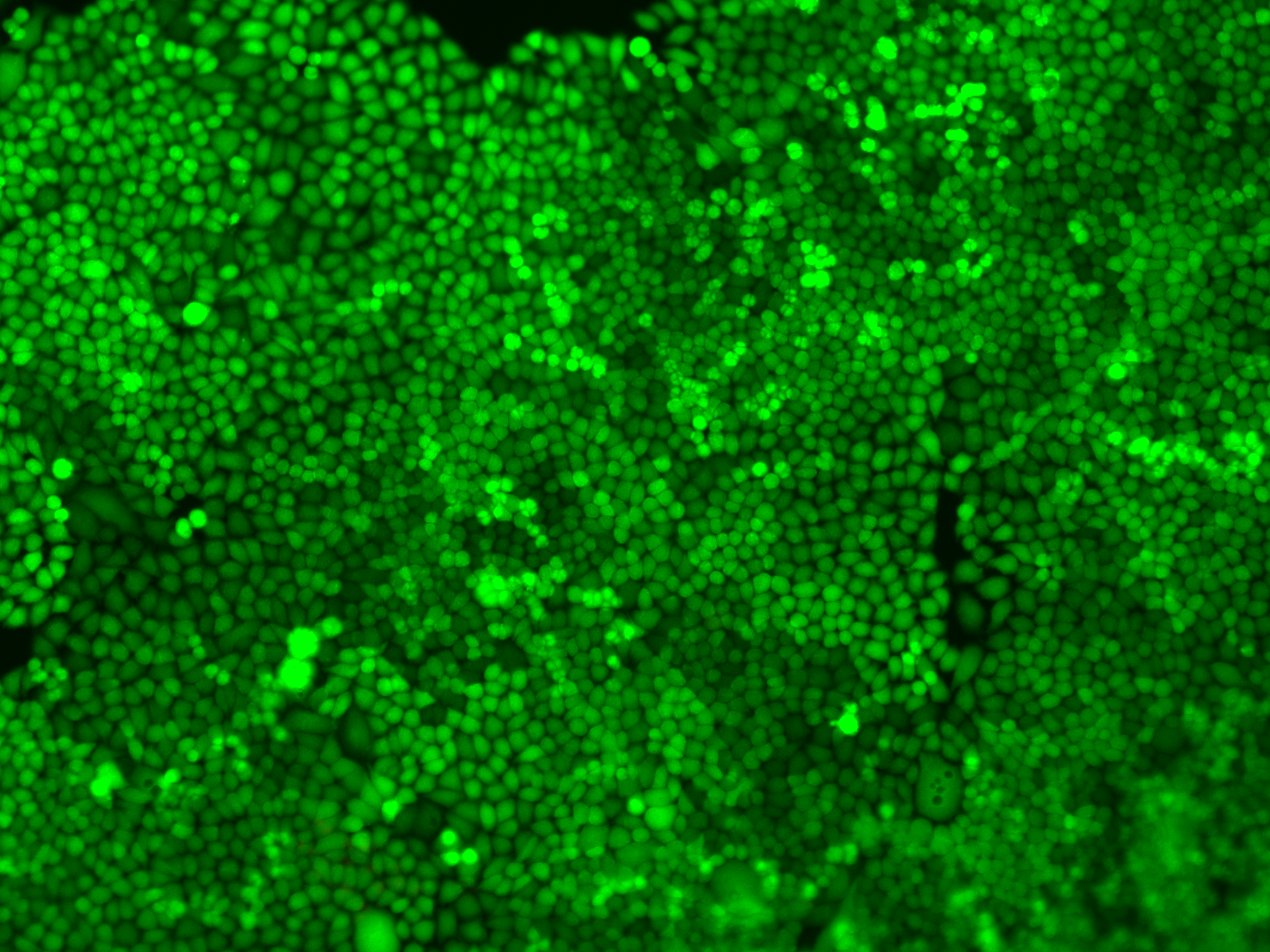

Supplement: Supplementary file 25 — Source Data [file 41467_2025_60928_MOESM25_ESM.zip › Source File/Fig. S37-38/Archive/staining/1023livedead-staining/ctrl/1.4.tiff]

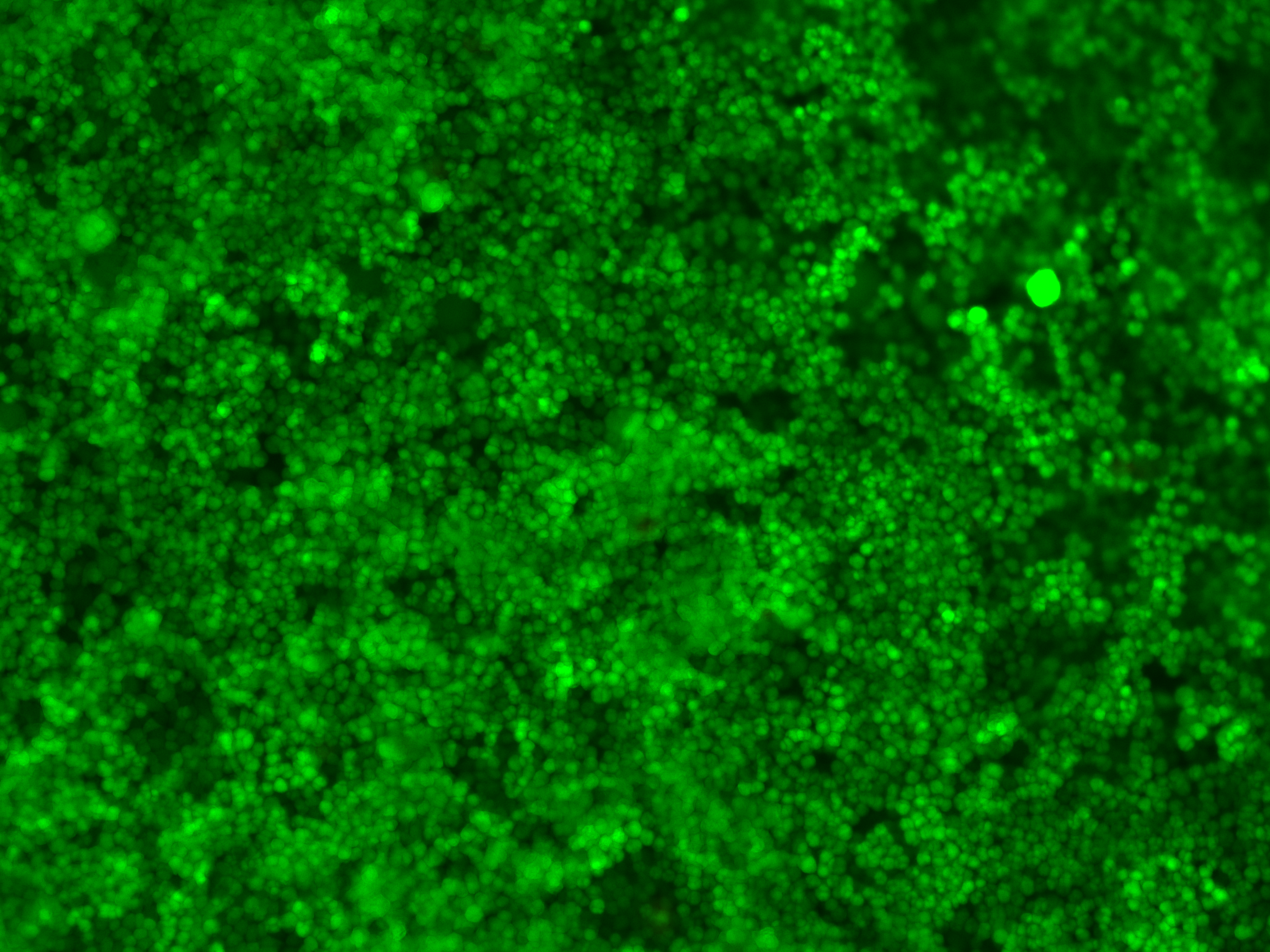

Supplement: Supplementary file 25 — Source Data [file 41467_2025_60928_MOESM25_ESM.zip › Source File/Fig. S37-38/Archive/staining/1023livedead-staining/ctrl/1.5.tiff]

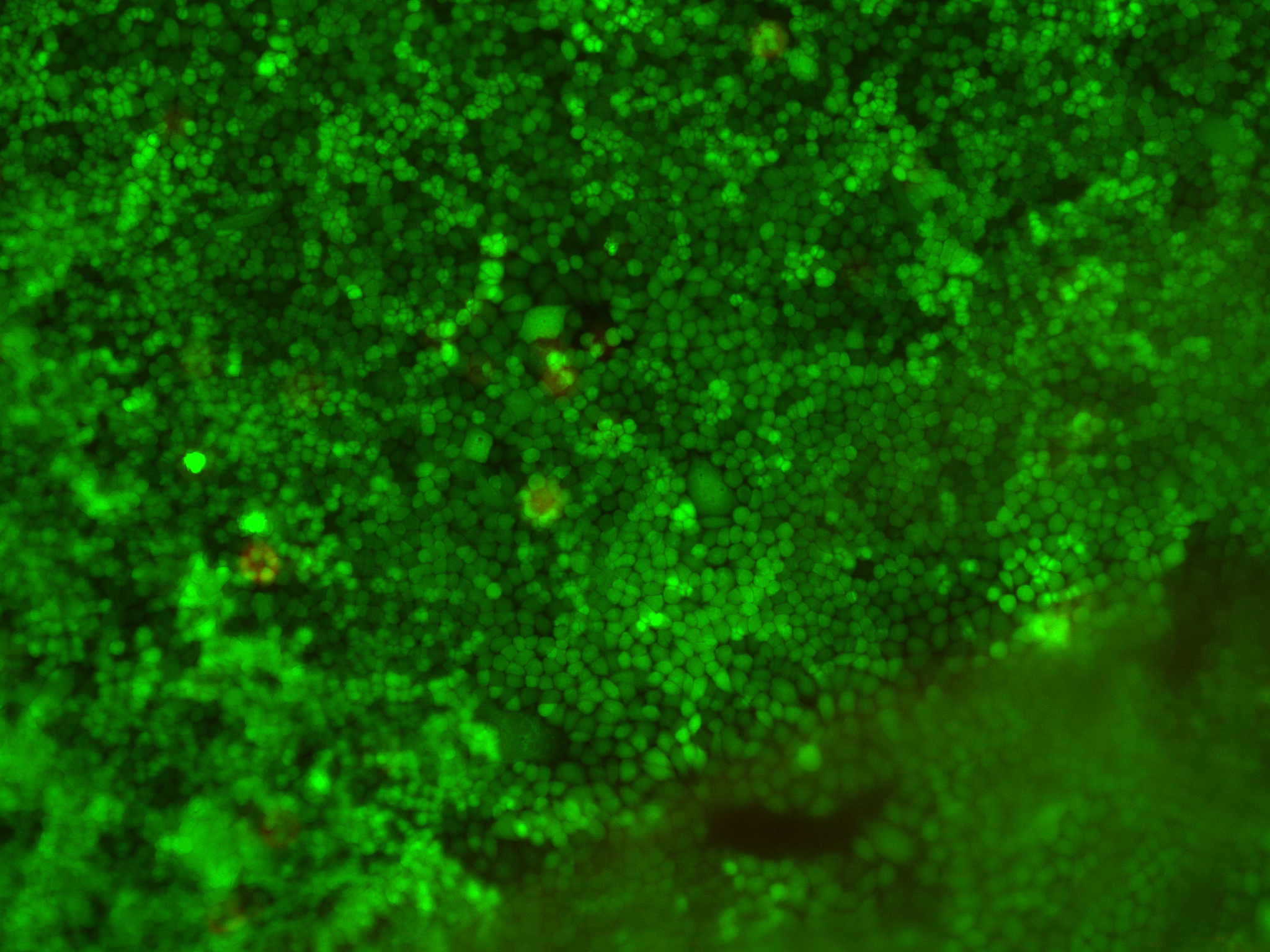

Supplement: Supplementary file 25 — Source Data [file 41467_2025_60928_MOESM25_ESM.zip › Source File/Fig. S37-38/Archive/staining/1023livedead-staining/ctrl/1.tiff]

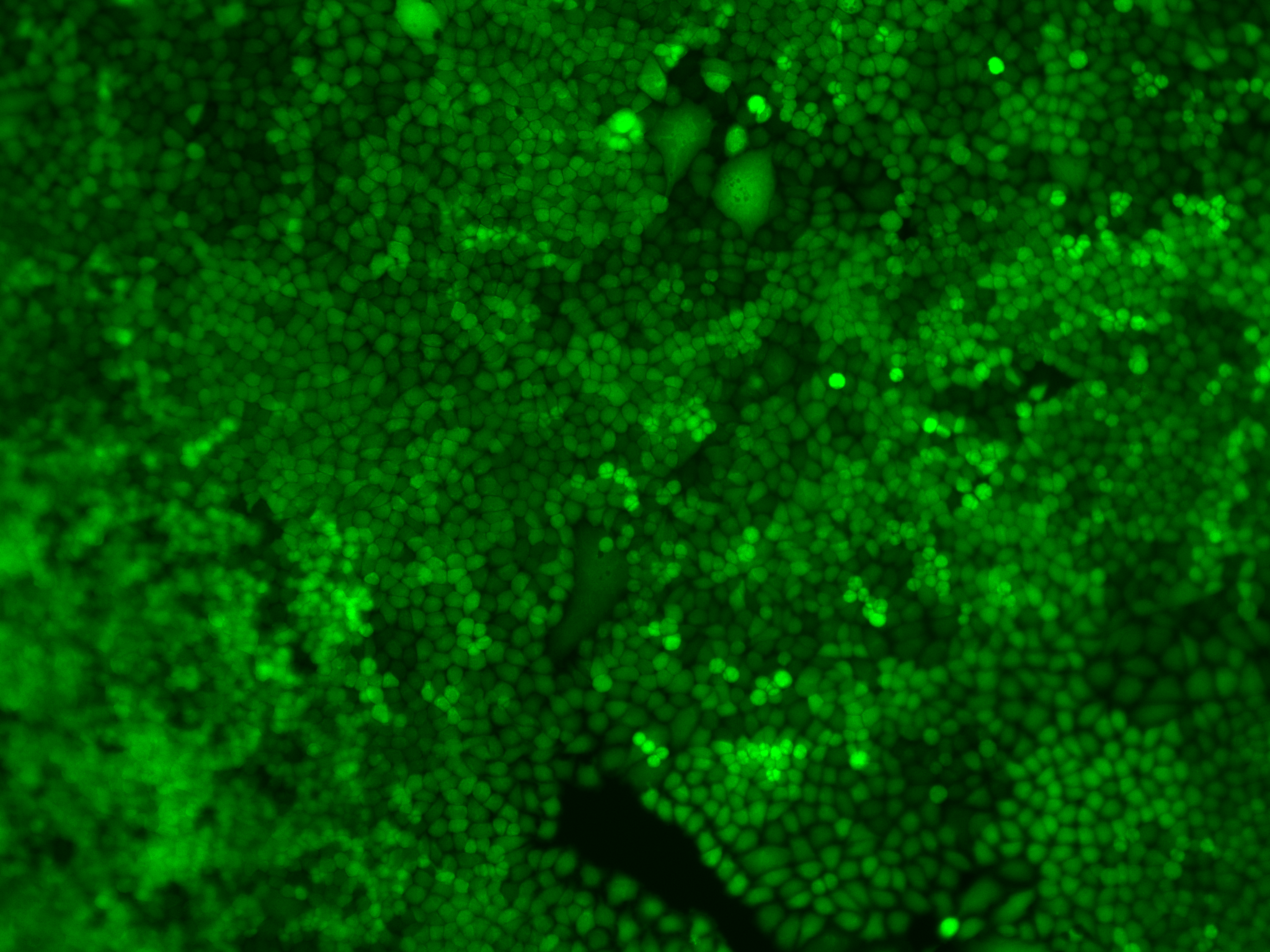

Supplement: Supplementary file 25 — Source Data [file 41467_2025_60928_MOESM25_ESM.zip › Source File/Fig. S37-38/Archive/staining/1023livedead-staining/ctrl/2.1.tiff]

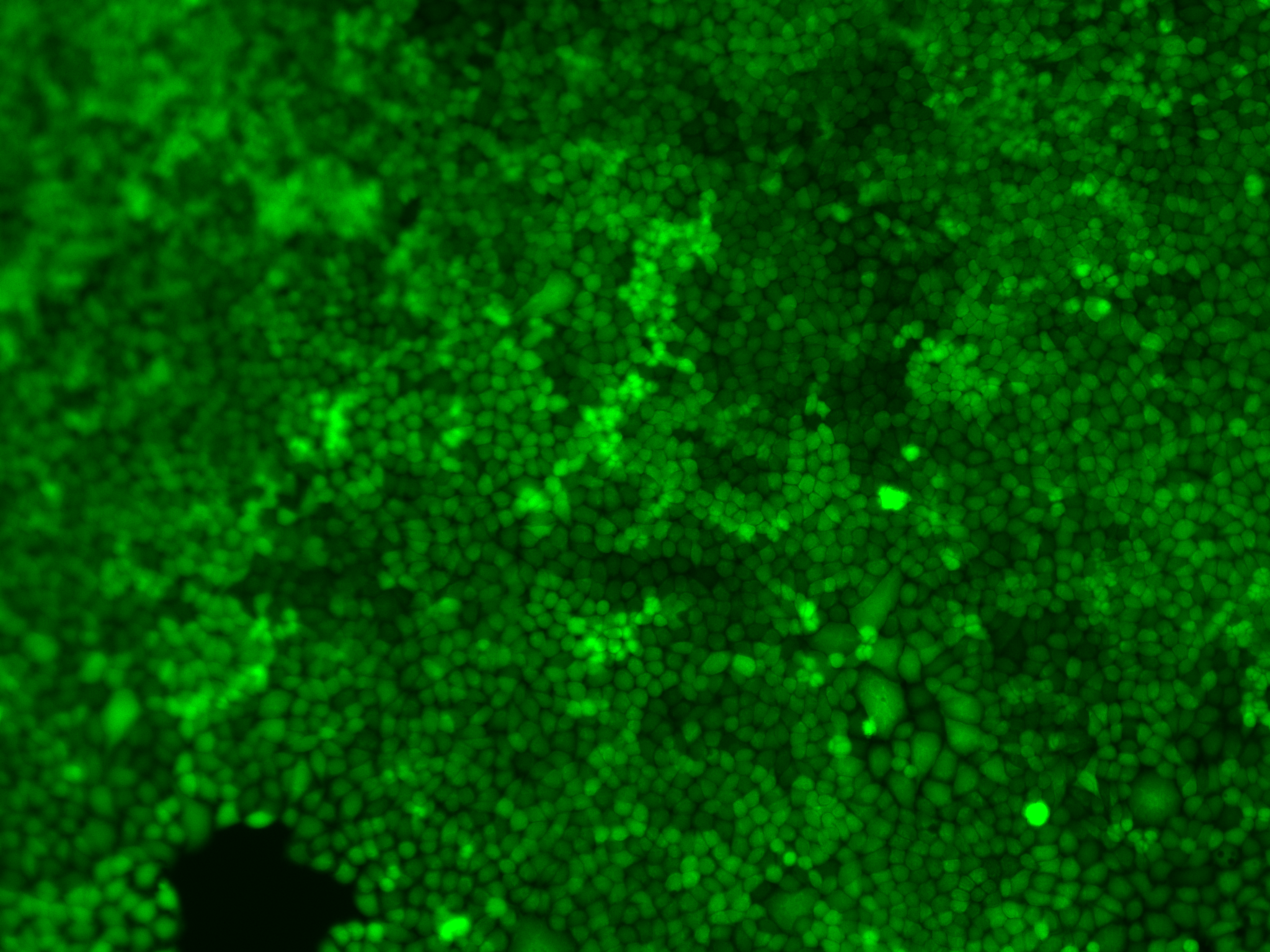

Supplement: Supplementary file 25 — Source Data [file 41467_2025_60928_MOESM25_ESM.zip › Source File/Fig. S37-38/Archive/staining/1023livedead-staining/ctrl/2.2.tiff]

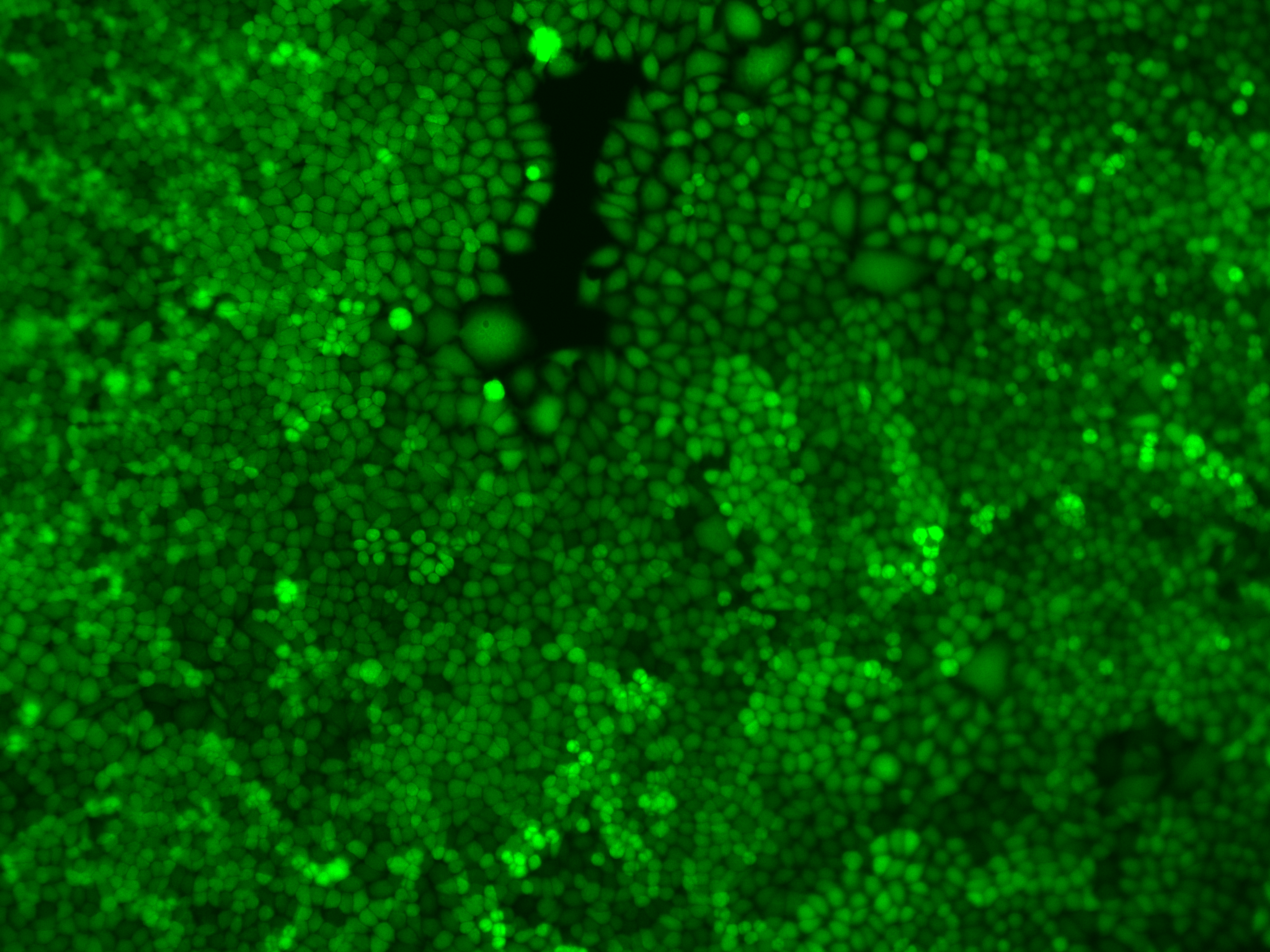

Supplement: Supplementary file 25 — Source Data [file 41467_2025_60928_MOESM25_ESM.zip › Source File/Fig. S37-38/Archive/staining/1023livedead-staining/ctrl/2.3.tiff]

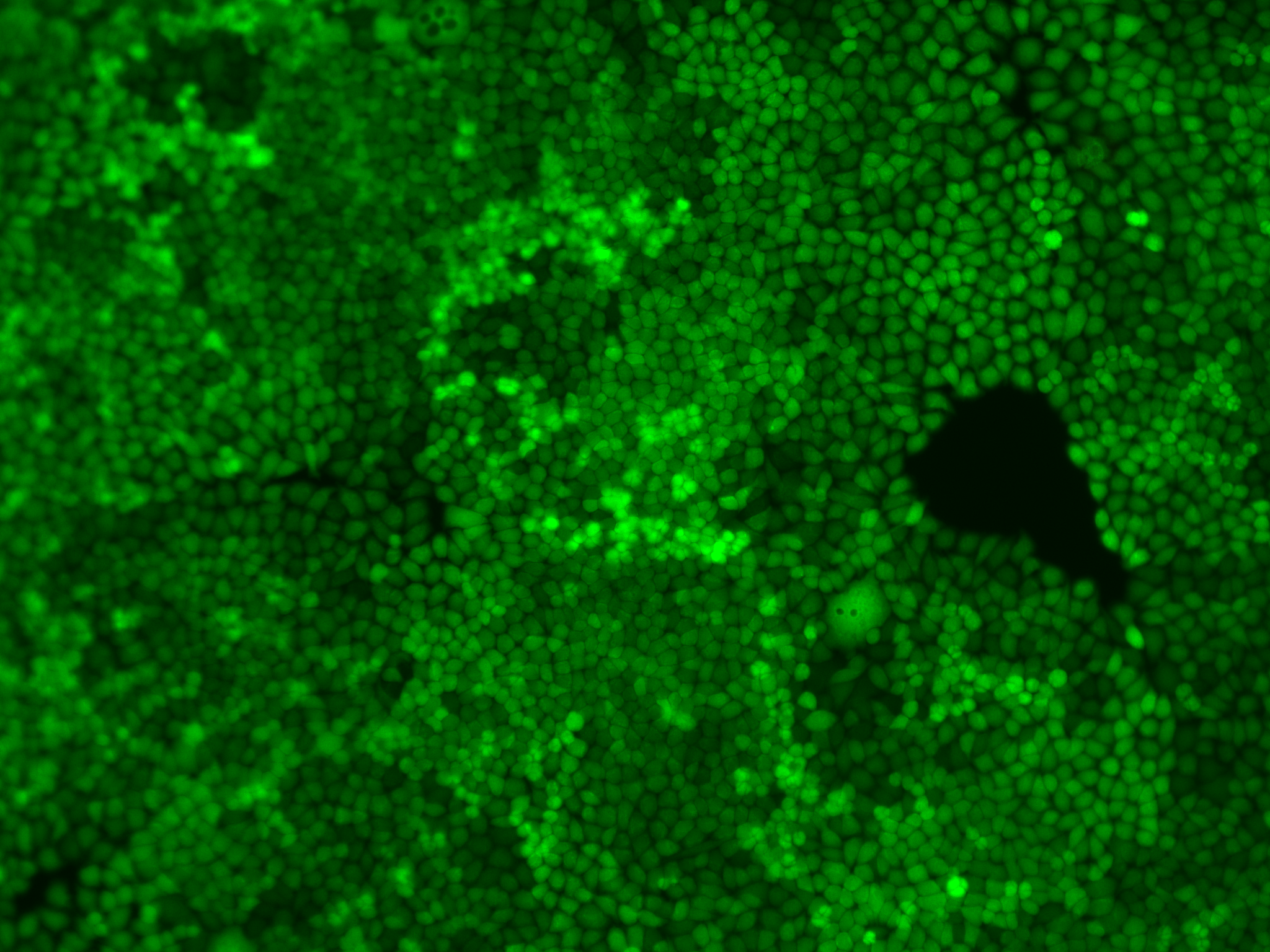

Supplement: Supplementary file 25 — Source Data [file 41467_2025_60928_MOESM25_ESM.zip › Source File/Fig. S37-38/Archive/staining/1023livedead-staining/ctrl/3.1.tiff]

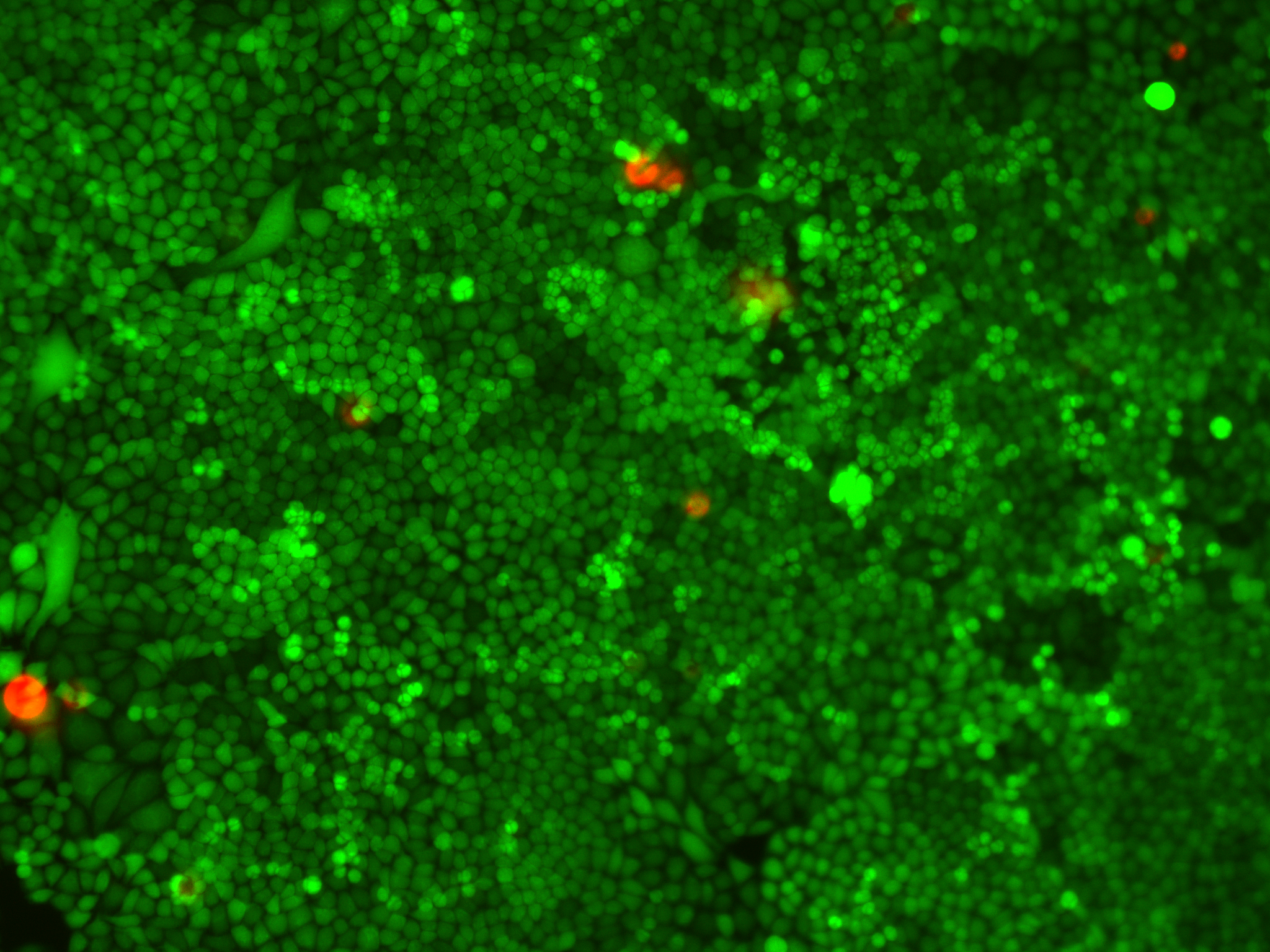

Supplement: Supplementary file 25 — Source Data [file 41467_2025_60928_MOESM25_ESM.zip › Source File/Fig. S37-38/Archive/staining/1023livedead-staining/ctrl/3.2.tiff]

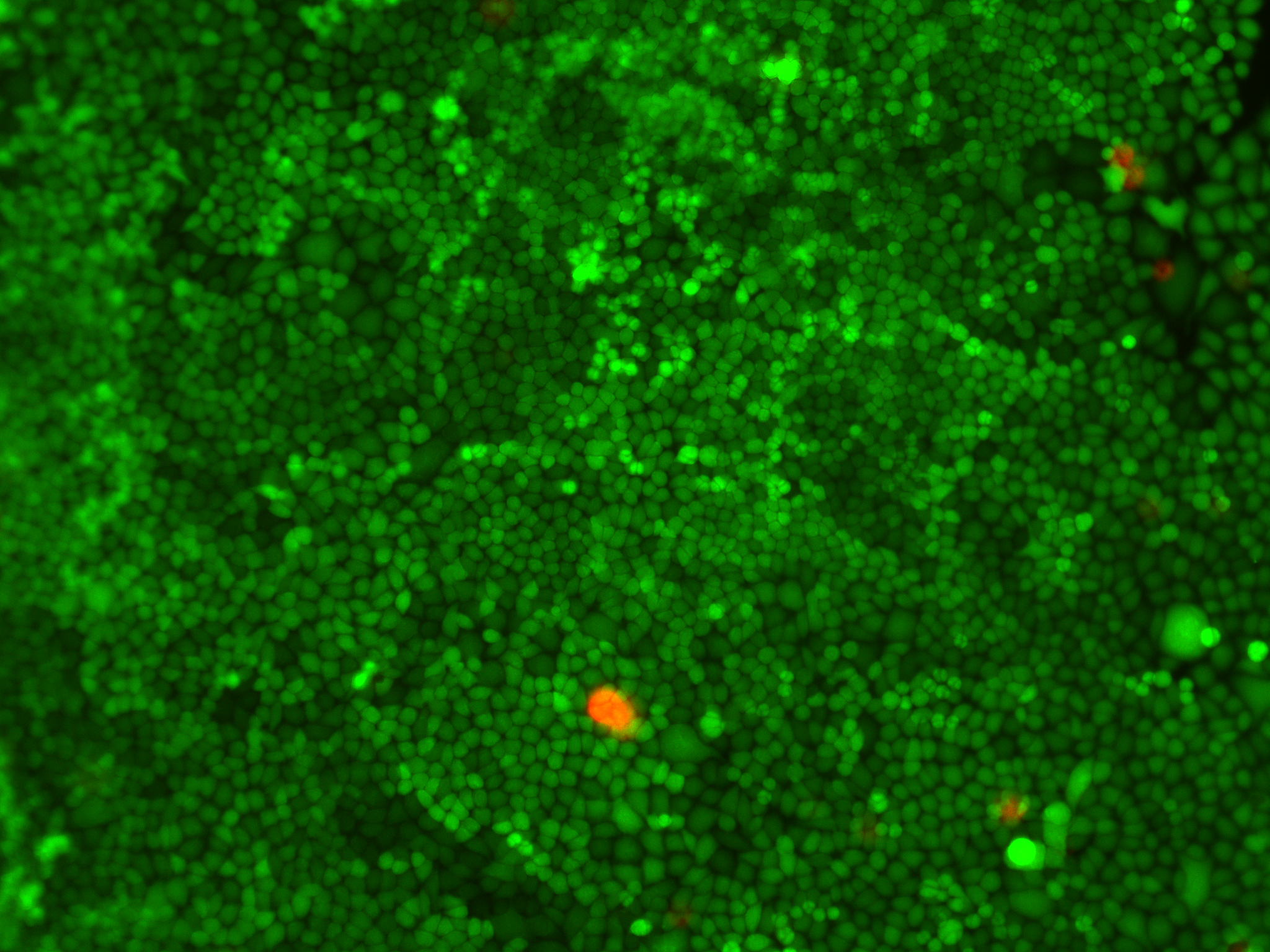

Supplement: Supplementary file 25 — Source Data [file 41467_2025_60928_MOESM25_ESM.zip › Source File/Fig. S37-38/Archive/staining/1023livedead-staining/ctrl/3.3.tiff]

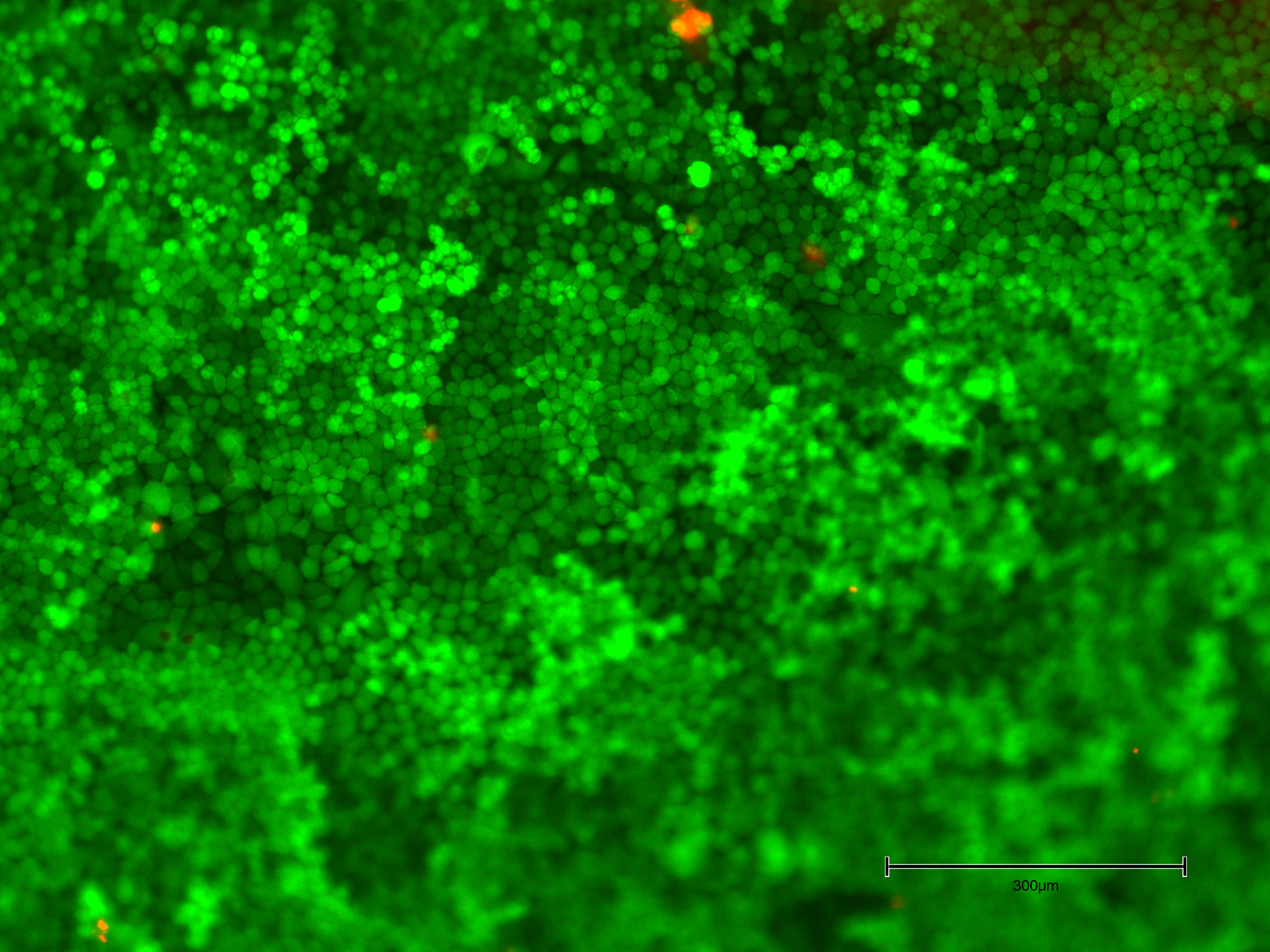

Supplement: Supplementary file 25 — Source Data [file 41467_2025_60928_MOESM25_ESM.zip › Source File/Fig. S37-38/Archive/staining/1023livedead-staining/Fe3O4/-3-green.tiff]

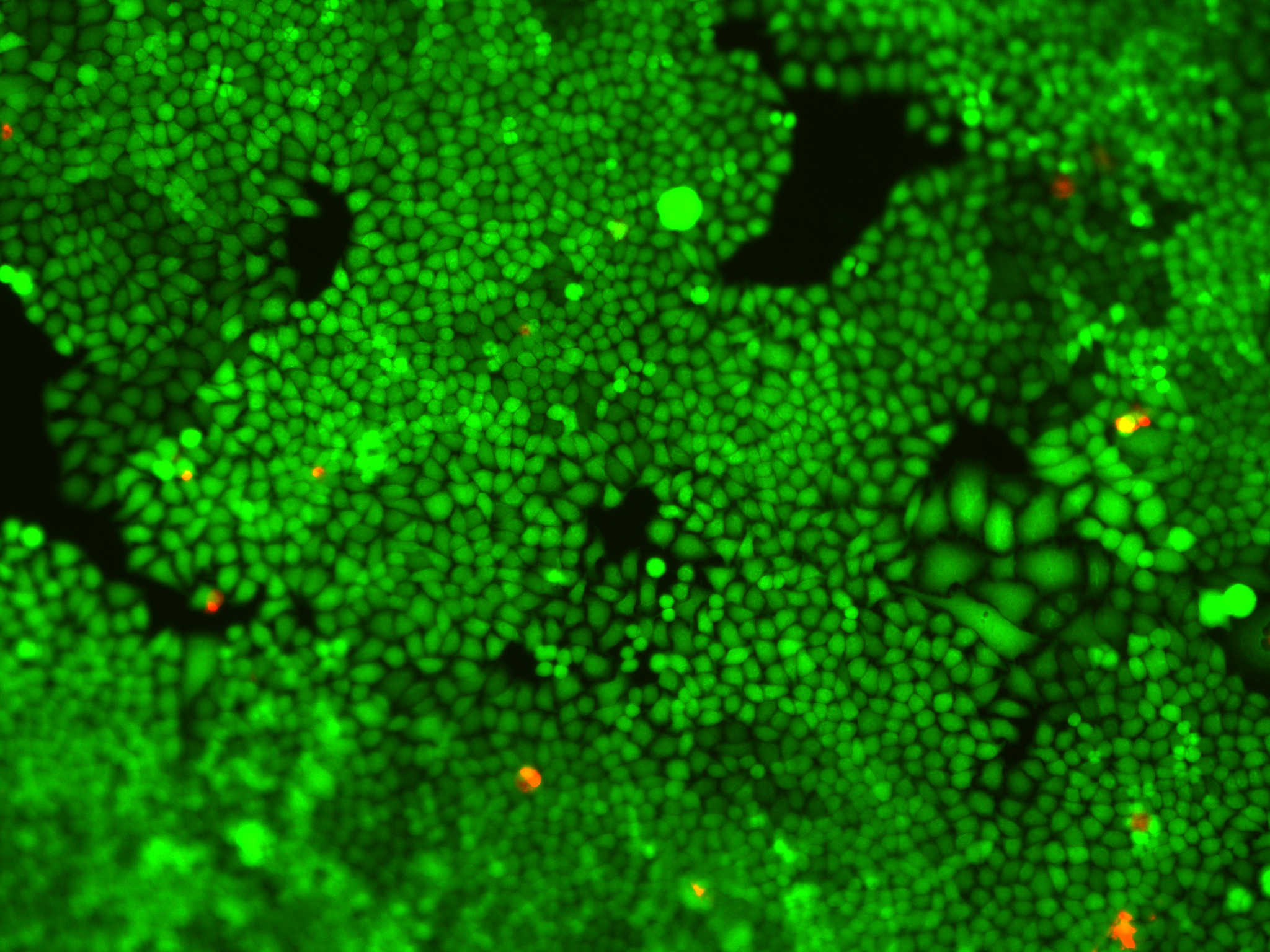

Supplement: Supplementary file 25 — Source Data [file 41467_2025_60928_MOESM25_ESM.zip › Source File/Fig. S37-38/Archive/staining/1023livedead-staining/Fe3O4/1.tiff]

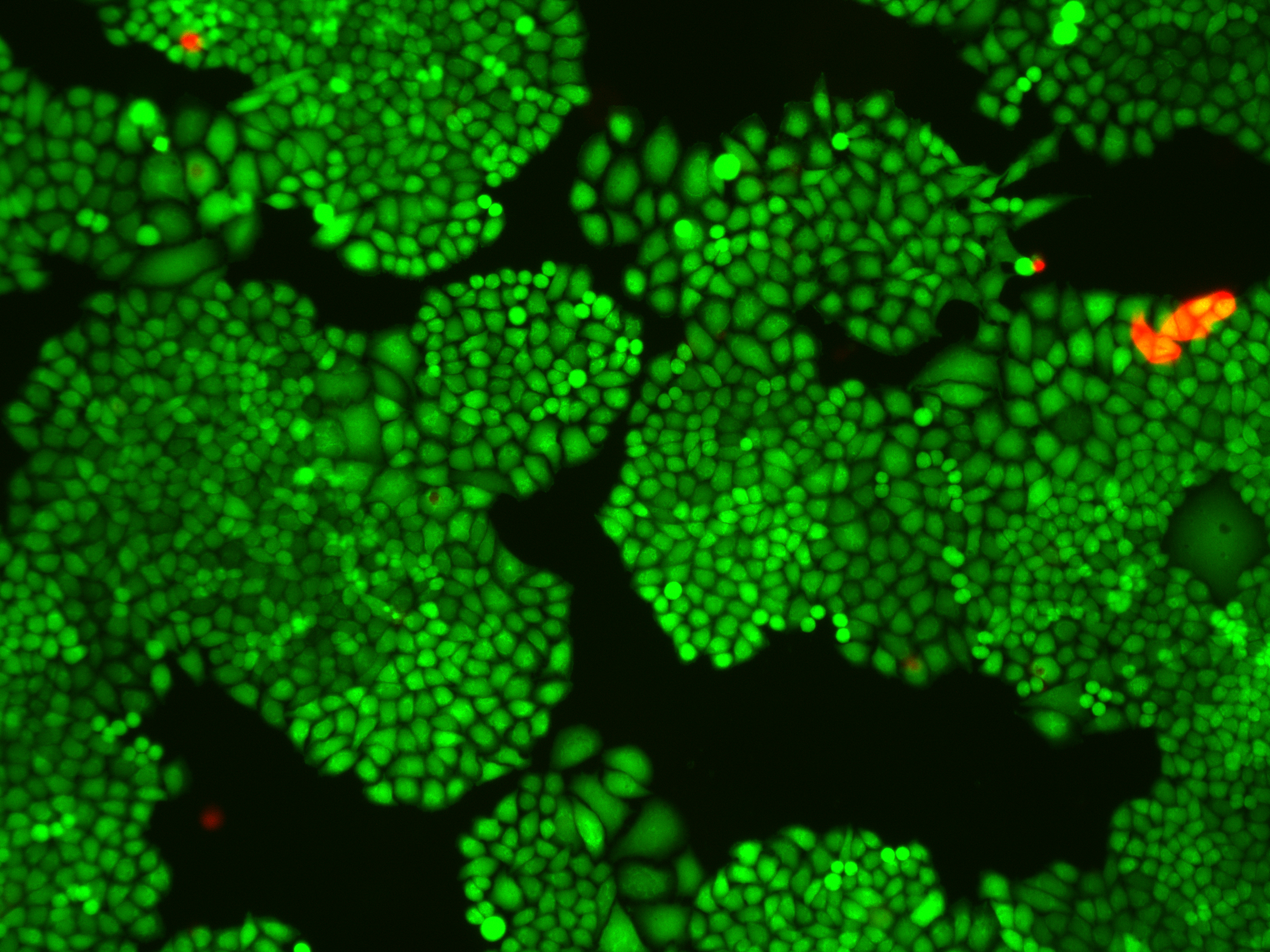

Supplement: Supplementary file 25 — Source Data [file 41467_2025_60928_MOESM25_ESM.zip › Source File/Fig. S37-38/Archive/staining/1023livedead-staining/Fe3O4/2.1.tiff]

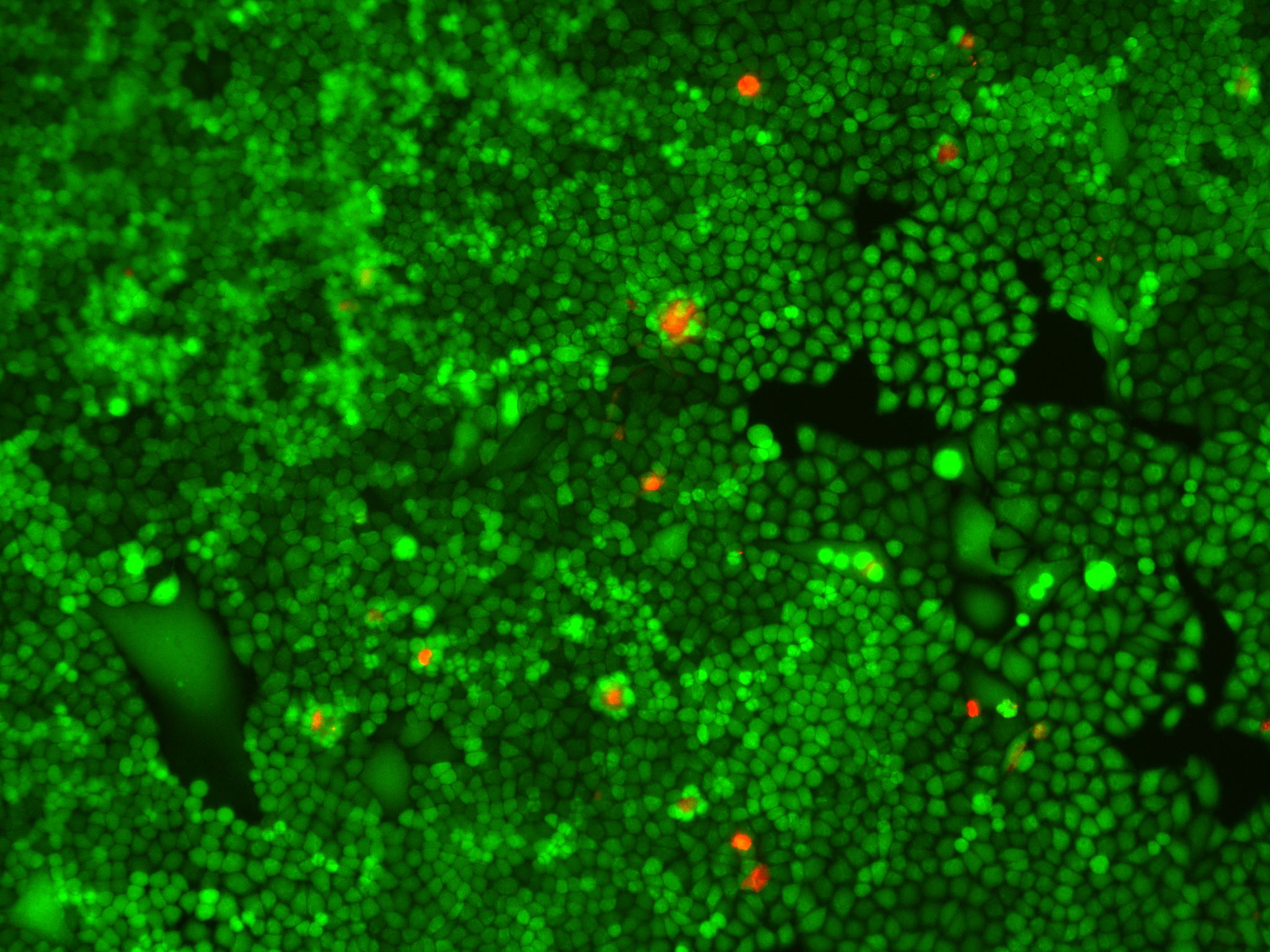

Supplement: Supplementary file 25 — Source Data [file 41467_2025_60928_MOESM25_ESM.zip › Source File/Fig. S37-38/Archive/staining/1023livedead-staining/Fe3O4/2.2.tiff]

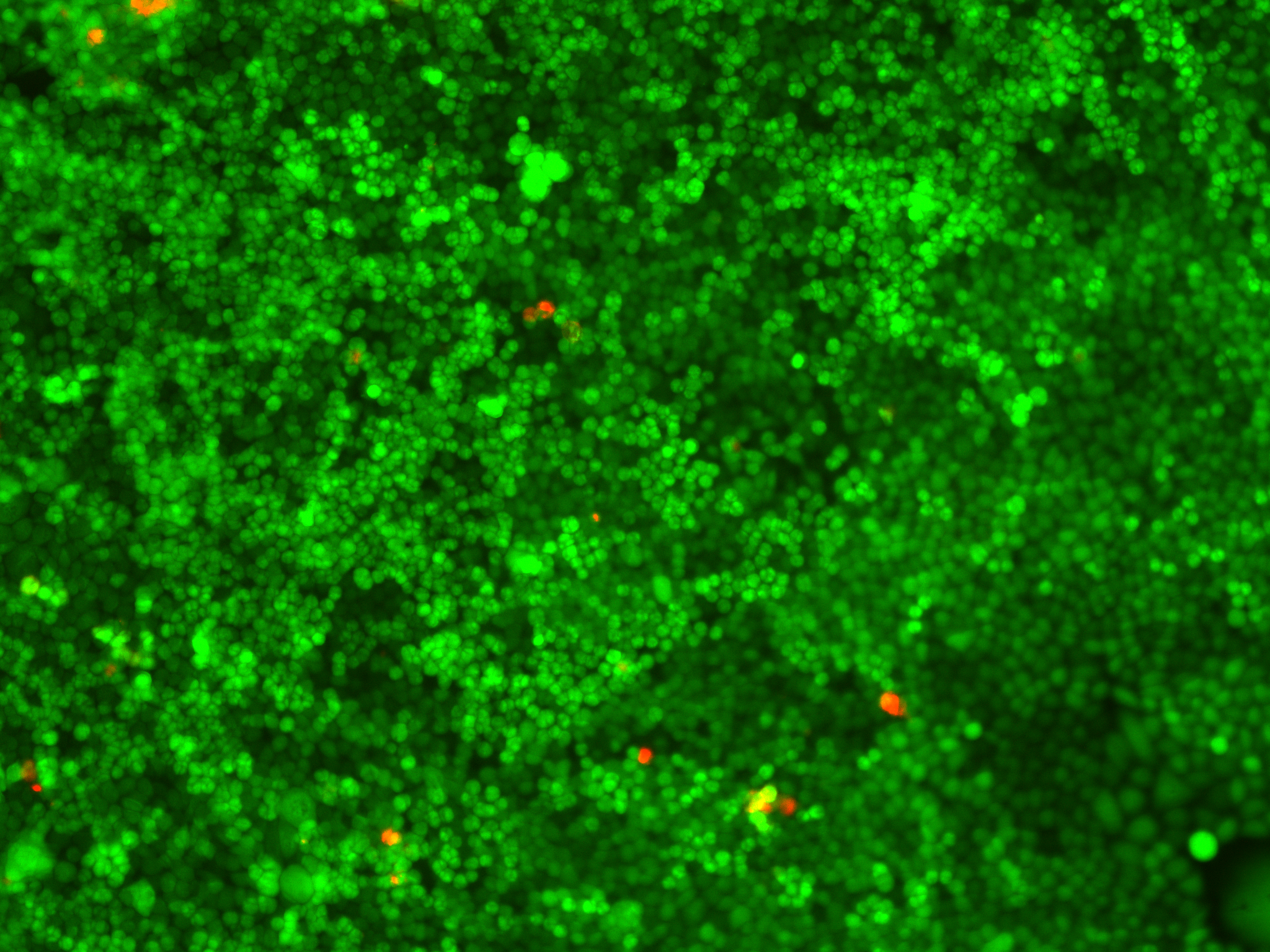

Supplement: Supplementary file 25 — Source Data [file 41467_2025_60928_MOESM25_ESM.zip › Source File/Fig. S37-38/Archive/staining/1023livedead-staining/Fe3O4/2.3.tiff]

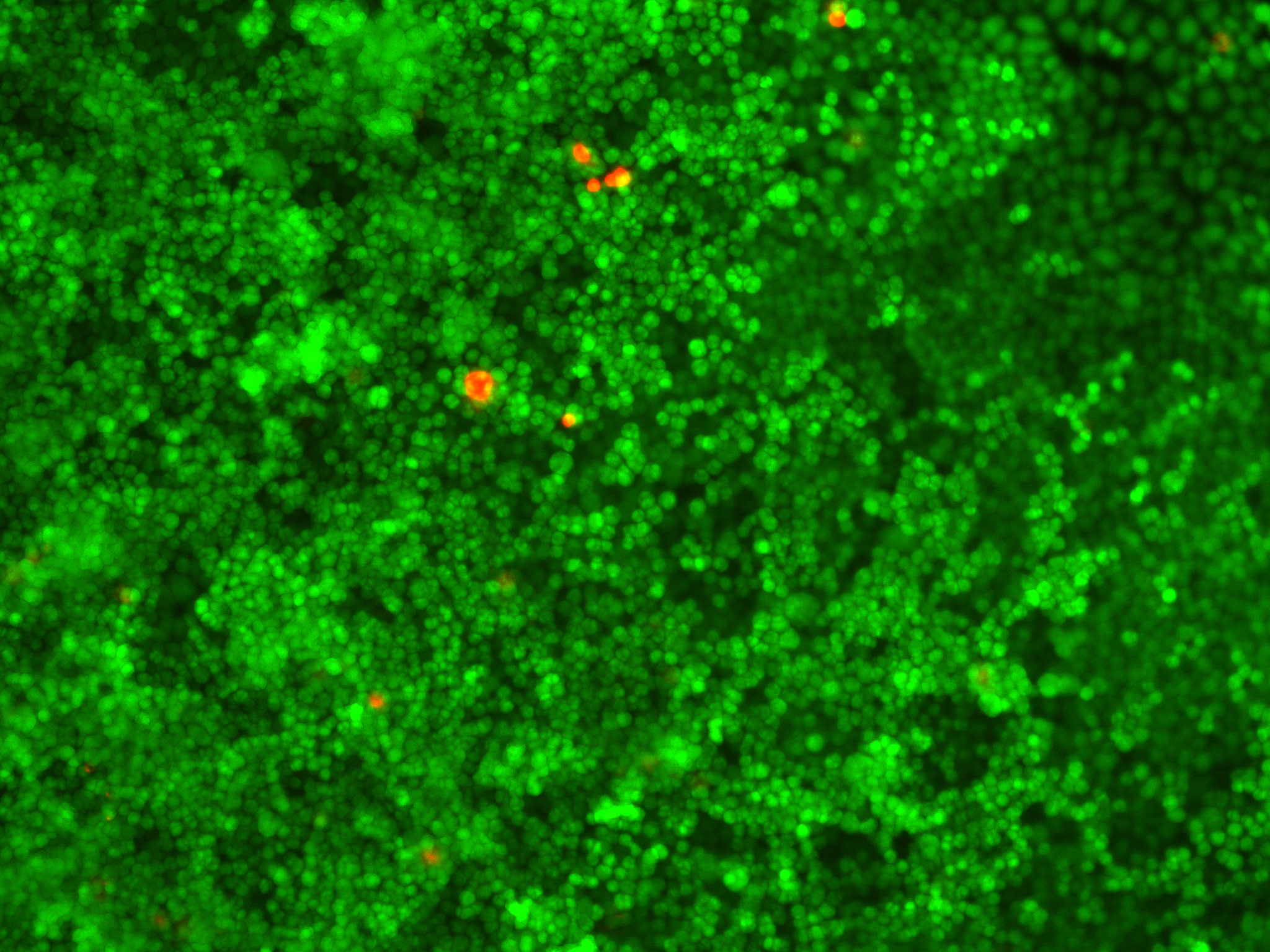

Supplement: Supplementary file 25 — Source Data [file 41467_2025_60928_MOESM25_ESM.zip › Source File/Fig. S37-38/Archive/staining/1023livedead-staining/Fe3O4/2.4.tiff]

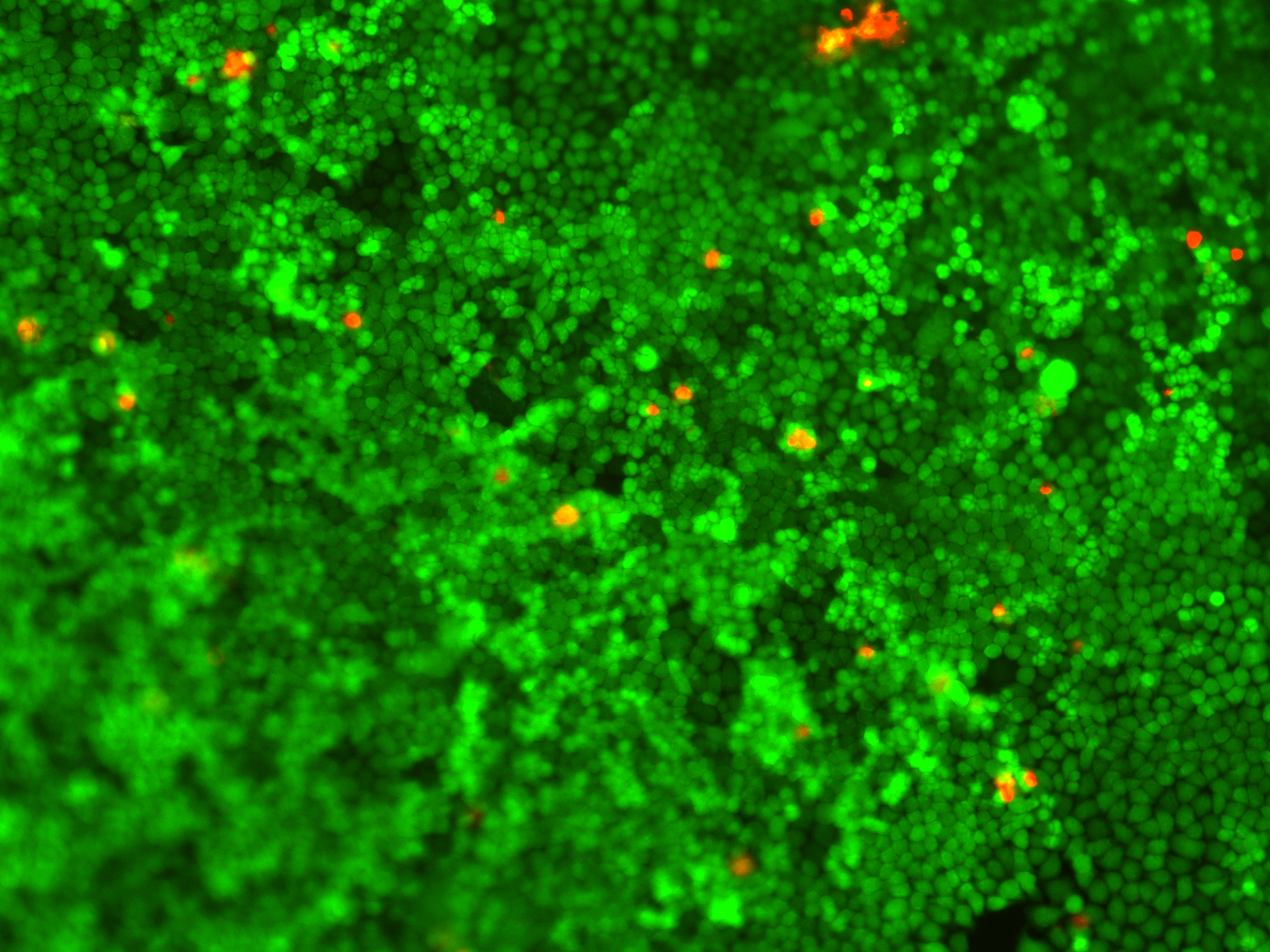

Supplement: Supplementary file 25 — Source Data [file 41467_2025_60928_MOESM25_ESM.zip › Source File/Fig. S37-38/Archive/staining/1023livedead-staining/Fe3O4/2.tiff]

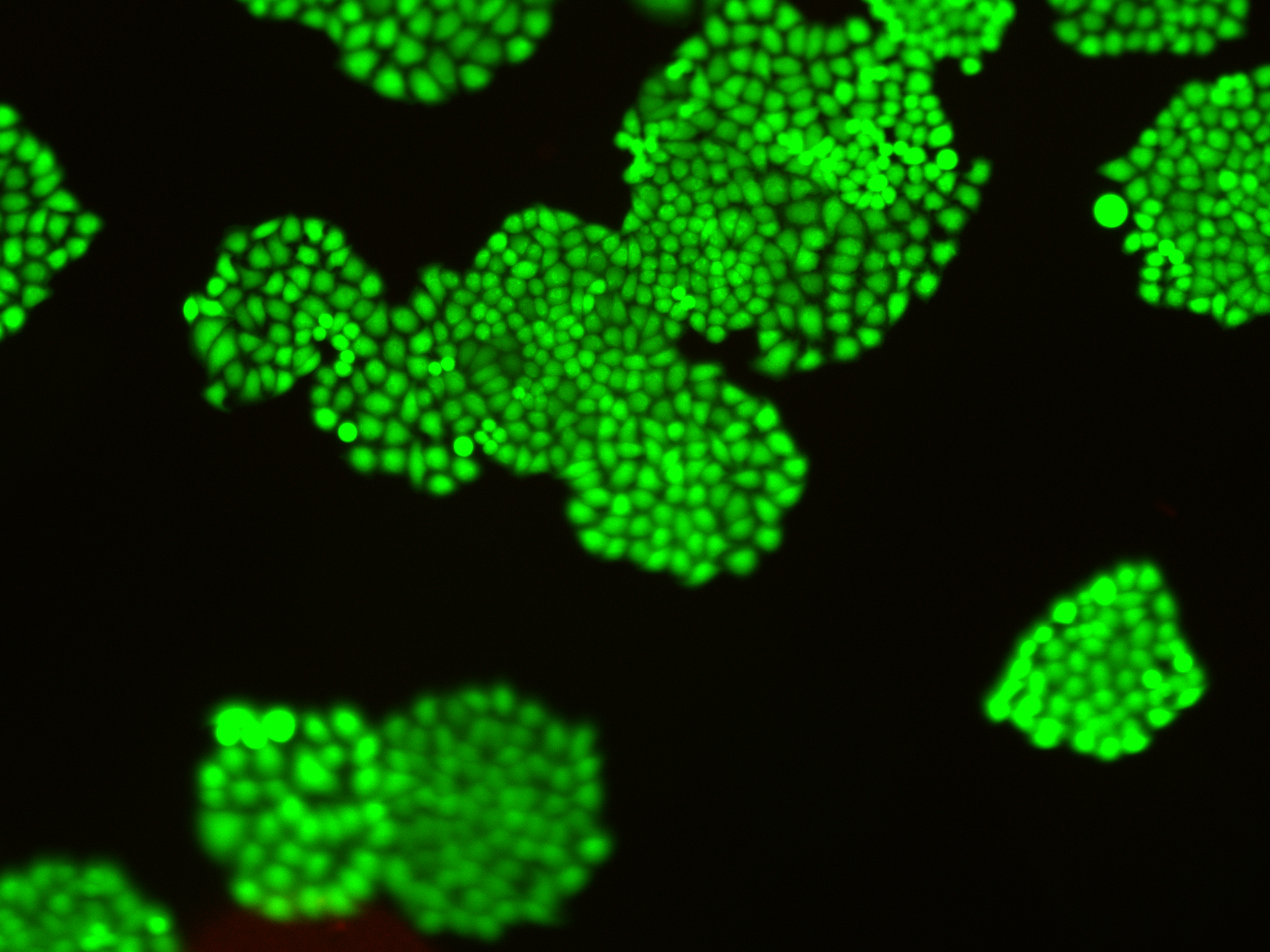

Supplement: Supplementary file 25 — Source Data [file 41467_2025_60928_MOESM25_ESM.zip › Source File/Fig. S37-38/Archive/staining/1023livedead-staining/Fe3O4/3.1.tiff]

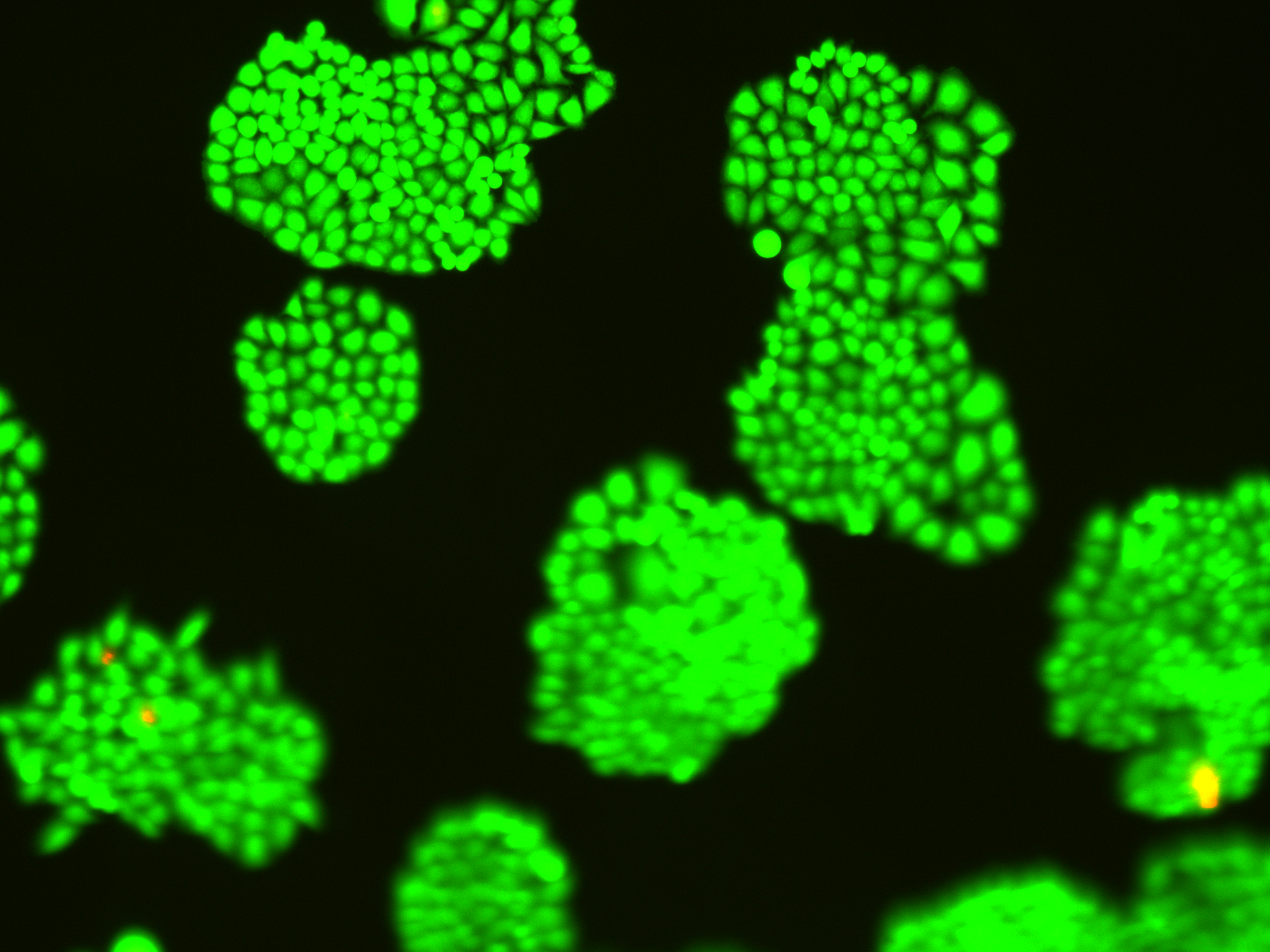

Supplement: Supplementary file 25 — Source Data [file 41467_2025_60928_MOESM25_ESM.zip › Source File/Fig. S37-38/Archive/staining/1023livedead-staining/Fe3O4/3.2.tiff]

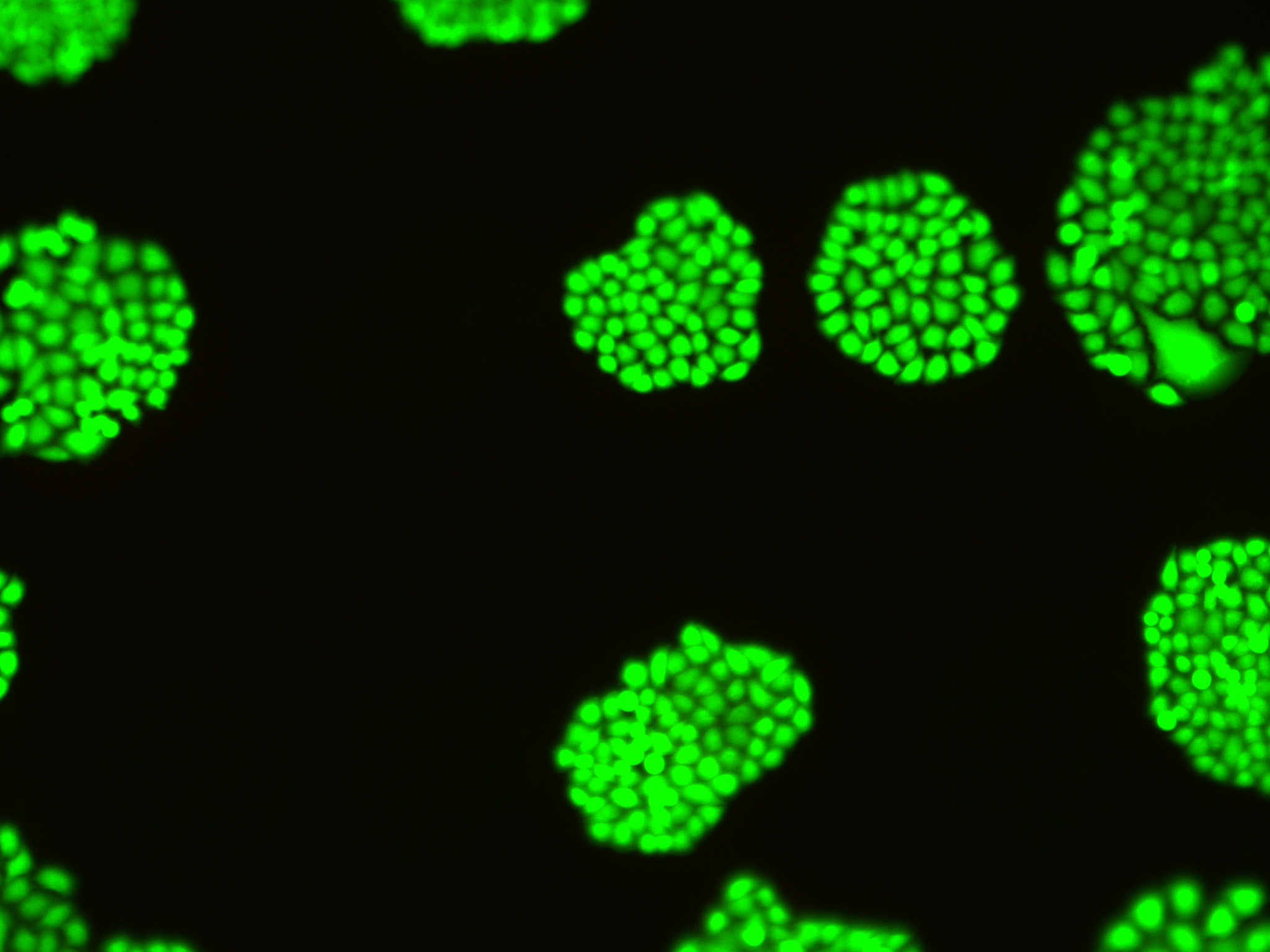

Supplement: Supplementary file 25 — Source Data [file 41467_2025_60928_MOESM25_ESM.zip › Source File/Fig. S37-38/Archive/staining/1023livedead-staining/Fe3O4/3.3.tiff]

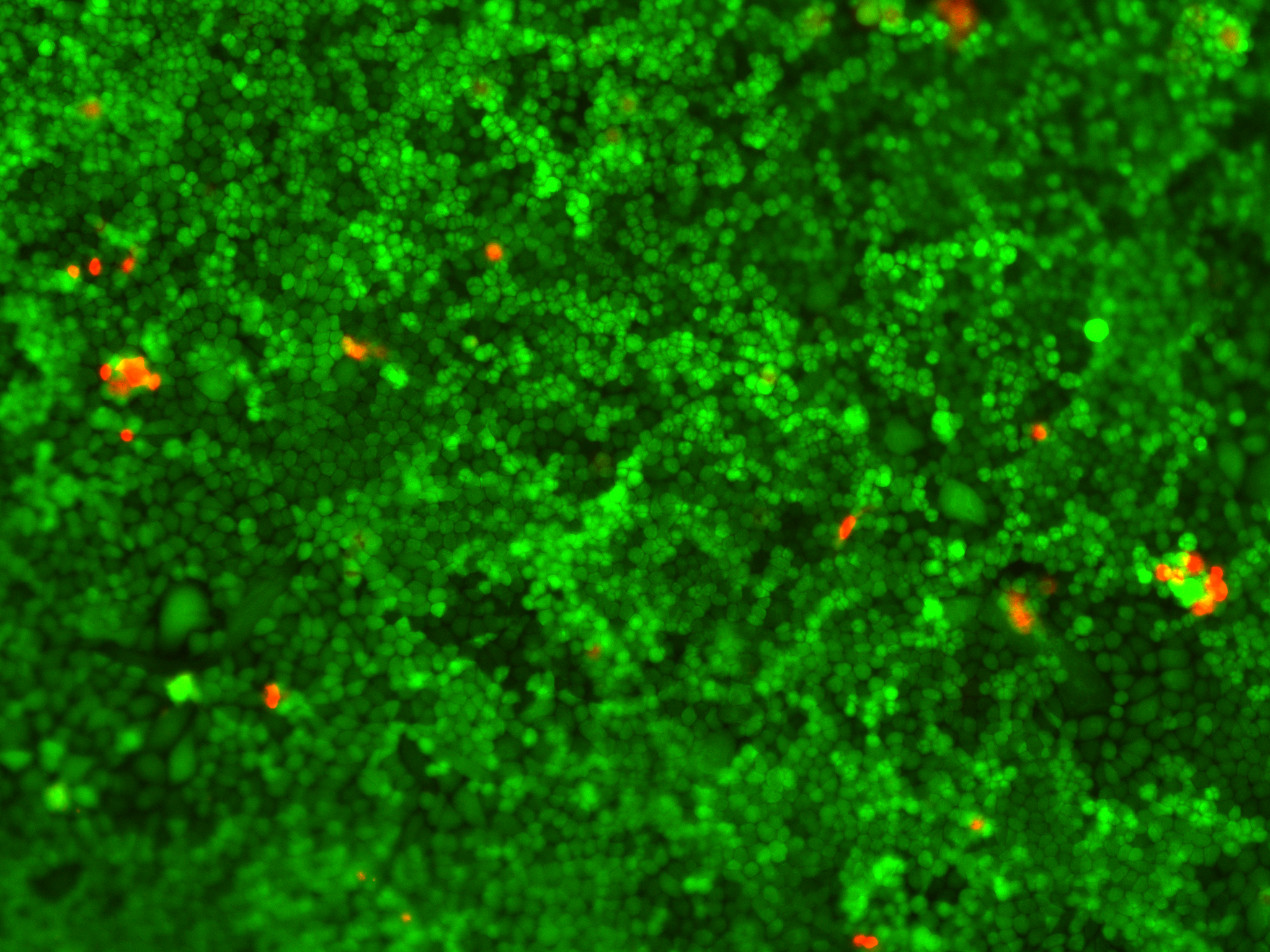

Supplement: Supplementary file 25 — Source Data [file 41467_2025_60928_MOESM25_ESM.zip › Source File/Fig. S37-38/Archive/staining/1023livedead-staining/Fe3O4/3.tiff]

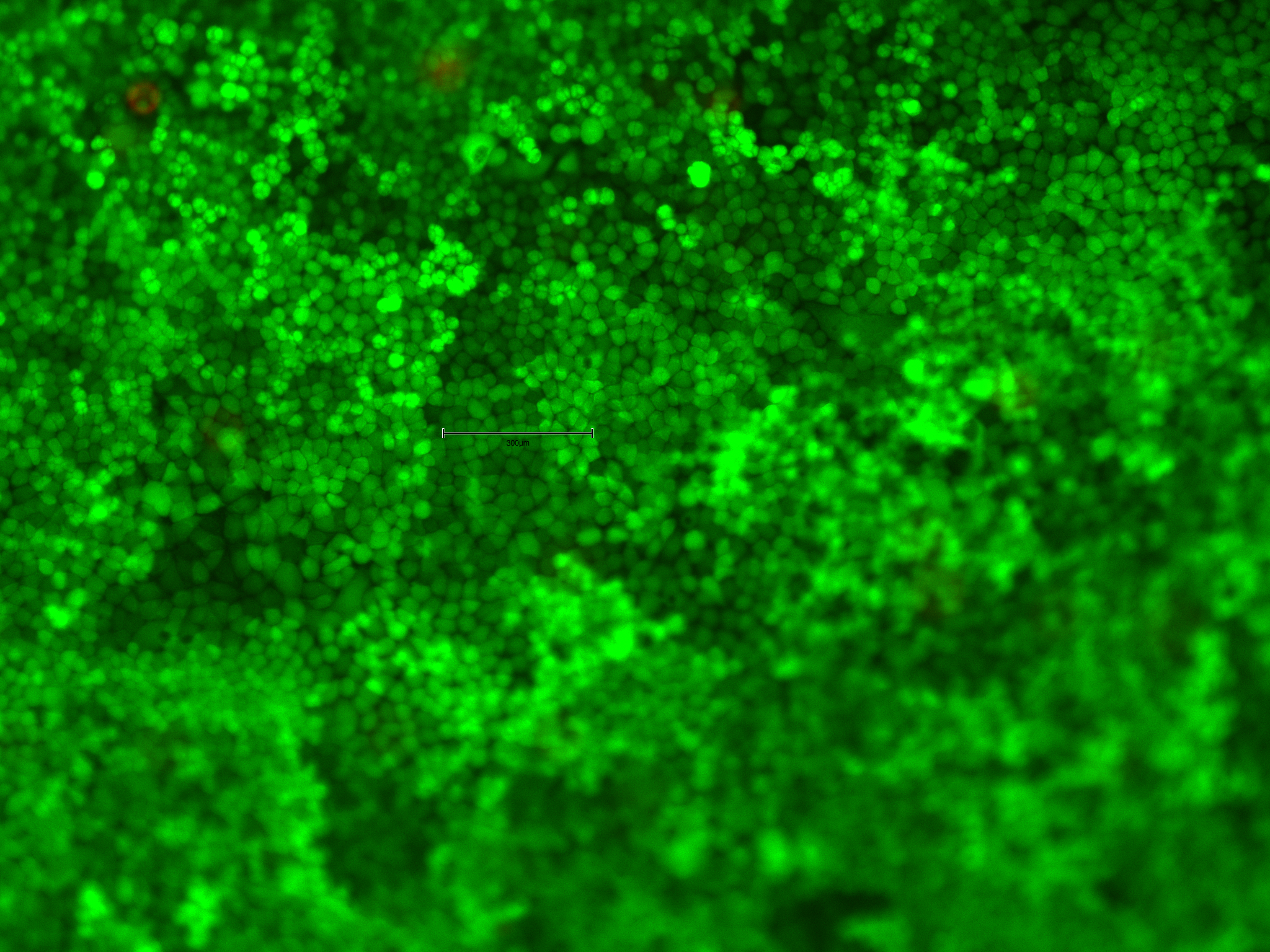

Supplement: Supplementary file 25 — Source Data [file 41467_2025_60928_MOESM25_ESM.zip › Source File/Fig. S37-38/Archive/staining/1023livedead-staining/Fe3O4/4.1.tiff]

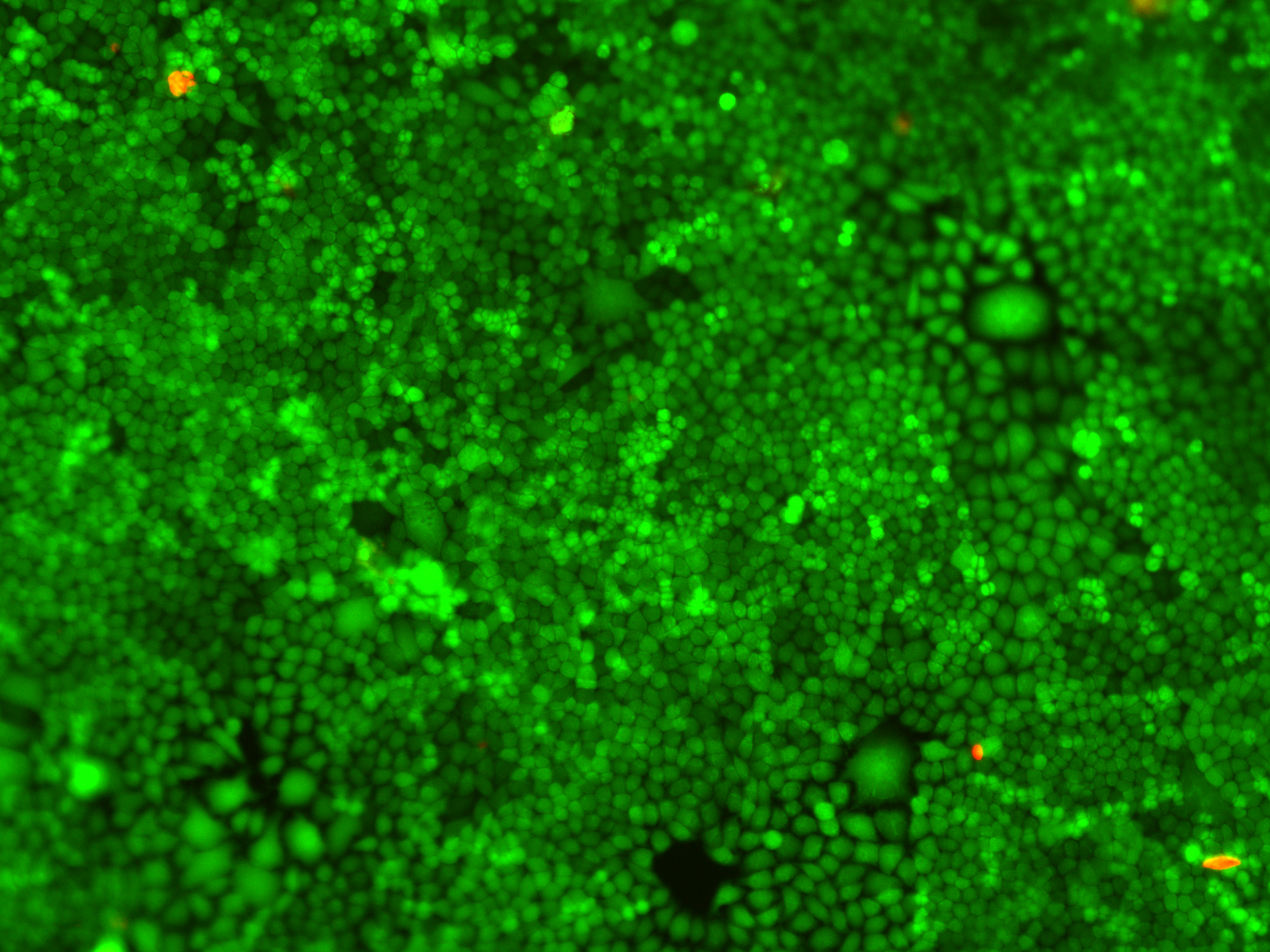

Supplement: Supplementary file 25 — Source Data [file 41467_2025_60928_MOESM25_ESM.zip › Source File/Fig. S37-38/Archive/staining/1023livedead-staining/Fe3O4/4.tiff]

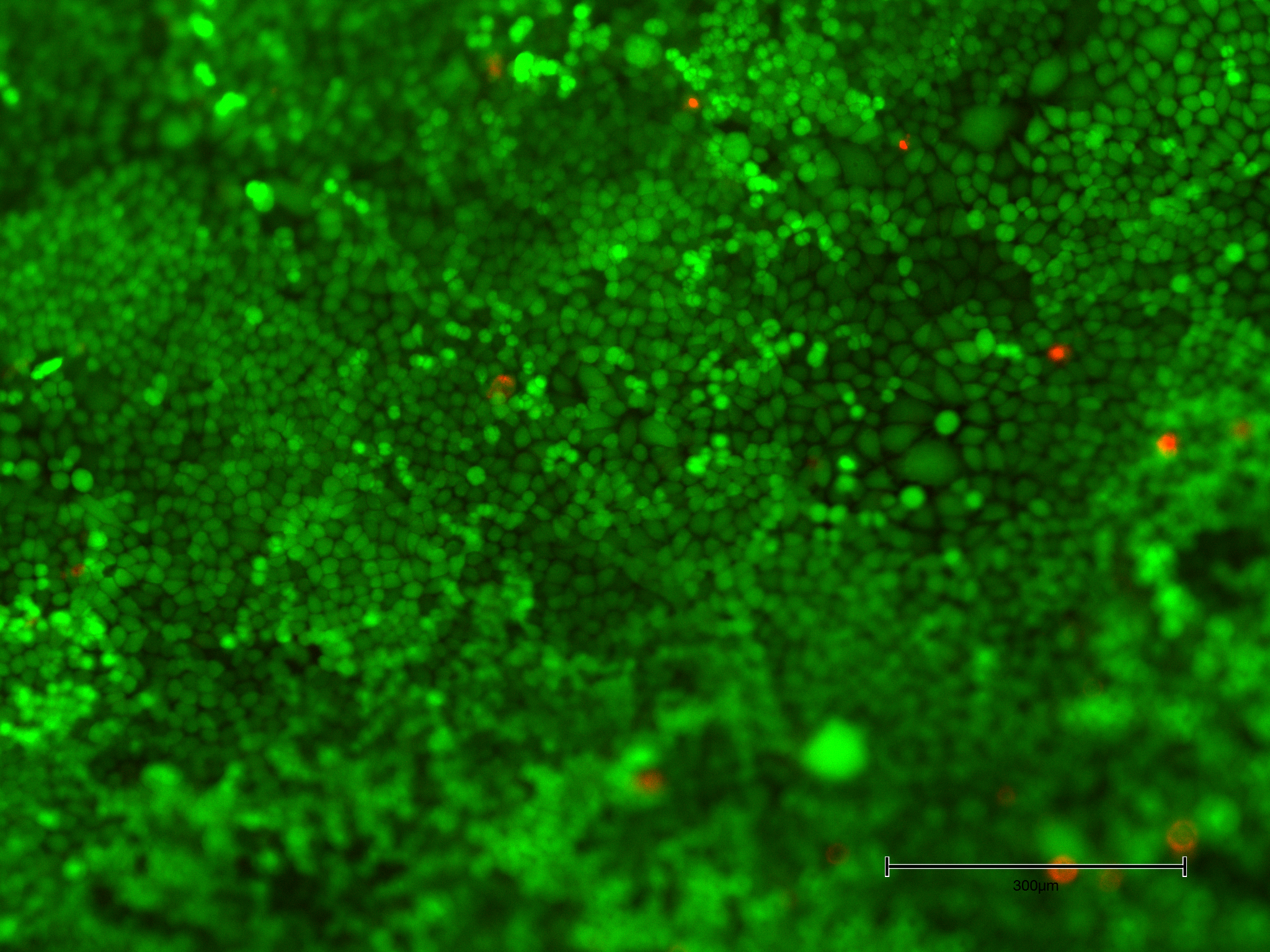

Supplement: Supplementary file 25 — Source Data [file 41467_2025_60928_MOESM25_ESM.zip › Source File/Fig. S37-38/Archive/staining/1023livedead-staining/Fe3O4/5 (1).tif]

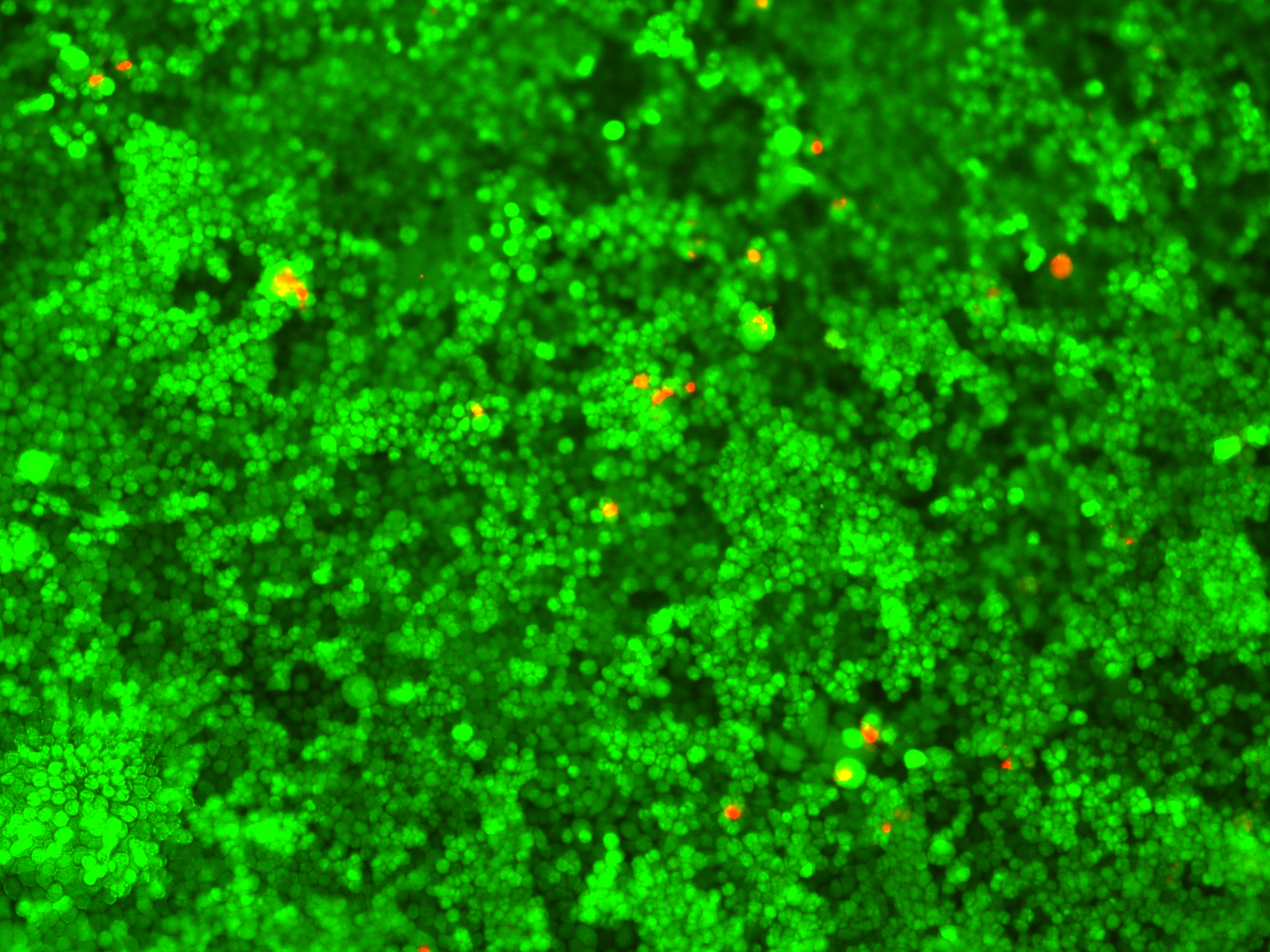

Supplement: Supplementary file 25 — Source Data [file 41467_2025_60928_MOESM25_ESM.zip › Source File/Fig. S37-38/Archive/staining/1023livedead-staining/NdFeB/1.1.tiff]

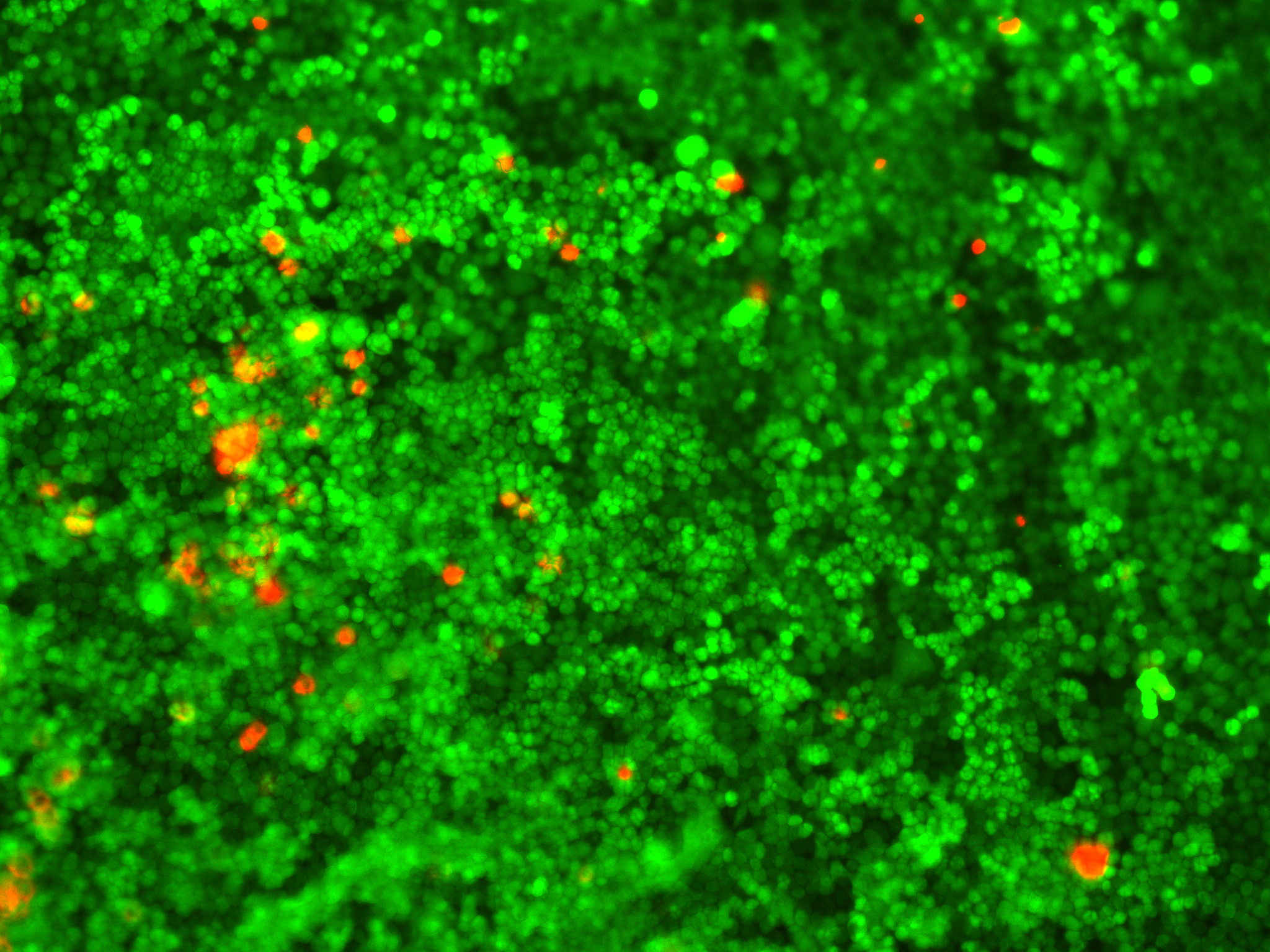

Supplement: Supplementary file 25 — Source Data [file 41467_2025_60928_MOESM25_ESM.zip › Source File/Fig. S37-38/Archive/staining/1023livedead-staining/NdFeB/1.2.tiff]

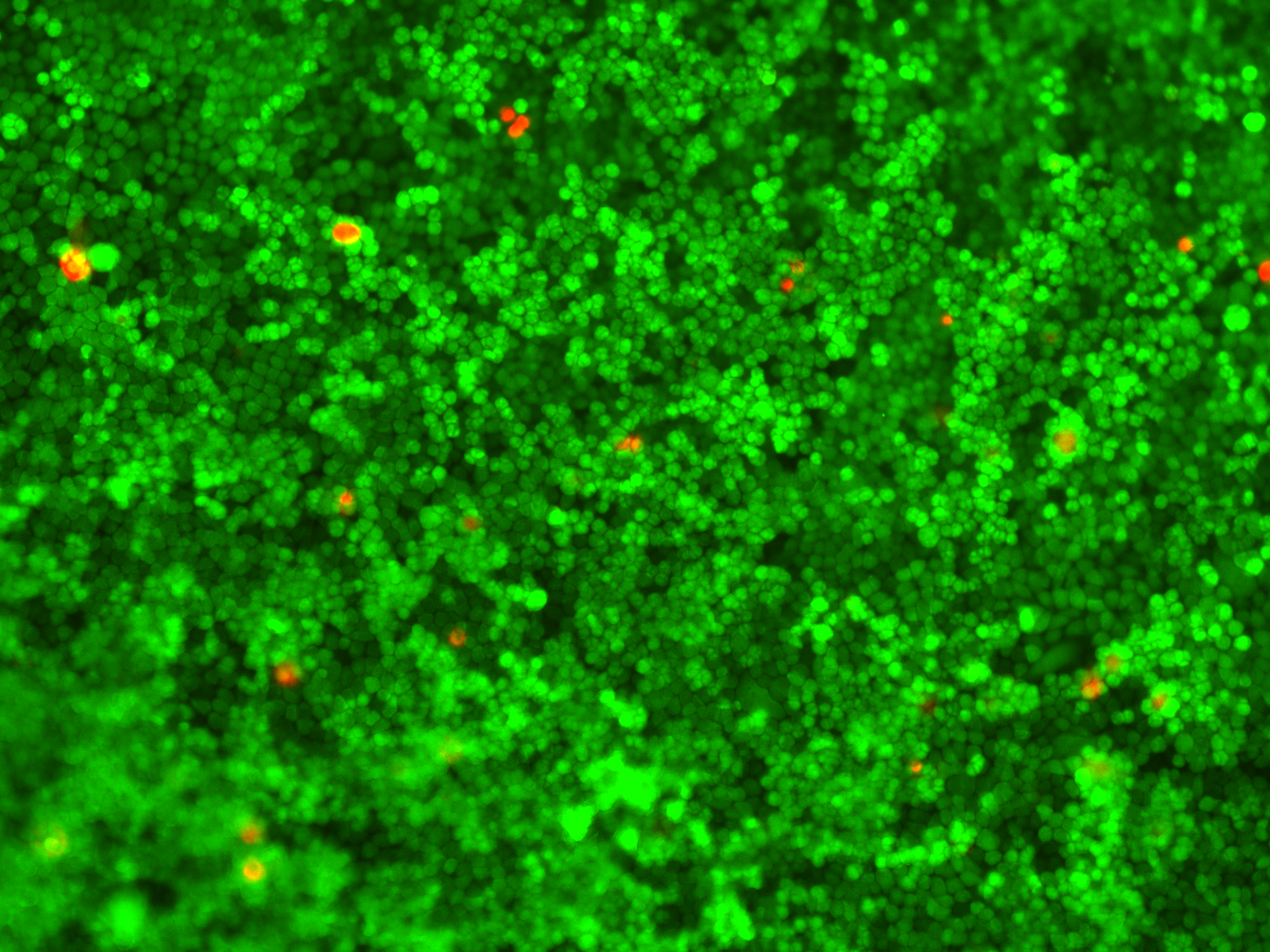

Supplement: Supplementary file 25 — Source Data [file 41467_2025_60928_MOESM25_ESM.zip › Source File/Fig. S37-38/Archive/staining/1023livedead-staining/NdFeB/1.3.tiff]

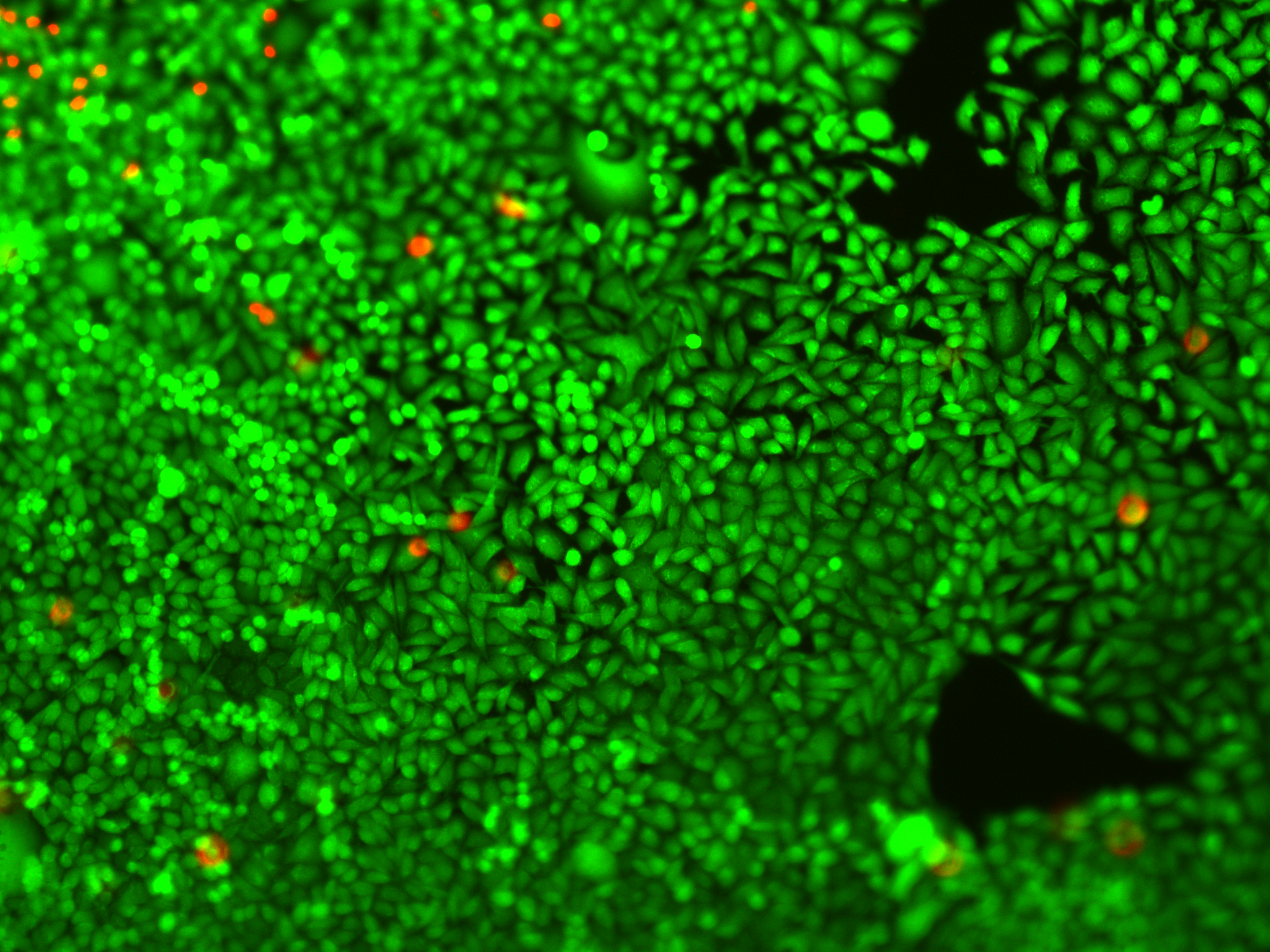

Supplement: Supplementary file 25 — Source Data [file 41467_2025_60928_MOESM25_ESM.zip › Source File/Fig. S37-38/Archive/staining/1023livedead-staining/NdFeB/2.1.tiff]

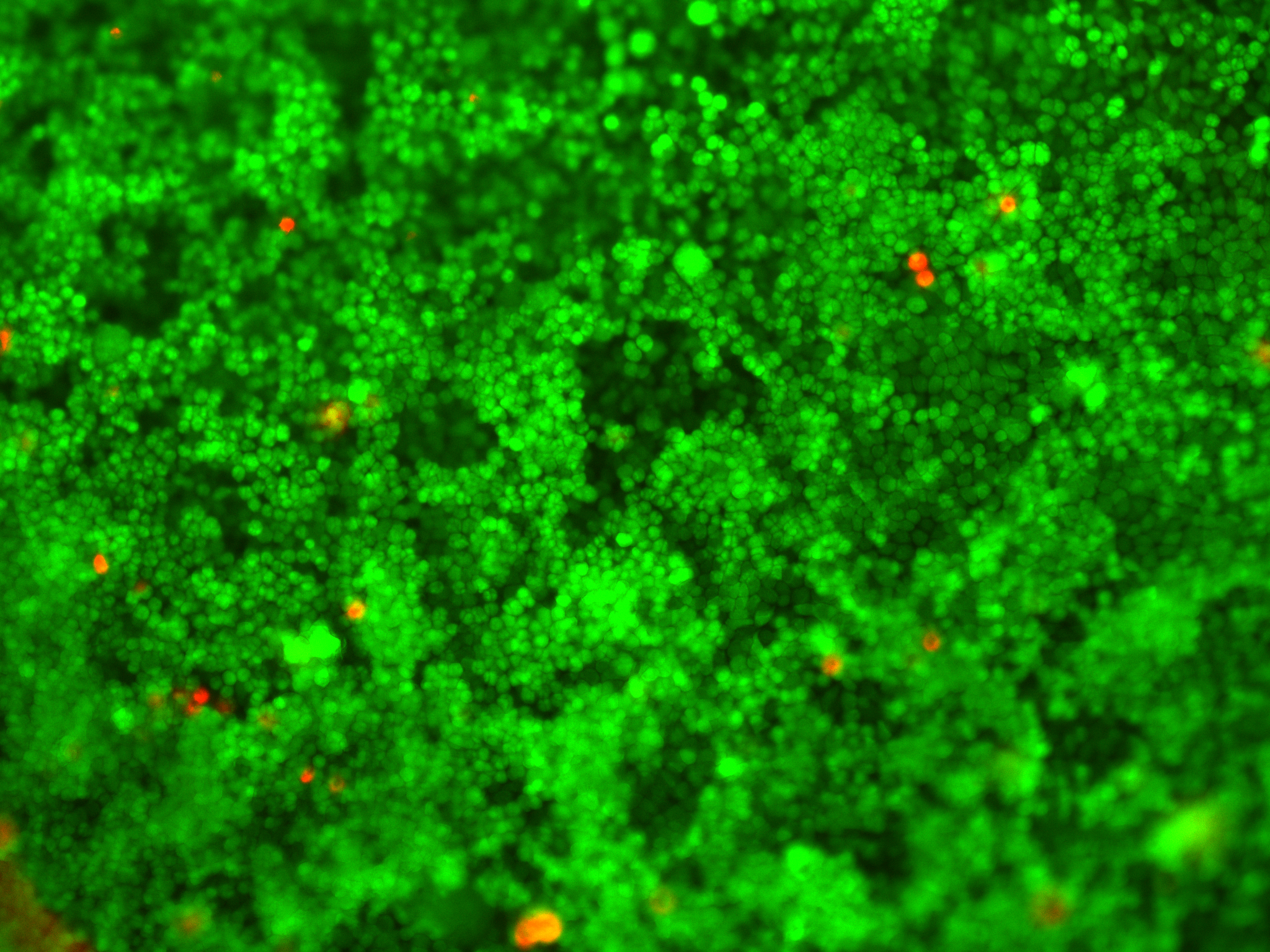

Supplement: Supplementary file 25 — Source Data [file 41467_2025_60928_MOESM25_ESM.zip › Source File/Fig. S37-38/Archive/staining/1023livedead-staining/NdFeB/2.2.tiff]

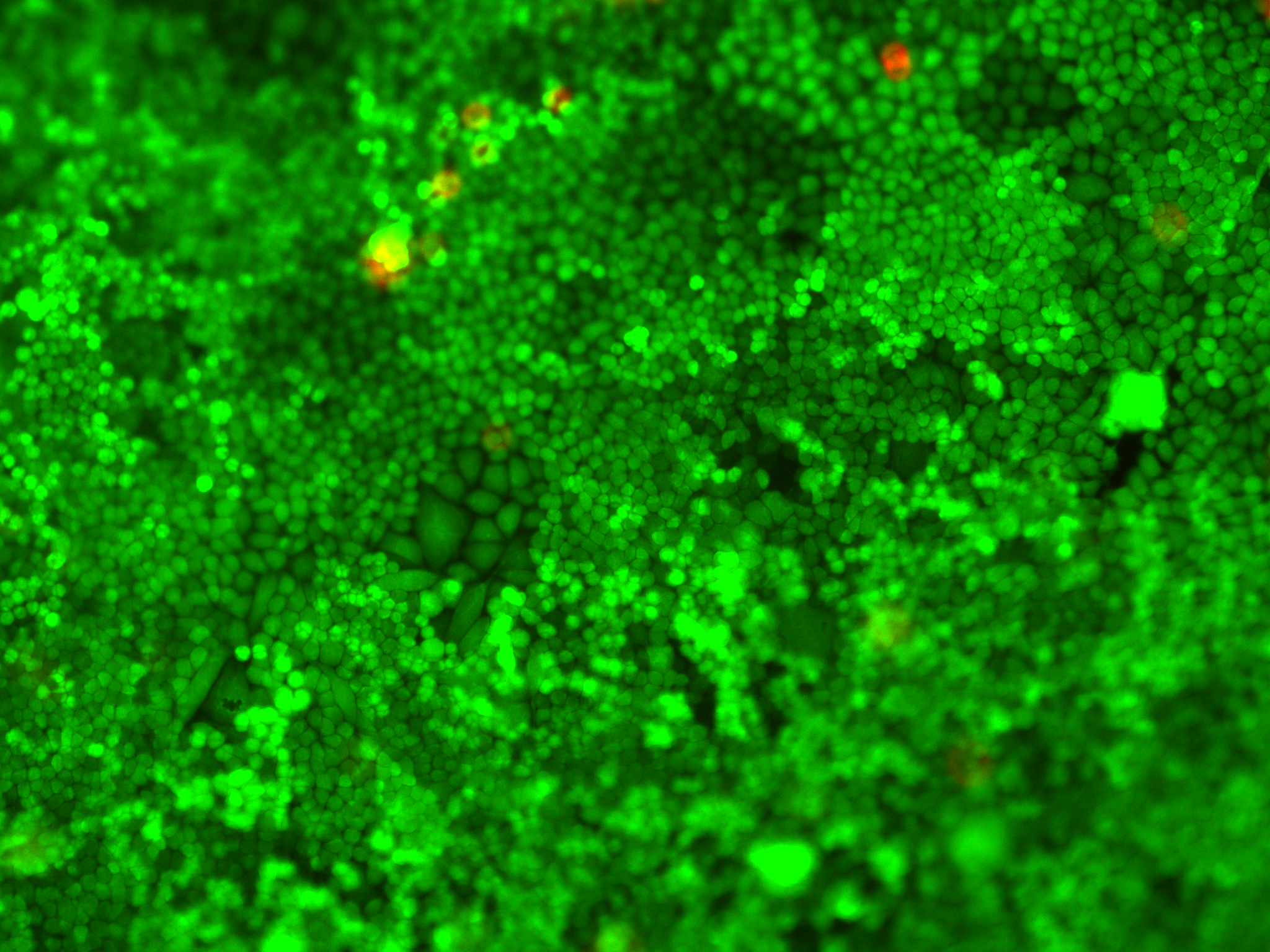

Supplement: Supplementary file 25 — Source Data [file 41467_2025_60928_MOESM25_ESM.zip › Source File/Fig. S37-38/Archive/staining/1023livedead-staining/NdFeB/2.3.tiff]

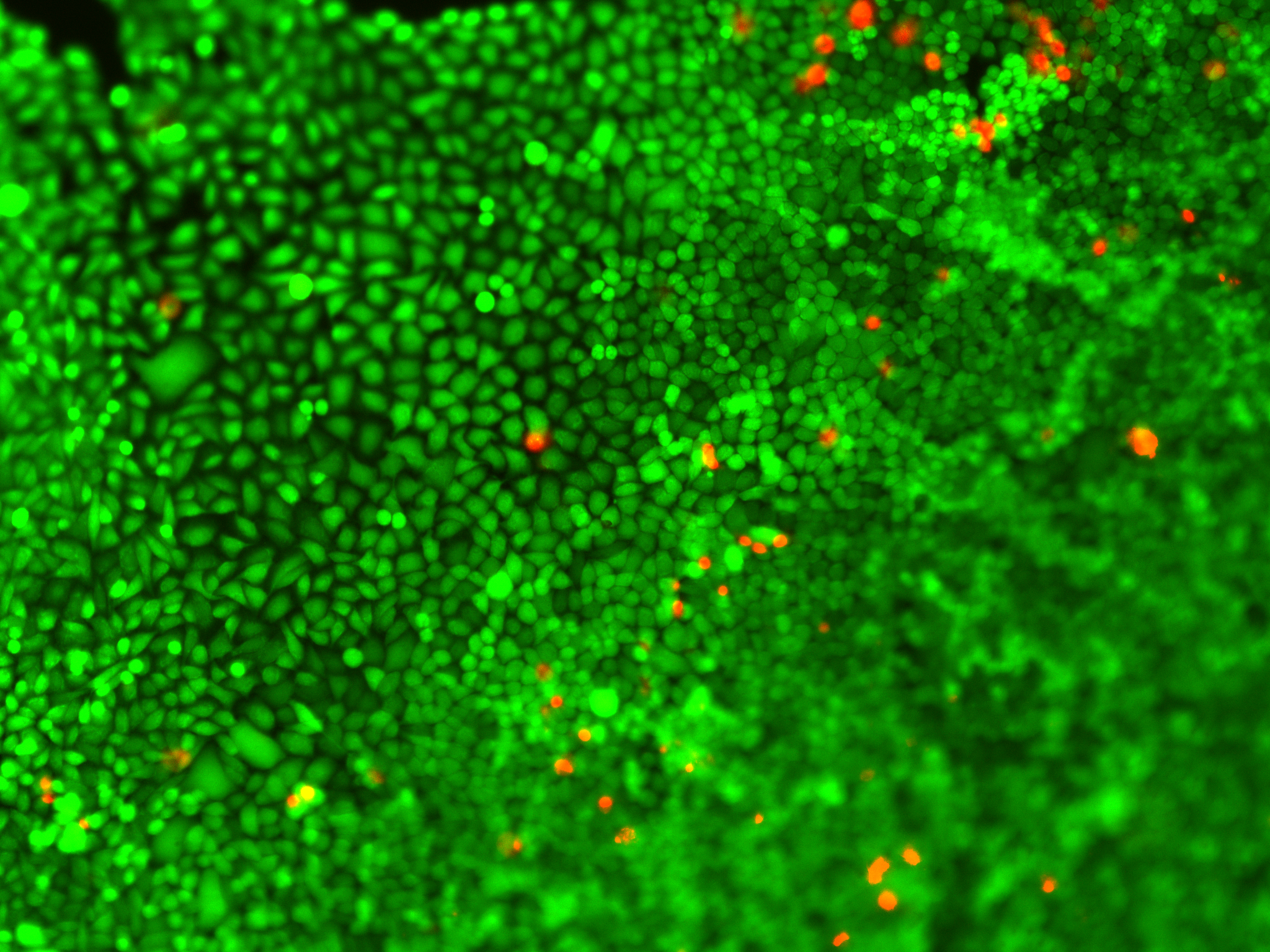

Supplement: Supplementary file 25 — Source Data [file 41467_2025_60928_MOESM25_ESM.zip › Source File/Fig. S37-38/Archive/staining/1023livedead-staining/NdFeB/2.4.tiff]

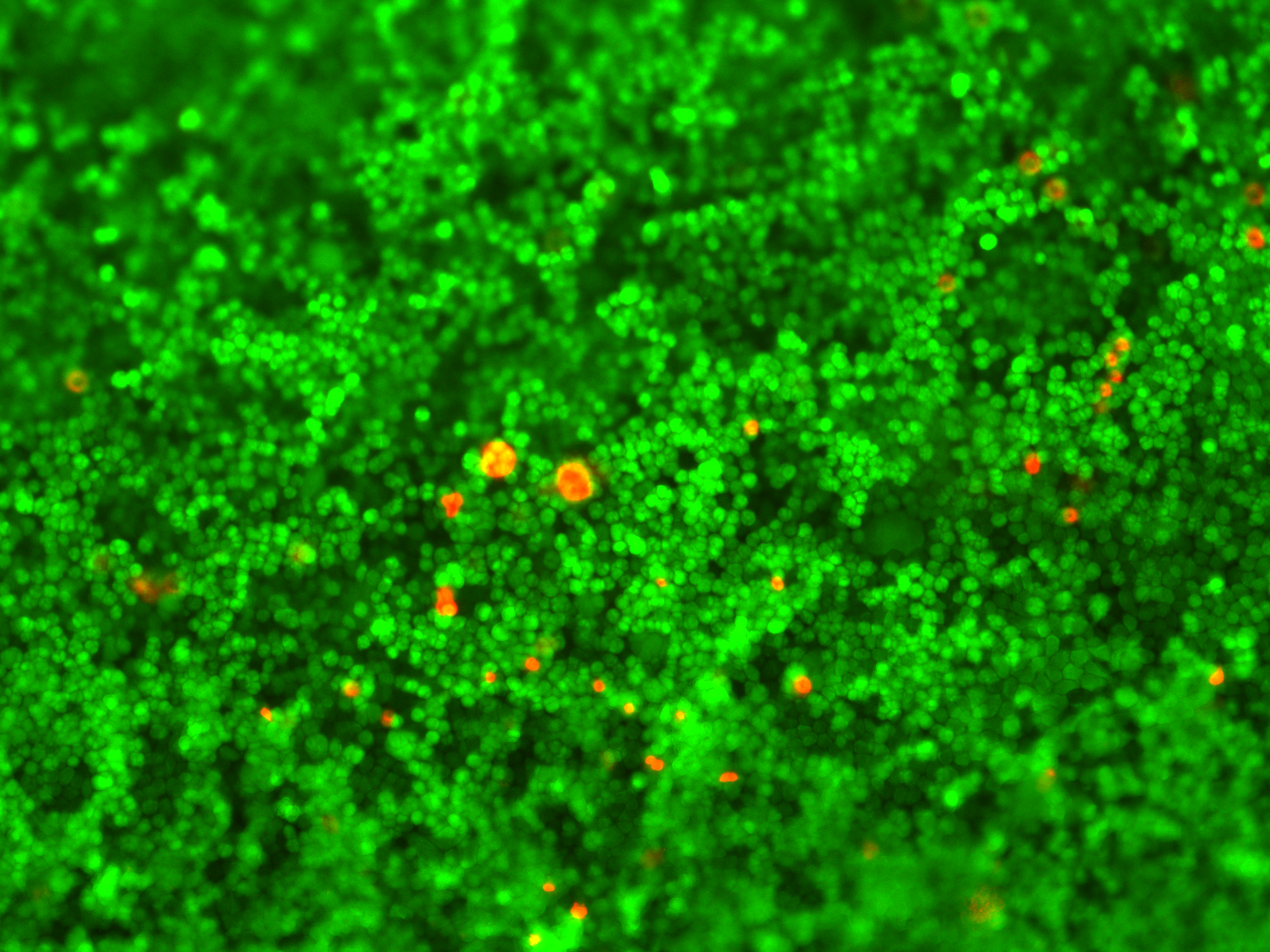

Supplement: Supplementary file 25 — Source Data [file 41467_2025_60928_MOESM25_ESM.zip › Source File/Fig. S37-38/Archive/staining/1023livedead-staining/NdFeB/3.1.tiff]

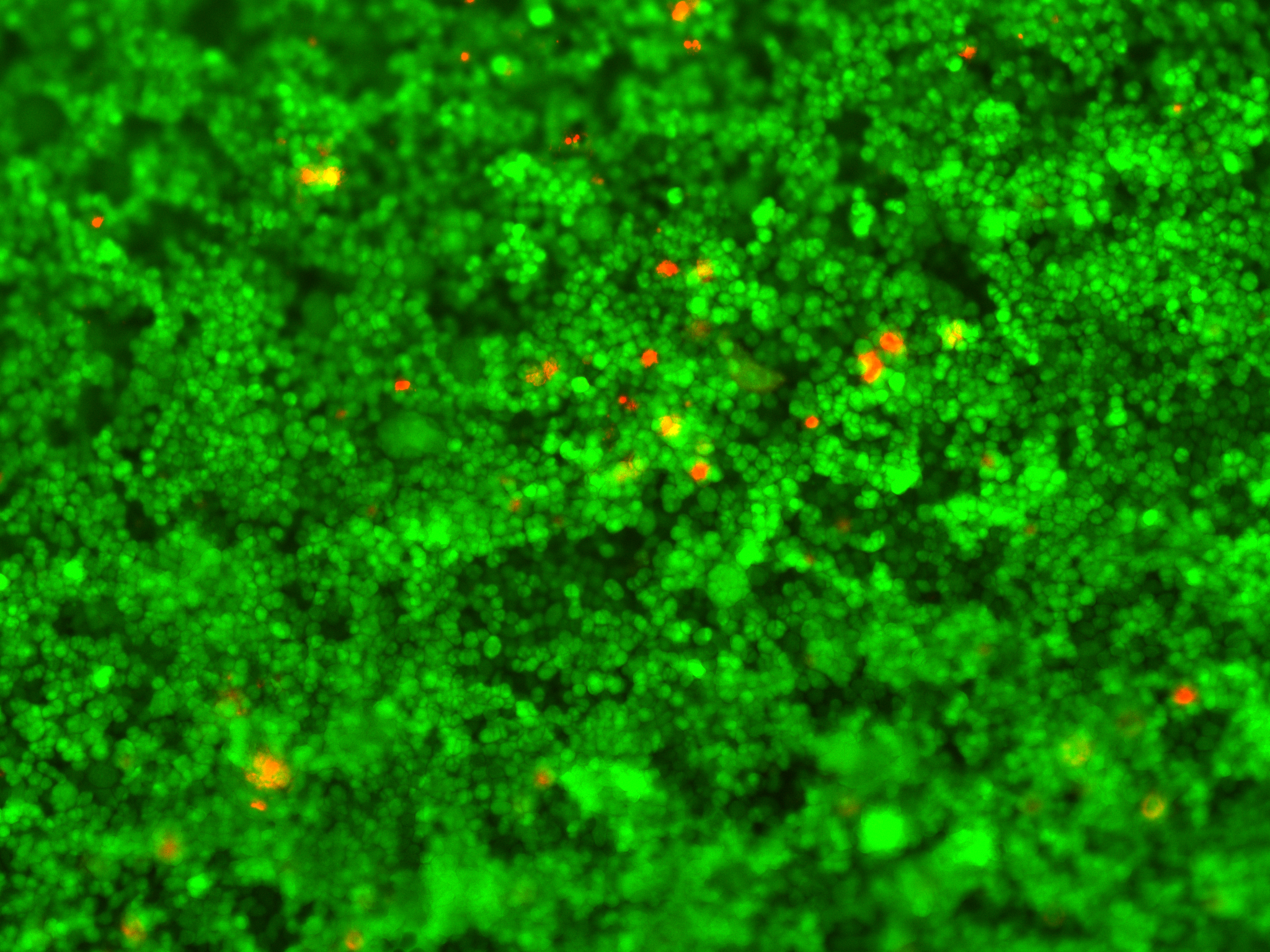

Supplement: Supplementary file 25 — Source Data [file 41467_2025_60928_MOESM25_ESM.zip › Source File/Fig. S37-38/Archive/staining/1023livedead-staining/NdFeB/3.2.tiff]

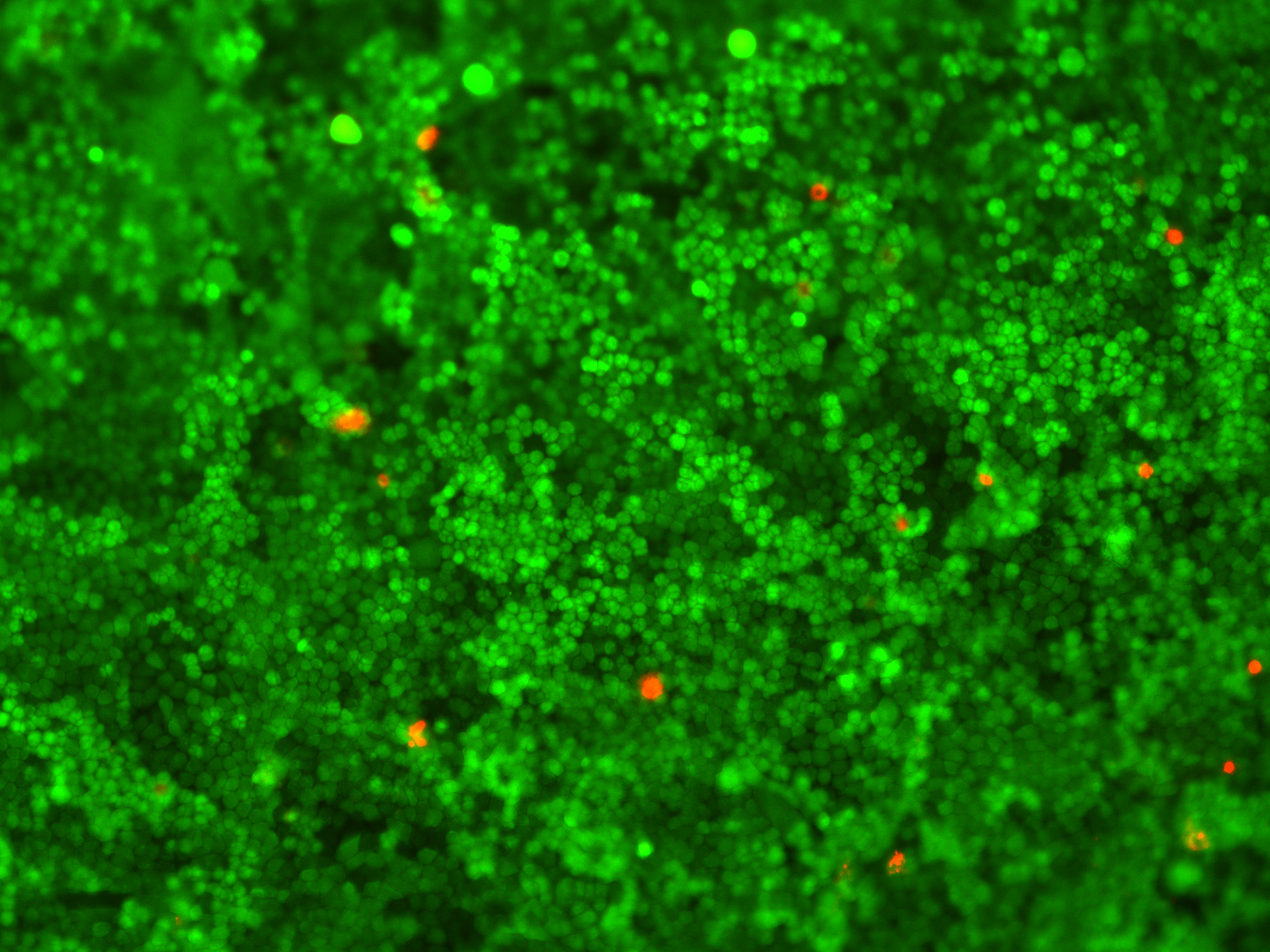

Supplement: Supplementary file 25 — Source Data [file 41467_2025_60928_MOESM25_ESM.zip › Source File/Fig. S37-38/Archive/staining/1023livedead-staining/NdFeB/3.3.tiff]

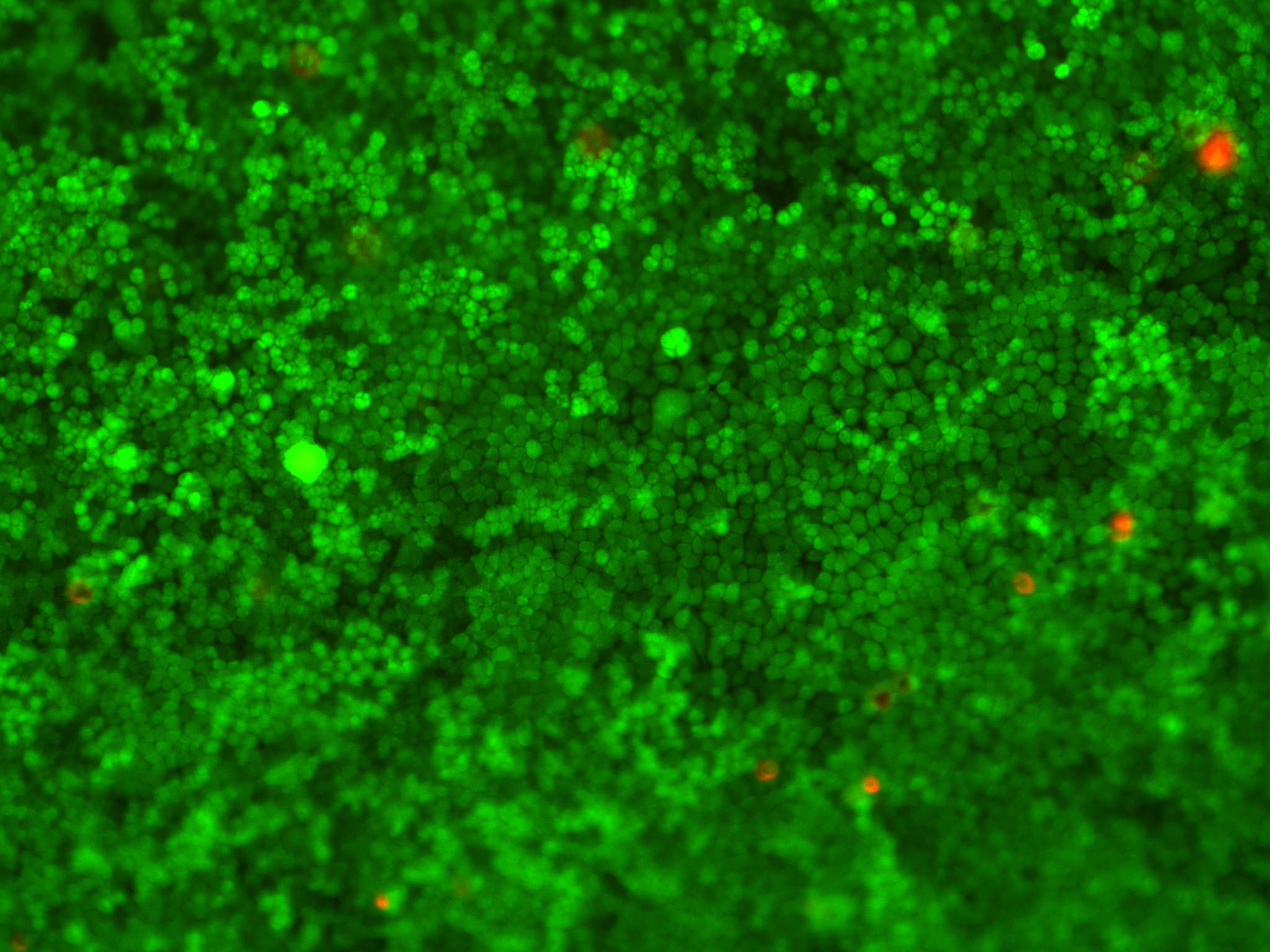

Supplement: Supplementary file 25 — Source Data [file 41467_2025_60928_MOESM25_ESM.zip › Source File/Fig. S37-38/Archive/staining/1023livedead-staining/NdFeB/3.4.tiff]

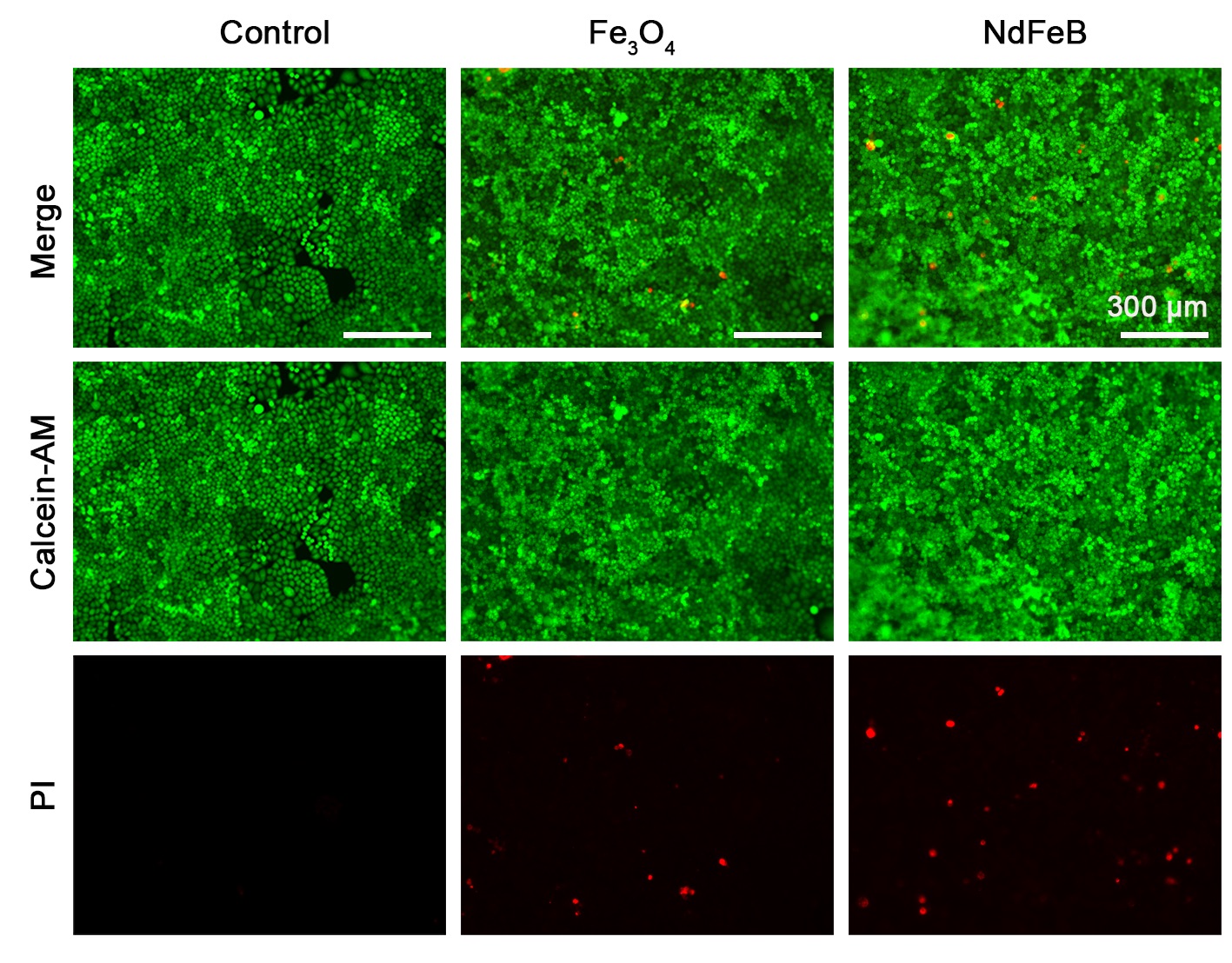

Supplement: Supplementary file 25 — Source Data [file 41467_2025_60928_MOESM25_ESM.zip › Source File/Fig. S37-38/Archive/staining/livedead staining.jpeg]

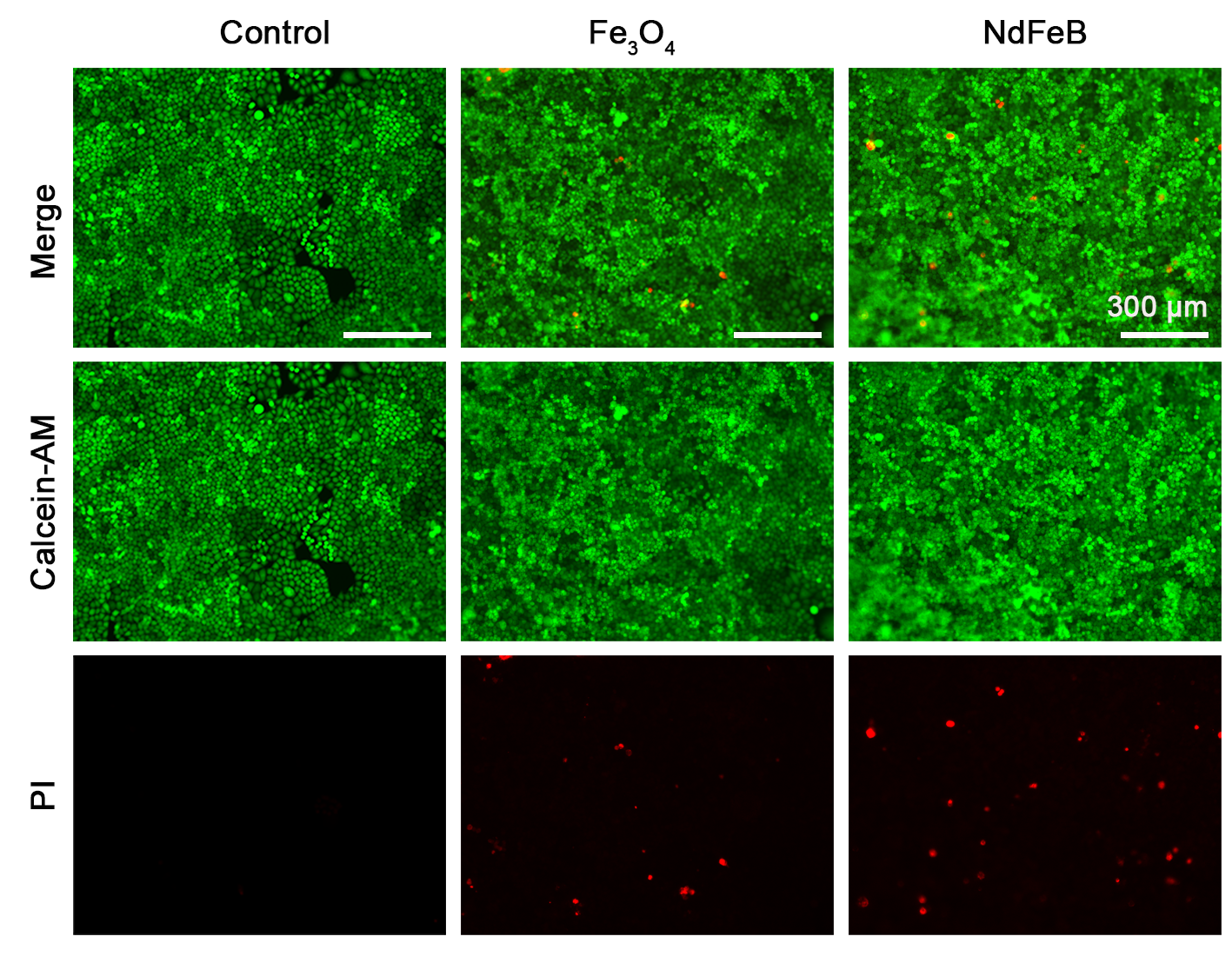

Supplement: Supplementary file 25 — Source Data [file 41467_2025_60928_MOESM25_ESM.zip › Source File/Fig. S37-38/Archive/staining/livedead staining.tif]

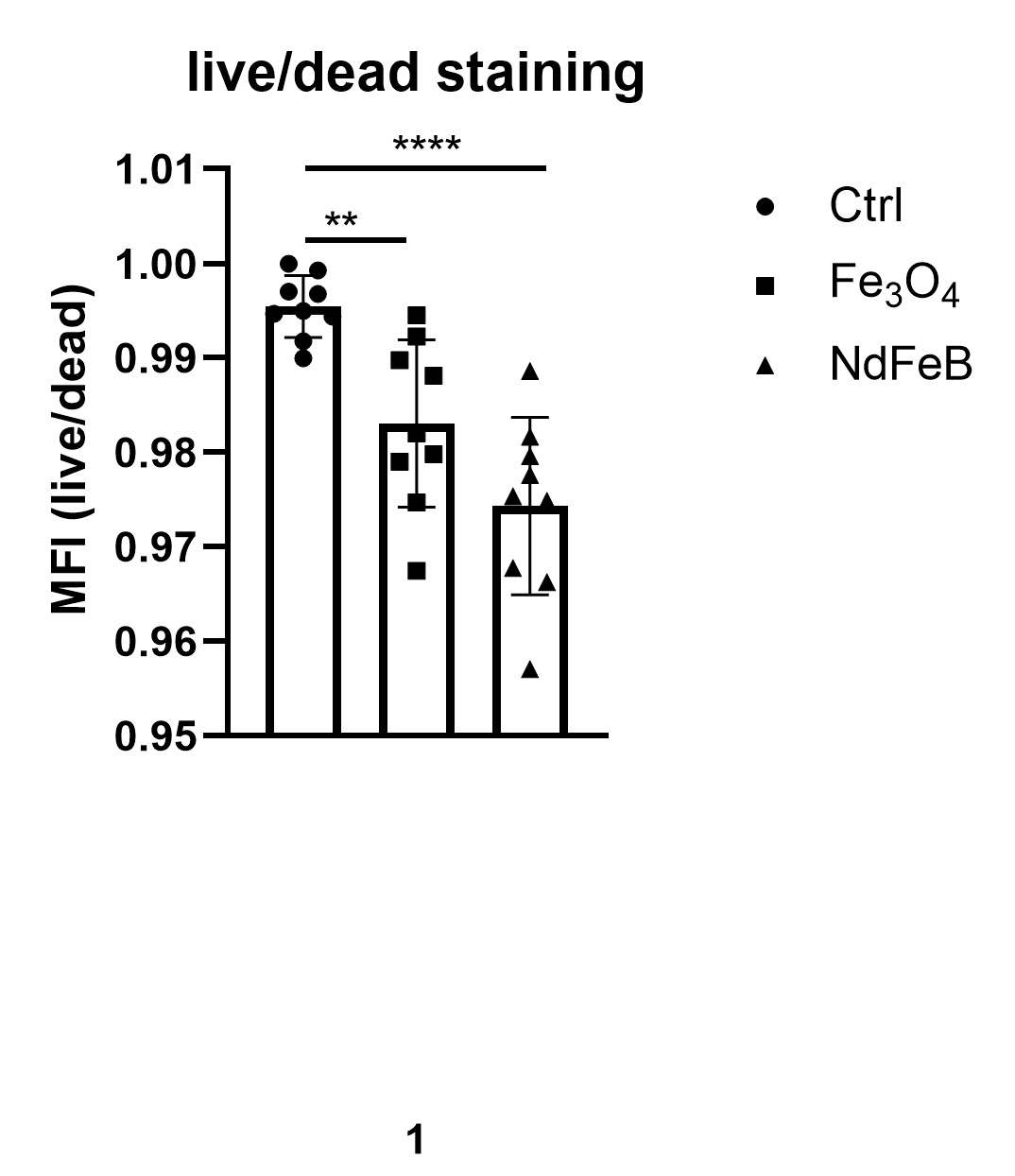

Supplement: Supplementary file 25 — Source Data [file 41467_2025_60928_MOESM25_ESM.zip › Source File/Fig. S37-38/Archive/staining/quantitative analyze.jpeg]

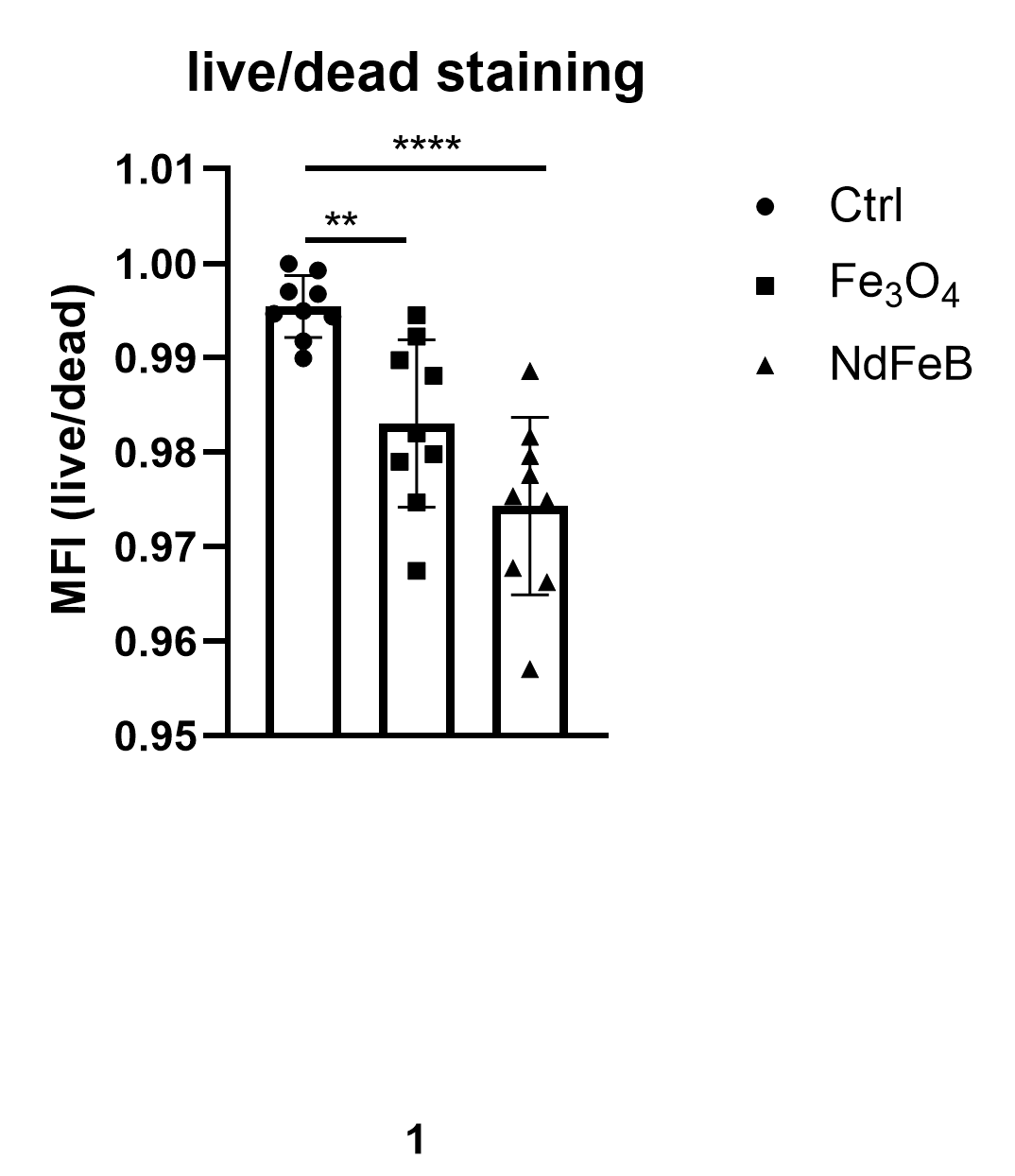

Supplement: Supplementary file 25 — Source Data [file 41467_2025_60928_MOESM25_ESM.zip › Source File/Fig. S37-38/Archive/staining/quantitative analyze.tif]
